# Maternal Sildenafil vs Placebo in Pregnant Women With Severe Early-Onset Fetal Growth Restriction: A Randomized Clinical Trial

**DOI:** 10.1001/jamanetworkopen.2020.5323

**Published:** 2020-06-17

**Authors:** Anouk Pels, Jan Derks, Ayten Elvan-Taspinar, Joris van Drongelen, Marjon de Boer, Hans Duvekot, Judith van Laar, Jim van Eyck, Salwan Al-Nasiry, Marieke Sueters, Marinka Post, Wes Onland, Aleid van Wassenaer-Leemhuis, Christiana Naaktgeboren, Janus C. Jakobsen, Christian Gluud, Ruben G. Duijnhoven, Titia Lely, Sanne Gordijn, Wessel Ganzevoort

**Affiliations:** 1 Department of Obstetrics and Gynecology, Amsterdam UMC, University of Amsterdam, Amsterdam, the Netherlands; 2 Wilhelmina Children’s Hospital, Department of Obstetrics, University Medical Center Utrecht, Gynecology and Neonatology, Utrecht, the Netherlands; 3 Department of Obstetrics and Gynecology, University Medical Center Groningen, Groningen, the Netherlands; 4 Department of Obstetrics and Gynecology, Radboud University Medical Center, Nijmegen, the Netherlands; 5 Department of Obstetrics and Gynecology, Amsterdam UMC, Vrije Universiteit Amsterdam, Amsterdam, the Netherlands; 6 Department of Obstetrics and Gynecology, Erasmus University Medical Center, Rotterdam, the Netherlands; 7 Department of Obstetrics and Gynecology, Maxima Medical Center, Veldhoven, the Netherlands; 8 Department of Obstetrics and Gynecology, Isala Hospital, Zwolle, the Netherlands; 9 Department of Obstetrics and Gynecology, Maastricht University Medical Center, Maastricht, the Netherlands; 10 Department of Obstetrics and Gynecology, Leiden University Medical Center, Leiden, the Netherlands; 11 Department of Obstetrics and Gynecology, Medical Center Leeuwarden, Leeuwarden, the Netherlands; 12 Emma Children’s Hospital, Amsterdam UMC, Department of Neonatology, University of Amsterdam, Amsterdam, the Netherlands; 13 The Copenhagen Trial Unit, Centre for Clinical Intervention Research, Rigshospitalet, Copenhagen University Hospital, Copenhagen, Denmark; 14 Department of Cardiology, Holbæk Hospital, Holbæk, Denmark; 15 Department of Regional Health Research, Faculty of Health Sciences, University of Southern Denmark, Odense, Denmark

## Abstract

**Importance:**

Severe early onset fetal growth restriction caused by placental dysfunction leads to high rates of perinatal mortality and neonatal morbidity. The phosphodiesterase 5 inhibitor, sildenafil, inhibits cyclic guanosine monophosphate hydrolysis, thereby activating the effects of nitric oxide, and might improve uteroplacental function and subsequent perinatal outcomes.

**Objective:**

To determine whether sildenafil reduces perinatal mortality or major morbidity.

**Design, Setting, and Participants:**

This placebo-controlled randomized clinical trial was conducted at 10 tertiary referral centers and 1 general hospital in the Netherlands from January 20, 2015, to July 16, 2018. Participants included pregnant women between 20 and 30 weeks of gestation with severe fetal growth restriction, defined as fetal abdominal circumference below the third percentile or estimated fetal weight below the fifth percentile combined with Dopplers measurements outside reference ranges or a maternal hypertensive disorder. The trial was stopped early owing to safety concerns on July 19, 2018, whereas benefit on the primary outcome was unlikely. Data were analyzed from January 20, 2015, to January 18, 2019. The prespecified primary analysis was an intention-to-treat analysis including all randomized participants.

**Interventions:**

Participants were randomized to sildenafil, 25 mg, 3 times a day vs placebo.

**Main Outcomes and Measures:**

The primary outcome was a composite of perinatal mortality or major neonatal morbidity until hospital discharge.

**Results:**

Out of 360 planned participants, a total of 216 pregnant women were included, with 108 women randomized to sildenafil (median gestational age at randomization, 24 weeks 5 days [interquartile range, 23 weeks 3 days to 25 weeks 5 days]; mean [SD] estimated fetal weight, 458 [160] g) and 108 women randomized to placebo (median gestational age, 25 weeks 0 days [interquartile range, 22 weeks 5 days to 26 weeks 3 days]; mean [SD] estimated fetal weight, 464 [186] g). In July 2018, the trial was halted owing to concerns that sildenafil may cause neonatal pulmonary hypertension, whereas benefit on the primary outcome was unlikely. The primary outcome, perinatal mortality or major neonatal morbidity, occurred in the offspring of 65 participants (60.2%) allocated to sildenafil vs 58 participants (54.2%) allocated to placebo (relative risk, 1.11; 95% CI, 0.88-1.40; *P* = .38). Pulmonary hypertension, a predefined outcome important for monitoring safety, occurred in 16 neonates (18.8%) in the sildenafil group vs 4 neonates (5.1%) in the placebo group (relative risk, 3.67; 95% CI, 1.28-10.51; *P* = .008).

**Conclusions and Relevance:**

These findings suggest that antenatal maternal sildenafil administration for severe early onset fetal growth restriction did not reduce the risk of perinatal mortality or major neonatal morbidity. The results suggest that sildenafil may increase the risk of neonatal pulmonary hypertension.

**Trial Registration:**

ClinicalTrials.gov Identifier: NCT02277132

## Key Points

**Question:**

Does sildenafil reduce the risk of perinatal mortality or morbidity in children of pregnant women with severe early onset fetal growth restriction?

**Findings:**

In this randomized clinical trial including 216 pregnant women, perinatal mortality or major morbidity was not statistically different and occurred in the offspring of 60.2% of participants allocated to sildenafil vs 54.2% of those allocated to placebo. Pulmonary hypertension occurred in 18.8% of neonates in the sildenafil group compared with 5.1% of neonates in the placebo group, which was statistically significantly different.

**Meaning:**

These findings suggest that treatment of severe early onset fetal growth restriction by maternal sildenafil did not reduce the risk of perinatal mortality or major neonatal morbidity, but increased neonatal pulmonary hypertension was observed.

## Teaser

This randomized clinical trial examines whether sildenafil reduces the risk of perinatal mortality or morbidity vs placebo in children of pregnant women with severe early onset fetal growth restriction.

## Introduction

Severe early onset fetal growth restriction is a rare condition, complicating approximately 0.4% of all pregnancies.^1,2^ It is associated with a high risk of fetal death, iatrogenic preterm birth, long-lasting stay at the neonatal intensive care unit, neonatal mortality, and long-term morbidity.^3,4^ Severe early-onset fetal growth restriction is also strongly associated with neurodevelopmental impairment later in childhood.^5,6^ To our knowledge, no effective treatment to promote fetal growth has been identified, and management consists of intensive monitoring to determine the best moment to deliver the fetus, balancing the consequences of prematurity vs undernutrition and hypoxia.^7^

Recently, phosphodiesterase type 5 inhibitors, most often sildenafil, have been investigated as potential treatment for fetal growth restriction.^8,9,10,11,12,13,14,15,16^ The Sildenafil Therapy in Dismal Prognosis Early Onset Fetal Growth Restriction (STRIDER) consortium designed and conducted in synchrony 4 randomized clinical trials to study sildenafil’s hypothesized improvement of placental circulation through its effects on the uteroplacental circulation.^8,9,10,11,12,13,14,15,16^ In the Dutch STRIDER trial, the hypothesis that sildenafil reduces the chance of perinatal mortality and morbidity was tested using a composite outcome of perinatal mortality and major neonatal morbidity.

## Methods

We conducted this placebo-controlled randomized clinical trial in 10 tertiary care centers and 1 general hospital in the Netherlands. Ethical approval was granted by Amsterdam UMC. All participating women provided written informed consent. The protocol was registered on September 29, 2014 (Trial Protocol in Supplement 1), before the first participant was randomized. This study is reported following the Consolidated Standards of Reporting Trials (CONSORT) reporting guideline.

### Study Design

An independent data safety monitoring board (DSMB) monitored the safety of the participants after data were available for each 50 participants, as well as the efficacy after the outcomes were known for half of the participants.^17^ The DSMB charter included the provision to recommend stopping the trial in case the safety of current or future participants was considered to be compromised. Furthermore, a stopping rule was included, indicating that the trial would be stopped if a significant difference between the 2 treatment groups was observed at interim analysis (according to the O’Brien-Fleming spending function, *P* < .005).

### Participants

Pregnant women were eligible if they were between 20 weeks 0 days and 27 weeks 6 days of gestation and if the fetal abdominal circumference was below the 3rd percentile or the estimated fetal weight (EFW) below the 5th percentile, combined with either unilateral or bilateral notching of the uterine artery, Pulsatility Index (PI) of the umbilical artery above the 95th percentile, PI of the middle cerebral artery below the 5th percentile, or a maternal hypertensive disorder. Participants with gestation between 28 weeks 0 days and 29 weeks 6 days were eligible if the EFW was less than 700 g, combined with the aforementioned Doppler anomalies or a maternal hypertensive disorder, to select the patients with unfavorable prognosis. Gestational age estimation was based on a first trimester ultrasound. Exclusion criteria were anticipated imminent termination of pregnancy for maternal or fetal indications, multifetal pregnancy, identified congenital anomalies (affecting outcomes), identified congenital infection, maternal age younger than 18 years, cocaine use, current use of sildenafil, current use of cytochrome P450 3A5 isozyme inhibitors, and recent myocardial infarction or stroke.

Maternal race/ethnicity was collected because maternal race/ethnicity is associated with placental dysfunction and pregnancy outcomes.^18,19^ Whether the maternal race/ethnicity was European descent, African descent, or Asian descent was indicated by the investigator. In case of doubt, the patient was asked to report her race/ethnicity.

In participating centers, samples of maternal blood were collected at randomization by venipuncture and stored at −80 °C for batch testing of placental growth factor (PGF) level. Measurement of PGF level was performed on the Kryptor immunoassay (Thermo Fisher Scientific) and compared with the fifth percentile of a reference population (ie, 106.54 pg/mL).^20^

### Randomization and Masking

The web-based randomization had a 1:1 ratio, random block sizes of 2 to 6, and was stratified per participating center. Participants, clinicians, investigators, and outcome assessors were blinded for the treatment allocation.

### Procedures

Trial medication was manufactured specifically for this trial by Tiofarma, and tablets contained either sildenafil 25 mg or placebo and were taken orally 3 times daily. Active and placebo medications were matching in color, size, weight, and taste. The dosage regimen was based on previous studies by the collaborators on this project.^12,15^

Participants used the trial medication until fetal death, 32 weeks of gestation, or birth. Adherence was participant-reported at each antenatal outpatient clinic visit, Additionally, at the end of the exposure period, medication bottles were collected, and the remaining number of tablets was counted. Participants kept a record of adverse effects. Trial medication was ended at the discretion of the patient. The fetal monitoring (ultrasonography and cardiotocography) and interventions other than the trial medication were at the discretion of the attending gynecologist and in line with Dutch national guidelines and local protocols, depending on the gestational age and EFW. In most participating centers, active management was installed after extensive counseling of parents by a gynecologist and neonatologist and at a minimum gestational age of 26 weeks 0 days combined with an EFW of 500 g. Data were collected from the patient’s electronic health record and entered into a secure electronic database (REDCAP).

### Outcomes

The primary outcome was a composite of either perinatal mortality or major neonatal morbidity before the neonate was discharged from the hospital. Major neonatal morbidity was defined as intraventricular hemorrhage grade 3 or more,^21,22,23^ periventricular leukomalacia grade 2 or more,^24,25^ moderate or severe bronchopulmonary dysplasia,^26,27,28,29,30^ necrotizing enterocolitis Bell stage 2 or more,^31,32^ or retinopathy of prematurity requiring laser therapy.^33,34^ We defined the neonatal period as the time until hospital discharge. Mortality after hospital discharge was considered not to be the mortality of interest in the primary outcome, since the chance of this mortality being associated with the intervention was considered small.

The secondary outcomes were (1) the proportion of mothers experiencing either pre-eclampsia or hemolysis, elevated liver enzymes, and low platelets syndrome^35^; (2) PI of umbilical artery. the first PI measured at the ultrasound performed more than 24 hours after start trial medication; (3) birth weight, with birth weight of live born neonates and birth weight of stillborn fetuses described separately; (4) gestational age at birth or fetal death; and (5) the proportion of neonates with neurodevelopmental impairment at age 2 years, assessed on the 2-year Bayley Scales of Infant Development, Third Edition (BSID-III)^36^ and its cognitive and motor subscales. The latter secondary outcome is not reported here because the 2-year follow-up is not yet complete. When possible, we reported outcomes according to the core outcome set for fetal growth restriction that was developed after the start of the trial.^37^

### Statistical Analysis

We aimed to find a decrease in the incidence of the primary outcome from 71%^15^ in the control group to 56% in the experimental group, which is equal to a relative risk reduction of 21%. Allowing for 10% loss to follow-up and interim analysis for efficacy according to the O’Brien-Fleming spending function (*P* < .005), and with an accepted type I error of 5% and type II error of 80%, we needed to randomize 180 women per group.

The statistical analysis plan, published elsewhere and available in Supplement 1),^38^ provides the details of the statistical analysis. In short, the prespecified primary analysis was an intention-to-treat analysis including all randomized participants. Additionally, several prespecified sensitivity analyses were conducted for the primary outcome: adjusting for gestational age and EFW at randomization; only including participants who had a fetus or neonate without any congenital anomaly that could either explain the small fetal size in hindsight or would have a likely effect on the primary outcome (originally defined as a subgroup analysis, since not all congenital anomalies can be known antenatally); and a per-protocol analysis for the primary outcome that included only participants who used at least 1 tablet of trial medication. Relative risks were calculated using generalized linear models (log link function), and continuous outcomes were analyzed using linear regression.^38^

Predefined subgroup analyses were conducted for participants with a serum level of PGF (categorized as less than the fifth percentile and fifth percentile or higher), gestational age at randomization (categorized as <25 weeks of gestation and ≥25 weeks of gestation), and EFW at randomization (categorized as <300 g, 300 to 599 g, and ≥600 g).

All statistical analyses were conducted independently by 2 researchers (C.N. and R.G.D., supervised by J.C.J.) using R statistical software version 3.5.1 (R Project for Statistical Computing) and SAS statistical software version 9.4 (SAS Institute). *P* values were 2-sided, and statistical significance was set at *P* < .05. Data were analyzed from January 20, 2015, to January 18, 2019.

## Results

Between January 20, 2015 and July 16, 2018, 281 women were eligible, of whom 216 were randomized (Figure). Among these, 108 women were randomized to sildenafil (median gestational age at randomization, 24 weeks 5 days [interquartile range, 23 weeks 3 days to 25 weeks 5 days]; mean [SD] estimate fetal weight, 458 [160] g) and 108 women were randomized to placebo (median gestational age, 25 weeks 0 days [interquartile range, 22 weeks 5 days to 26 weeks 3 days]; mean [SD] estimate fetal weight 464 [186] g). On July 19, 2018, the DSMB recommended to discontinue the trial based on the findings at the interim analysis on the data from the first 183 participants (eAppendix 1 in Supplement 2). The main consideration for the DSMB to recommend stopping was an increased incidence of neonatal pulmonary hypertension (a predefined outcome important for monitoring safety), whereas it was considered unlikely that benefit would be shown on the primary outcome of perinatal mortality or major neonatal morbidity until hospital discharge if the trial were continued to its completion. Moreover, no positive effects on the primary, secondary, or exploratory outcomes, as defined in the statistical analysis plan,^38^ were seen. The results of the recently published STRIDER UK trial^39^ as well as the (at that time unpublished) data of the STRIDER New Zealand/Australia trial^40^ were included in the DSMB deliberations, as was foreseen in the DSMB charter.^17^ The trial leadership stopped the trial immediately on July 19, 2018, at which point 7 remaining participants using trial medication were advised to stop using the trial medication, and drug allocation of all participants was unblinded for the participants and the researchers. Owing to the unforeseen stopping of the trial, we were not able to carry out all analyses as planned (eAppendix 2 in Supplement 2).

**Figure.**
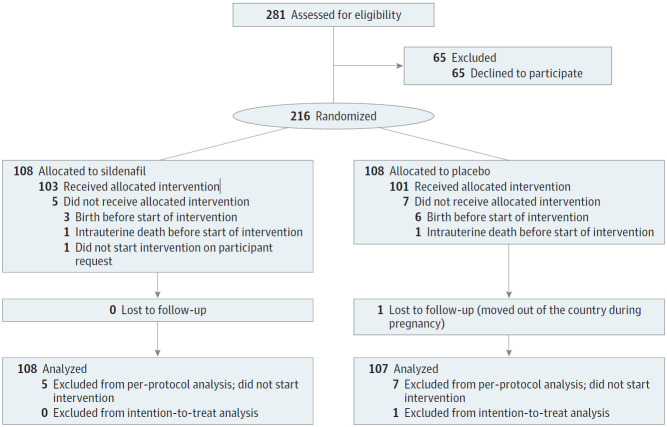
CONSORT Flow Diagram

Of 216 participants randomized at the time of halting the trial, 1 participant was lost to follow-up for all outcomes after having moved abroad, and 12 participants did not start trial medication and were therefore excluded from the per-protocol analysis. The mean (SD) adherence in the per-protocol group was 91% (23%) of the tablets taken.

Baseline characteristics are shown in Table 1. There were no clinically relevant differences between the sildenafil and placebo groups in the maternal or fetal baseline characteristics, other than a slight imbalance in fetal sex (sildenafil: 51 [47.2%] boys; placebo: 59 [54.6%] boys).

**Table 1.** Baseline Characteristics

Characteristic	No. (%)	
	Sildenafil (n = 108)	Placebo (n = 108)
Age, mean (SD), y	31 (5.1)	31 (5.0)
BMI, mean (SD)	26 (4.7)	26 (5.8)
Race/ethnicity, descent		
European	84 (77.8)	86 (79.6)
African	7 (6.5)	11 (10.2)
Asian	2 (1.9)	5 (4.6)
Other	13 (12.0)	6 (5.6)
Maternal smoking	13 (12.0)	10 (9.3)
Gestational age at randomization, median (IQR), wk	24 5/7 (23 3/7 to 25 5/7)	25 0/7 (22 5/7 to 26 3/7)
Ultrasonagraphic examination results, mean (SD)		
Estimated fetal weight, g	458 (160)	464 (186)
Fetal abdominal circumference, mm	165 (2)	164 (26)
Sex		
Boys	51 (47.2)	59 (54.6)
Girls	57 (52.8)	48 (44.4)
Notching uterine artery (1- or 2-sided)	61 (56.5)	64 (59.3)
PI		
Umbilical artery >95th percentile	51 (47.2)	53 (49.1)
Middle cerebral artery <5th percentile	47 (43.5)	43 (39.8)
End-diastolic flow		
Positive	73 (67.6)	65 (60.2)
Absent	27 (25.0)	33 (30.6)
Reversed	7 (6.5)	7 (6.5)
Pregnancy hypertension	22 (20.4)	24 (22.2)
Pre-eclampsia	23 (21.3)	26 (24.1)
HELLP syndrome	1 (0.9)	2 (1.9)
Blood pressure, mean (SD), mm Hg		
Systolic	132 (22)	132 (20)
Diastolic	83 (15)	83 (15)
PGF <5th percentile of reference value, No./total No. (%)^a^	56/61 (91.8)	53/59 (89.8)

Abbreviations: BMI, body mass index (calculated as weight in kilograms divided by height in meters squared); HELLP, hemolysis, elevated liver enzymes, and low platelets; IQR, interquartile range; PI, Pulsatility Index; PGF, placental growth factor.

^a^ Reference value was 106.54 pg/L; 5th percentile of reference population.

No difference was observed in the composite primary outcome of perinatal mortality or major neonatal morbidity until hospital discharge: 65 participants (60.2%) in the sildenafil-group and 58 participants (54.2%) in the placebo-group experienced perinatal death or major neonatal morbidity (relative risk [RR], 1.11; 95% CI, 0.88-1.40; *P* = .38) (Table 2). Bayes factor analysis indicated that the results on the primary outcome were 3.7-fold more likely compatible with no effect than with the risk reduction hypothesized in the sample size calculation. No differences were observed in the subcomponents of the primary outcome. Perinatal mortality was comparable (sildenafil: 44 deaths [40.7%]; placebo: 40 deaths [37.4%]; RR, 1.09; 95% CI, 0.78-1.52; *P* = .61). Two more children in the sildenafil group died after hospital discharge: 1 died 1 day after hospital discharge owing to sepsis resulting from necrotizing enterocolitis; another died at age 18 months owing to cardiogenic shock resulting from sepsis. Trial sequential analysis on the data from this trial showed that the boundary for futility was crossed for the primary outcome (eAppendix 3 in Supplement 2).

**Table 2.** Fetal or Neonatal Outcomes (Intention-to-Treat Analysis)^a^

Outcome	No./total No. (%)		RR (95% CI)	Mean difference (95% CI)	*P* value
	Sildenafil	Placebo			
Primary outcome^b^	65/108 (60.2)	58/107 (54.2)	1.11 (0.88 to 1.40)	NA	.38
Birth weight, mean (SD), g	829 (537)	884 (627)	NA	−55 (−211 to 101)	.49
Stillbirth	23/108 (21.3)	29/107 (27.1)	0.79 (0.49 to 1.27)	NA	.32
Birth weight, mean (SD), g	414 (143)	362 (115)	NA	53 (−17 to 123)	.15
Percentile^c^					
<Tenth	22/22 (100)	25/25 (100)	NA	NA	NA
<Third	21/22 (95.5)	24/25 (96.0)	0.99 (0.88-1.12)	NA	>.99
Live birth	85/108 (78.7)	78/107 (72.9)	1.08 (0.93 to 1.26)	NA	.32
Birth weight, mean (SD), g	942 (549)	1078 (628)	NA	−136 (−318 to 44)	.14
Percentile^c^					
<Tenth	83/85 (97.6)	78/78 (100.0)	0.98 (0.95-1.01)	NA	.50
<Third	77/85 (90.6)	69/78 (88.5)	1.02 (0.92-1.14)	NA	.80
Apgar score 5 min <7	32/108 (37.6)	25/107 (32.1)	1.17 (0.77 to 1.79)	NA	.46
Cord blood gas pH <7.10	2/48 (4.2)	0/31 (0)			
Neonatal death	21/85 (24.7)	11/78 (14.1)	1.75 (0.9 to 3.39)	NA	.10
Survival					
At hospital discharge	64/85 (75.3)	67/78 (85.9)	0.88 (0.75 to 1.02)	NA	.09
With major morbidity at hospital discharge	21/85 (24.7)	18/78 (23.1)	1.07 (0.62 to 1.85)	NA	.81
Without major morbidity at hospital discharge	43/85 (50.6)	49/78 (62.8)	0.81 (0.61 to 1.06)	NA	.12
Postmenstrual age at first discharge home, mean (SD), wk	42 (7.9)	40 (2.3)	NA	2.04 (0.04 to 4.03)	.047
IVH grade III or IV	3/85 (3.5)	2/78 (2.6)	1.38 (0.24 to 8.02)	NA	.72
PVL grade II or more	0/85 (0)	0/78 (0)	NA		>.99
BPD					
Moderate or severe	23/85 (27.1)	16/78 (20.5)	1.32 (0.75 to 2.31)	NA	.33
None	41/85 (48.2)	47/78 (60.3)	0.80 (0.61 to 1.06)	NA	.13
ROP treated by laser or surgery	8/85 (9.5)	3/78 (3.8)	2.45 (0.67 to 8.9)	NA	.17
1 or more culture-proven episode of infection or clinical episode of infection with antibiotic treatment necessary ≥5 d	44/85 (51.8)	35/78 (44.9)	1.15 (0.84 to 1.59)	NA	.38
NEC grade II or more	7/85 (8.3)	8/78 (10.3)	0.80 (0.31 to 2.11)	NA	.66
Abdominal circumference, initial growth rate between randomization and 14 d, mean (SD), mm/wk	7.4 (6.2)	8.8 (7.3)	NA	−1.4 (−3.43 to 0.69)	.19

Abbreviations: BPD, bronchopulmonary dysplasia; EFW, estimated fetal weight; IVH, intraventricular hemorrhage; NA, not applicable; NEC, necrotizing enterocolitis; PVL, periventricular leukomalacia; ROP, retinopathy of prematurity; RR, relative risk.

^a^ Intention-to-treat analysis corrected for gestational age and EFW at randomization, and per-protocol analysis had similar results.

^b^ The primary outcome was perinatal death or major neonatal morbidity before discharge.

^c^ Birth weight percentiles were only calculated for infants born after 23 weeks gestational age.

The proportion of mothers experiencing either pre-eclampsia or hemolysis, elevated liver enzymes, and low platelets syndrome was 46 mothers (42.6%) in the sildenafil group vs 48 mothers (44.9%) in the group allocated to placebo (RR, 0.95; 95% CI, 0.70-1.29) (Table 3). The mean PI of the maternal uterine artery or the fetal umbilical and middle cerebral artery arteries after treatment with study medication did not differ between groups (eTable 1 in Supplement 2).

**Table 3.** Maternal Outcomes (Intention-to-Treat Analysis)^a^

Outcome	No./total No. (%)		RR (95% CI)	Mean difference (95% CI)	*P* value
	Sildenafil	Placebo			
Treatment duration, mean (SD), d	24/108 (18)	20/107 (43)	NA	3.73 (−5.44 to 12.91)	.43
Gestational age at birth, mean (SD), wk	29 3/7 (4 0/7)	29 3/7 (4 3/7)	NA	−0.01 (−1.12 to 1.11)	.99
Preterm birth, wk					
<28	46/108 (42.6)	48/107 (44.9)	0.95 (0.70 to 1.29)	NA	.74
<37	97/108 (89.8)	90/107 (84.1)	1.07 (0.96 to 1.18)	NA	.22
Pregnancy prolongation after randomization, mean (SD), d	34 (28)	33 (32)	NA	0.64 (−7.4 to 8.67)	.88
Mode of delivery					.46
Cesarean					NA
On fetal indication	57/108 (52.8)	51/107 (47.7)	NA	NA	
On maternal indication	14/108 (13.0)	14/107 (13.1)	NA	NA	
Induced vaginal birth					
On fetal indication	7/108 (6.5)	12/107 (11.2)	NA	NA	
On maternal indication	8/108 (7.4)	3/107 (2.8)	NA	NA	
Spontaneous vaginal birth	9/108 (8.3)	11/107 (10.3)	NA	NA	
Induction of labor after intrauterine death	12/108 (11.1)	16/107 (15.0)	NA	NA	
Pregnancy hypertension	74/108 (68.5)	72/107 (67.3)	1.02 (0.85 to 1.22)	NA	.85
Pre-eclampsia	42/108 (38.9)	44/107 (41.1)	0.95 (0.68 to 1.31)	NA	.74
HELLP syndrome	12/108 (11.1)	10/107 (9.3)	1.19 (0.54 to 2.63)	NA	.67
Maternal use of antihypertensive treatment antenatal or postnatal, No. used					
None	50/108 (46.3)	53/107 (49.5)	NA	NA	.61
1	32/108 (29.6)	34/107 (31.8)	NA	NA	
2	16/108 (14.8)	15/107 (14.0)	NA	NA	
≥3	10/108 (9.3)	5/107 (4.7)	NA	NA	
Maternal magnesium sulphate for hypertension	14/108 (13.0)	15/107 (14.0)	0.92 (0.47 to 1.82)	NA	.82
Antenatal corticosteroids for fetal lung maturation before birth					
48 h to 14 d (complete course)	52/85 (61.2)	56/78 (71.8)	0.85 (0.68 to 1.06)	NA	.15
<48 h (incomplete course)	4/85 (4.8)	7/78 (9.0)	0.52 (0.16 to 1.72)	NA	.29

Abbreviation: NA, not applicable; HELLP, hemolysis, elevated liver enzymes, and low platelets; RR, relative risk.

^a^ Intention-to-treat analysis corrected for gestational age and estimated fetal weight at randomization. Per-protocol analysis had similar results.

No difference in birth weight was observed between the treatment groups. Mean (SD) birth weight of the neonates who were stillborn was 414 (143) g in the sildenafil group vs 362 (115) g in the placebo group (*P* = .15). Mean (SD) gestational age of birth or fetal death was 29 weeks 3 days (4 weeks 0 days) in the sildenafil group vs 29 weeks 3 days (4 weeks 3 days) in the placebo group (*P* > .99).

The results of all exploratory outcomes are reported in Table 2 and Table 3. Because the DSMB based their advice to stop the trial on the increased occurrence of neonatal pulmonary hypertension in the sildenafil group, we composed an expert adjudication committee of 4 neonatologists (W.O., A.F.J.v.H., I.K.M.R., and E.L.) experienced in treating neonates who are preterm and growth restricted and a pediatric cardiologist (R.M.F.B.) knowledgeable on the subject of neonatal and pediatric pulmonary hypertension to carefully review this outcome. The committee, blinded for treatment allocation, reviewed all neonatal records with the purpose of consensus validation of this diagnosis after discontinuation of the trial. Persistent pulmonary hypertension was defined as either confirmed by cardiac ultrasonagraphic examination or as a difference in oxygen saturation between the right upper extremity and either lower extremity (ie, postductal) of more than 10%. There was an increase of neonates in the sildenafil group who experienced pulmonary hypertension vs the placebo group (16 of 85 neonates [18.8%] vs 4 of 78 neonates [5.1%]; RR, 3.67; 95% CI, 1.28-10.51; *P* = .008). The adjudication committee observed that 2 different forms of pulmonary hypertension had occurred (eTable 2 in Supplement 2), including persistent pulmonary hypertension in the neonate and later-onset pulmonary hypertension associated with either sepsis or bronchopulmonary dysplasia. Neonatal death was attributable to pulmonary hypertension in 4 infants, 2 in each group (eTable 2 and eTable 3 in Supplement 2). We plan to publish a separate article on the diagnosis of pulmonary hypertension and the validation process within the trial.

No subgroup differences were detected when assessed by adding interaction terms to the models, and the sensitivity analyses did not lead to statistically significant results on the primary outcome (Table 4). However, the RR slightly increased when analyses were adjusted for gestational age and EFW at inclusion (RR, 1.23; 95% CI, 0.69-2.23; *P* = .48) and in a post hoc analysis that explored the potential effect of the imbalance of sex at inclusion (RR, 1.33; 95% CI, 0.77-2.30; *P* = .31).

**Table 4.** Prespecified Sensitivity and Subgroup Analyses on the Primary Outcome According to Treatment

Analysis	No./total No. (%)		RR (95% CI)	*P* value^a^
	Sildenafil	Placebo		
Sensitivity				
Per protocol	60/103 (58.3)	54/100 (54.0)	1.08 (0.85-1.38)	.54
Adjusted for EFW and GA at randomization	65/108 (60.2)	58/107 (54.2)	1.23 (0.69-2.23)	.48
Post hoc adjustment for sex	65/108 (60.2)	58/107 (54.2)	1.33 (0.77-2.30)	.31
Excluding				
Neonates who appeared to have a congenital anomaly	60/102 (58.8)	54/101 (53.5)	1.10 (0.86-1.40)	.44
Participants who were pregnant or for whom the neonate was admitted at NICU when trial was stopped (post hoc)	60/97 (61.9)	55/98 (56.1)	1.10 (0.87-1.39)	.42
Placental growth factor, percentile of the reference value				
<Fifth	36/56 (64.3)	25/53 (47.2)	1.36 (0.96-1.93)	.99
≥Fifth	4/5 (80.0)	3/6 (50.0)	1.60 (0.64-3.98)	
GA at randomization, wk				
<25	42/60 (70.0)	36/54 (66.7)	1.05 (0.82-1.35)	.85
≥25	23/48 (47.9)	22/53 (41.5)	1.15 (0.75-1.78)	
EFW at randomization, g				
<300	15/19 (78.9)	21/26 (80.8)	0.98 (0.73-1.32)	.70
300-599	44/67 (65.7)	23/48 (47.9)	1.37 (0.97-1.93)	
≥600	3/18 (16.7)	10/26 (38.5)	0.43 (0.14-1.36)	

Abbreviations: EFW, estimated fetal weight; GA, gestational age; NICU, neonatal intensive care unit; RR, relative risk.

^a^ The *P* value is from the interaction term for subgroup analyses and from the treatment effect for the sensitivity analyses.

We observed 10 maternal and 2 fetal or neonatal serious adverse events in the sildenafil group vs 8 maternal and 2 fetal or neonatal serious adverse events in the placebo group that could be directly attributed to the high-risk nature of the study population (eg, hospitalization of the neonate) (eTable 4 in Supplement 2). Other than pulmonary hypertension, there were no differences in the other serious adverse events. Several adverse effects of the trial medication with different frequencies were reported by the participants and are presented in eTable 5 in Supplement 2. The primary causes of neonatal death and the congenital anomalies observed are described in eTable 6 and eTable 7 in Supplement 2.

## Discussion

This randomized clinical trial found that sildenafil compared with placebo did not reduce the risk of perinatal mortality or major neonatal morbidity. This finding is in line with 2 other published STRIDER trials from the UK^39^ and from New Zealand and Australia,^40^ and is confirmed by trial sequential analysis of this outcome that includes all 3 trials.

Our present finding of an increased incidence of pulmonary hypertension after antenatal sildenafil administration was not observed in the 2 other STRIDER trials.^39,40^ This difference may be explained by definition differences, diagnostic strategies, or thresholds of suspicion. Our finding may indicate an important safety signal, and causality is a possibility because sildenafil targets the pulmonary vasculature. We hypothesize that the causal mechanisms might be rebound vasoconstriction (or lack of dilatation) of the pulmonary arteries to structural changes within the pulmonary vasculature.

The Canadian STRIDER trial (NCT02442492) was terminated based on the results of this STRIDER trial. The planned individual patient data analysis that combines our data with all other STRIDER trials will have more power to draw conclusions based on all available data and hopefully to allow meaningful subgroup analysis to identify if there are specific participant groups that experience harm or benefit from the intervention.^41^

### Limitations

Our trial has limitations. First, the trial was stopped before the planned sample size was reached because of an increased incidence of pulmonary hypertension in the sildenafil group, as well as indications of futility in our primary outcome. Pulmonary hypertension was predefined as an important safety outcome to monitor, but it was neither defined as a primary or secondary outcome, and pulmonary hypertension is a nonvalidated surrogate outcome regarding more patient-centered outcomes. Although each adverse event should be seriously regarded,^42^ it might be argued that the pulmonary hypertension result should only be regarded as hypothesis-generating and should be tested in the planned individual patient data analysis.^41^ Second, when assessing the primary outcome of perinatal mortality or major neonatal morbidity, the trial sequential analysis showed that the boundary for futility was crossed, indicating that we could reject that sildenafil reduces the risk of the primary outcome by 20%. However, we cannot reject that sildenafil reduces the risk of the primary outcome by smaller and perhaps clinically important margins, or that sildenafil reduces the risk of any of the secondary outcomes.

It could be argued that the trial was stopped too soon owing to the lack of robustness on the findings of harm. However, the advice of the independent DSMB was not only based on potential harms but also on lack of benefits. In addition to the increase in pulmonary hypertension observed at the interim analysis, it became evident that it was unlikely that benefit of sildenafil treatment would be shown on the primary outcome if the trial were continued to its completion. This was also demonstrated in the trial sequential analysis on the primary outcome that showed that the boundary for futility was crossed when taking the results of the UK^39^ and Australian/New Zealand^40^ STRIDER trials into account.

It could also be argued that the STRIDER trials were premature. However, there was extensive evidence in appropriate animal models of fetal growth restriction and increasing human evidence suggesting potential for a positive effect on fetal growth.^8^ The dosage used in this study (ie, 25 mg 3 times daily) was based on a previous trial^15^ and is slightly higher than the dosage used for the treatment of pulmonary hypertension in adults. A meta-analysis of animal studies^8^ suggested that a higher dosage than the current study used might be necessary to reach adequate serum levels of sildenafil. Sildenafil is approved to improve exercise ability and delay clinical worsening of pulmonary arterial hypertension in adult patients (World Health Organization Group I). In 2012, the US Food and Drug Administration recommended that sildenafil should not be prescribed to children ages 1 through 17 years for pulmonary arterial hypertension.^43^ However, there were no reported safety concerns for the use of sildenafil in fetal growth restriction. In contrast, there was an ongoing inclusion of this drug into clinical practice in this at-risk patient category.^44^ Adequately powered randomized clinical trials are necessary to assess the validity of an intervention before it is implemented. The concerted approach of the STRIDER trials aimed to prevent premature implementation of sildenafil based a few underpowered trials and sought to thoroughly test the beneficial claims before implementation.^12,14,15^

## Conclusions

This randomized clinical trial found that antenatal maternal sildenafil administration for severe early onset fetal growth restriction did not reduce the risk of perinatal death or major neonatal morbidity. Our results suggest that sildenafil may increase the risk of neonatal pulmonary hypertension.

## Notes

Supplement 1.
Trial Protocol and Statistical Analysis Plan

Supplement 2. **eAppendix 1.** Letter of DSMB Regarding Result of Interim Analysis **eAppendix 2.** Differences Between the Predefined Statistical Analysis Plan and the Final Analysis **eAppendix 3.** Trial Sequential Analyses **eTable 1.** Doppler Measurements at Randomization and First Measurement More Than 24 Hours After Start of Medication **eTable 2. **Types of Pulmonary Hypertension Within Live Born Neonates in the Dutch STRIDER Trial per Randomization Allocation **eTable 3.** Characteristics of Neonates With and Without Pulmonary Hypertension **eTable 4. **Non–Context Specific Serious Adverse Events **eTable 5. **Side Effects of Study Medication as Reported by the Participants **eTable 6.** Primary Causes of Neonatal Death **eTable 7.** Congenital Anomalies
eReferences

Supplement 3.
Dutch STRIDER Trial Group Members

Supplement 4.
Data Sharing Statement
