# Errors in Table 2

**DOI:** 10.1001/jamanetworkopen.2025.33037

**Published:** 2025-08-21

In the Original Investigation titled “Maternal Sildenafil vs Placebo in Pregnant Women With Severe Early-Onset Fetal Growth Restriction: A Randomized Clinical Trial,”^1^ published June 17, 2020, there were missing data and errors in the numbers and percentages of birth weight percentiles in Table 2. In addition, Supplement 3 was added to acknowledge the contributions of nonauthor collaborators. This article has been corrected.^1^
