# Supplementary material for: Maternal Sildenafil vs Placebo in Pregnant Women With Severe Early-Onset Fetal Growth Restriction: A Randomized Clinical Trial
Source: JAMA Netw Open. 2020 Jun 17;3(6):e205323. doi: 10.1001/jamanetworkopen.2020.5323 (PMC7301225; doi:10.1001/jamanetworkopen.2020.5323)
Supplement: Supplement 1. — Trial Protocol and Statistical Analysis Plan [file jamanetwopen-e205323-s001.pdf]

OnderzoeksPROTOCOL The Dutch STRIDER

(Sildenafil TheRapy In Dismal prognosis Early-onset fetal growth Restriction)

May 2018

PROTOCOL TITLE 'The Dutch STRIDER (Sildenafil TheRapy In Dismal prognosis Early-onset intrauterine growth Restriction) Trial'

**Protocol ID**	**ZonMW 80-83600-98-20081**
**Short title**	**Dutch-STRIDER**
**EudraCT number**	**2012-004112-63**
**Version**	**8.2.0**
**Date**	**May 2018**
**Coordinating investigator/project leader**	**W Ganzevoort**
**Principal investigator(s)**	**W Ganzevoort**
AMC Amsterdam	**W Ganzevoort**
MMC Veldhoven	**J van Laar**
LUMC Leiden	**M Sueters**
Isala Klinieken Zwolle	**J van Eyck**
VUMC Amsterdam	**MA de Boer**
MUMC Maastricht	**S Al-Nasiry**
UMCG Groningen	**A Elvan-Taspinar**
Radboud MC Nijmegen	**J Van Drongelen**
Erasmus MC Rotterdam	**JJ Duvekot**
UMCU Utrecht	**JB Derks**
Inselspital Bern	**L Raio**
**Sponsor**	**AMC**
**Subsidising party**	***ZonMW***
**Independent expert (s)**	**WM Ankum**
**Laboratory sites**	***Local laboratory sites***
**Pharmacy**	***AMC pharmacy***<br>***Meibergdreef 9***<br>***1105 AZ Amsterdam***<br>***Phone: 0031 2056 62445***

PROTOCOL SIGNATURE SHEET

Name	Signature	Date
**Head of Department:**<br><br>***J van der Post***		
**Coordinating Investigator/Project leader/Principal Investigator:**<br><br>***W Ganzevoort***		

TABLE OF CONTENTS

1. INTRODUCTION AND RATIONALE 10
2. OBJECTIVES 12
3. STUDY DESIGN 13
4. STUDY POPULATION 14
   4.1 Population (base) 14
   4.2 Inclusion criteria 14
   4.3 Exclusion criteria 14
   4.4 Sample size calculation 14
5. TREATMENT OF SUBJECTS 16
   5.1 Investigational product/treatment 16
   5.2 Use of co-intervention (if applicable) 16
   5.3 Escape medication (if applicable) 16
6. INVESTIGATIONAL PRODUCT 18
   6.1 Name and description of investigational product(s) 18
   6.2 Summary of findings from non-clinical studies 18
   6.3 Summary of findings from clinical studies 19
   6.4 Summary of known and potential risks and benefits 19
   6.5 Description and justification of route of administration and dosage 20
   6.6 Dosages, dosage modifications and method of administration 20
   6.7 Preparation and labelling of Investigational Medicinal Product 20
   6.8 Drug accountability 20
7. NON-INVESTIGATIONAL PRODUCT 22
8. METHODS 23
   8.1 Study parameters/endpoints 23
      8.1.1 Main study parameter/endpoint 23
      8.1.2 Secondary study parameters/endpoints (if applicable) 23
      8.1.3 Other study parameters (if applicable) 23
   8.2 Randomisation, blinding and treatment allocation 23
   8.3 Study procedures 23
   8.4 Withdrawal of individual subjects 26
      8.4.1 Specific criteria for withdrawal (if applicable) 27
   8.5 Replacement of individual subjects after withdrawal 27
   8.6 Follow-up of subjects withdrawn from treatment 27
   8.7 Premature termination of the study 28
9. SAFETY REPORTING 28
   9.1 Section 10 WMO event 28
   9.2 AEs, SAEs and SUSARs 28
      9.2.1 Adverse events (AEs) 28
      9.2.2 Serious adverse events (SAEs) 28
      9.2.3 Context specific SAE reporting 29
9.2.4. Suspected unexpected serious adverse reactions (SUSARs) 30

9.3 Annual safety report 31
9.4 Follow-up of adverse events 31
9.5 Data Safety Monitoring Board (DSMB) / Safety Committee 31
10. STATISTICAL ANALYSIS 33
10.1 Primary study parameter(s) 33
10.2 Secondary study parameter(s) 34
10.3 Other study parameters 34
10.4 Interim analysis (if applicable) 35
11. ETHICAL CONSIDERATIONS 36
11.1 Regulation statement 36
11.2 Recruitment and consent 36
11.3 Objection by minors or incapacitated subjects (if applicable) 36
11.4 Benefits and risks assessment, group relatedness 36
11.5 Compensation for injury 36
11.6 Incentives (if applicable) 37
12. ADMINISTRATIVE ASPECTS, MONITORING AND PUBLICATION 38
12.1 Handling and storage of data and documents 38
12.2 Monitoring and Quality Assurance 38
12.3 Amendments 38
12.4 Annual progress report 38
12.5 End of study report 39
12.6 Public disclosure and publication policy 39
13. STRUCTURED RISK ANALYSIS 40
13.1 Potential issues of concern 40
13.2 Synthesis 44
14. Ancillary studies 45
14.1 Questionnaires Influence of Sildenafil on sexuality 45
14.2 Evaluation of cardiac function 45
14.3 Neonatal renal oxygensaturation 50
14.4 Analysis of the placenta 53
14.5 Pharmacokinetics/ pharmacodynamics 54
15. REFERENCES 56

LIST OF ABBREVIATIONS AND RELEVANT DEFINITIONS

ABR	ABR form, General Assessment and Registration form, is the application form that is required for submission to the accredited Ethics Committee (In Dutch, ABR = Algemene Beoordeling en Registratie)
AC	Abdominal Circumference
ACE	Angiotensin Converting Enzyme
AE	Adverse Event
BP	Blood Pressure
BPD	BronchoPulmonary Dysplasia
BSID	Bayley Scales of Infant Development
CCMO	Central Committee on Research Involving Human Subjects; in Dutch: Centrale Commissie Mensgebonden Onderzoek
CBCL	Child Behaviour CheckList
CNS	Central Nervous System
CP	Cerebral Palsy
CTG	Cardiotocography
DSMB	Data Safety Monitoring Board
EDF	End Diastolic Flow
EFW	Estimate of Fetal Weight
EudraCT	European drug regulatory affairs Clinical Trials
FGR	Fetal Growth Restriction
GCP	Good Clinical Practice
GMFCS	Gross Motor Function Classification Scale -questionnaire
GMP	Good Manufacturing Practice
HELLP	Hemolysis Elevated Liver enzymes Low Platelets
IC	Informed Consent
ICH	IntraCerebral Hemorrhage
IMP	Investigational Medicinal Product
IMPD	Investigational Medicinal Product Dossier
LMP	Last Menstrual Period
MDI	Mental Developmental Index
METC	Medical research ethics committee (MREC); in Dutch: medisch ethische toetsing commissie (METC)
MgSO4	Magnesium Sulphate
MRI	Magnetic Resonance Imaging
NEC	Necrotising EnteroColitis
NICU	Neonatal Intensive Care Unit
PDI	Psychomotor Developmental Index
PIGF	Placental Growth Factor
PVL	PeriVentricular Leukomalacia
RCT	Randomised Controlled Trial
RN	Research Nurse

ROP	Retinopathy Of Prematurity
(S)AE	(Serious) Adverse Event
SPC	Summary of Product Characteristics (in Dutch: officiële productinfomatie IB1-tekst)
Sponsor	The sponsor is the party that commissions the organisation or performance of the research, for example a pharmaceutical company, academic hospital, scientific organisation or investigator. A party that provides funding for a study but does not commission it is not regarded as the sponsor, but referred to as a subsidising party.
STV	Short-Term Variation
SUSAR	Suspected Unexpected Serious Adverse Reaction
Wbp	Personal Data Protection Act (in Dutch: Wet Bescherming Persoonsgevens)
WMO	Medical Research Involving Human Subjects Act (in Dutch: Wet Medisch-wetenschappelijk Onderzoek met Mensen

SUMMARY

Rationale: Severe, early-onset fetal growth restriction (FGR) due to placental insufficiency is associated with a high risk of perinatal morbidity with long-lasting sequelae and mortality. Placental insufficiency is the result of abnormal formation and function of the placenta (placentation) with inadequate remodelling of the maternal spiral (uteroplacental) arteries. There is currently no therapy available with demonstrated effectiveness. Evidence suggests Sildenafil citrate improves uteroplacental blood flow, growth, and meaningful outcomes.

Objective: To evaluate the effectiveness of sildenafil (versus placebo) in achieving healthy perinatal survival.

Study design: Multicenter nationwide randomized placebo-controlled clinical trial.

Study population: Women with a singleton pregnancy between 20 and 30 weeks with severe fetal growth restriction of likely placental origin, and with estimated significant likelihood of perinatal death.

Intervention: Sildenafil 25mg or placebo tablet orally three times daily.

Main study parameters/endpoints: Perinatal healthy survival, i.e. survival without severe neonatal morbidity at term age.

Nature and extent of the burden and risks associated with participation, benefit and group relatedness: Taking tablets three times daily. No additional ultrasounds, other than standard clinical protocol, one extra blood sample at inclusion. No risks anticipated, unexpected medication-associated risks can’t be excluded on beforehand.

1. INTRODUCTION AND RATIONALE

An estimated 700 to 900 cases of severe early-onset fetal growth restriction are managed yearly in the academic institutions. This patient group consumes disproportionate amounts of care and has a high likelihood of premature delivery, both for fetal and for secondary maternal indications such as the development of the maternal syndrome of pre-eclampsia. As these FGR children are born very preterm, they carry significant risks of neonatal mortality, major and minor morbidity, and long-term health sequelae. These are not only strongly gestational-age related, but also related to FGR. The effect of severe early- onset FGR is particularly significant: less than a third of these fetuses will survive their uniform neonatal intensive care unit (NICU) stay (if they are born alive) without significant neurodevelopmental sequelae. Survival rates for severely growth-restricted fetuses very remote from term (<28 weeks' gestation) vary between 7-33%.

Currently, there are no specific evidence-based therapies for early-onset severe fetal growth restriction. In the absence of therapeutic interventions, standard management consists of intensive monitoring and counselling. Non-specific interventions include primarily lifestyle modifications such as reducing or stopping work, stopping aerobic exercise, rest at home, and hospital admission for rest and surveillance. These interventions, which are not supported by sound evidence of efficacy, are used in the belief that rest will reduce the steal from the uteroplacental circulation to the glutei and quadriceps muscles. The other intervention is that of increased fetal and maternal surveillance in the period of fetal viablity, so that decisions around management and timing of delivery can be made. The role of fetal surveillance by ultrasound and cardiotocography is clear; the key facet of maternal surveillance is to monitor the secondary development of the dangerous maternal syndrome of pre-eclampsia that will ultimately complicate approximately 40% of these pregnancies. Effective amelioration of the placental insufficiency would add tremendously to the therapeutic arsenal.

Doppler waveform analysis of pregnancies complicated by FGR suggests compromised uteroplacental circulation and placental hypoperfusion. There is ample evidence from mouse models of growth restriction that sildenafil increases average pup birth weight, including improving intermediate parameters of uteroplacental blood flow (umbilical artery, uterine artery). Sildenafil citrate may thus offer a potential therapeutic strategy to improve uteroplacental blood flow in human pregnancies complicated by severe fetal growth restriction of placental origin. There is evidence to support the effect of sildenafil in humans. A small trial in women with early pre-eclampsia (the maternal expression of the state of placental insufficiency) showed no effect on pre-eclampsia, but a strong effect on birth weight. Also, evidence from a cohort study by collaborators in Vancouver showed a tendency towards more liveborn children that survived intact to primary discharge after sildenafil therapy. From all observations thus far there are no indications that sildenafil in the second trimester has significant fetotoxicity.

On the basis of preliminary research, centers are already adopting the treatment with sildenafil. There is however significant uncertainty regarding true health benefits. Moreover, potential harm is not yet excluded. Fetotoxicity is unlikely, since this has not been described

yet, but theoretically possible. For this reason evidence from randomised trials is needed before widespread implementation.

.

2. OBJECTIVES

The Dutch STRIDER (Sildenafil TheRapy In Dismal prognosis Early-onset fetal growth Restriction) Trial is a double-blind randomised controlled trial (RCT) designed to evaluate whether in pregnant women with severe early-onset FGR of placental origin, Sildenafil citrate compared with placebo increases the likelihood of intact perinatal survival until term age. This is defined by survival to term age without evidence of either severe central nervous system (CNS) injury (by ultrasound and/or magnetic resonance imaging [MRI]) or non-CNS severe morbidity (bronchopulmonary dysplasia, retinopathy of prematurity, or necrotising enterocolitis requiring surgery).

The secondary objectives of study are

1) to evaluate whether Sildenafil citrate, compared to placebo, increases the likelihood of improved fetal growth velocity assessed by ultrasound abdominal circumference measurements (AC);

2) to evaluate whether Sildenafil citrate, compared to placebo, increases the likelihood of age-adequate performance on the two-year Bayley scales of infant development (BSID)-III (composite cognitive score and composite motor score);

3) to assess co-incidence and severity of the maternal syndrome of pre-eclampsia / HELLP-syndrome

4) to evaluate fetal and placental dopplers at study entrance and development of Doppler parameters during treatment

5) to evaluate the role of the placenta(bed) in early severe FGR and the influence of Sildenafil

Announced subgroup analyses:

to evaluate the effect of Sildenafil on the abovementioned outcome measures, in subgroups defined by a) an abnormal or normal serum level of placental growth factor (PlGF); b) other patient characteristics available at baseline such as gestational age, estimated fetal weight.

3. STUDY DESIGN

Double-blind randomized placebo-controlled trial, in all ten Dutch perinatal tertiary care centers, during 2014-2017.

The ten Dutch perinatal tertiary care centers are:

- Academisch Medisch Centrum, Amsterdam
- Maxima Medisch Centrum, Veldhoven
- Leids Universitair Medisch Centrum, Leiden
- Isala Klinieken, Zwolle
- VU Medisch Centrum, Amsterdam
- Maastricht Universitair Medisch Centrum, Maastricht
- Universitair Medisch Centrum Groningen, Groningen
- Radboud Medisch Centrum, Nijmegen
- Erasmus Medisch Centrum, Rotterdam
- Universitair Medisch Centrum Utrecht, Utrecht

4. STUDY POPULATION

4.1 Population (base)

Dutch pregnant women referred to tertiary care referral centers for evaluation and management of severe early-onset fetal growth restriction

4.2 Inclusion criteria

Inclusion criteria ((I OR II) AND III):

I. at 20^0-27^6 weeks: an ultrasound measurement of the fetal abdominal circumference (AC) <3rd percentile for gestational age or an ultrasound estimate of fetal weight (EFW) <5th percentile

OR

II. at 28^0-29^6 weeks: an ultrasound estimate of fetal weight (EFW) <700 grams using Hadlock C formula

AND

III. likely placental origin defined by (a AND/OR b AND/OR c AND/OR d)
   a. the presence of uterine artery notching
   b. abnormal flow velocity patterns of the umbilical artery or middle cerebral artery
   c. maternal hypertensive disorders
   d. low PlGF in point-of-care assessment

4.3 Exclusion criteria

I. Plan to terminate pregnancy for maternal or fetal indication within days
II. Known multiple pregnancy
III. Identified congenital anomalies or congenital infection
IV. Maternal age at eligibility <18 years
V. Cocaine use
VI. Current use of sildenafil
VII Current use of cyp3A5 inhibitors: amiodaron, azitromycine, ciclosporine, claritromycine, diltiazem, erytromycine, fluconazol, itraconazol, ketoconazol, verapamil, voriconazol.
VIII Recent myocardial infarction or stroke

4.4 Sample size calculation

Assumptions for power calculations are made on the basis of clinical relevance and a pilot cohort[1]. In the pilot cohort a 29% rate of intact survival until discharge (placebo-treated) vs 50% (Sildenafil-treated) was seen. We have decided for a difference of 15% to be clinically relevant. A 29% rate of intact survival until discharge (placebo-treated) vs 44% (Sildenafil-treated), with an α of 0.05, we will have 80% power to detect this difference if we randomise 161 women per group. Therefore, accepting a 10% drop-out rate, the total sample size will be 354 women. We have a history of very low rates of loss to follow-up in our RCTs. Also, we shall minimise loss to follow-up by having the site study co-ordinator establish a close relationship with the study participants, through regular study contacts (at the planned weekly

antenatal assessments (minimum), during hospitalisation(s), and at delivery, as applicable) and the availability to answer any questions about the Trial.

The 10 tertiary care centers in the Dutch Consortium for women's health and reproductivity studies each see an approximate 3-4 patients with severe fetal growth restriction of likely placental origin every month. With an expected uptake of the trial in 50% of patients (missed inclusions from failure to seek informed consent, denial of informed consent) each center may be able to recruit 20 patients per year. With some centers expected not to recruit optimally, a sum total of 120 patients per year is realistic. Enrolment of participants will be over 3-year duration. The clinical outcomes for non-consenting women will be tracked to evaluate the external validity of the STRIDER Trial findings.

5 TREATMENT OF SUBJECTS

5.1 Investigational product/treatment

Sildenafil 25 mg or look-alike placebo tablets three times daily orally from randomization until delivery or 32 weeks gestation.

5.2 Use of co-intervention

Advice for rest, ultrasound fetal monitoring, delivery (cesarean section) for fetal indication if fetus deemed viable, corticosteroids for fetal lung maturation.

5.3Escape medication

Not applicable.

5.4 Other treatments

Indications for delivery:

These criteria apply to all patients if any of the following conditions are met irrespective of the management based on randomised group (sildenafil or placebo):

- CTG criteria: If an elevated pulsatility index exists, patients should have an Doppler ultrasound every 2 weeks. If the Doppler ultrasound shows absent or reversed EDF women should be admitted to hospital, corticosteroids should be administered and a CTG twice a day. STV (short-term variation) should be calculated daily from the CTG. The cut off ‘rescue’ value of STV for delivering based on CTG at 26+0 to <29+0 weeks is if STV <3,5 msec; and 29+0 to <32+0 weeks if STV < 4 msec. Or if, irrespective of STV, there are spontaneous repeated persistent unprovoked decelerations on CTG.
- Umbilical artery Doppler criteria: Absolute indications for delivery in all randomised arms:
    - >32+0 weeks deliver if reversed umbilical artery EDF.
    - >34+0 weeks deliver if absent umbilical artery EDF.
- Delivery may be undertaken according to local policies after 30+0 weeks if there is reversed umbilical artery EDF, and after 32+0 weeks if there is absent umbilical EDF.
- Ductus venosus measurements can be used per local protocol for timing of delivery.
- Maternal indication may also be an indication for delivery.

Limits of fetal viability:

In this study, performed by the 10 Perinatal Intensive Care Centers we consider an estimated fetal weight of ≥600 gram at ≥27 weeks of gestation the standard limits of fetal viability. In the period before either limit, individualized limits may be adapted, down to an estimated fetal weight of ≥500 gram at ≥26 weeks of gestation. This implicates a policy where clinicians abstain from delivery if fetuses are below this limit and the perceived chance of intact perinatal survival is very small.

Doppler ultrasound is repeated at least every two weeks. Women with a fetus that is deemed viable according to gestational age and weight criteria are admitted for intensive fetal monitoring if Doppler ultrasound shows absent or reversed EDF women. Cardiotocography will be performed according to local protocol and will be used for timing of delivery. Criteria for intervention are:

- Spontaneous repeated persistent unprovoked decelerations on CTG
- Diminished short-term variation (STV) preferably calculated with computerized CTG (some decreased variation may be accepted in the period 24-72 hours after first corticosteroid administration). The cut off ‘rescue’ value of STV for delivering based on CTG at 26+0 to <29+0 weeks is if STV <3,5 msec; and 29+0 to <32+0 weeks if STV < 4 msec.
- Umbilical artery Doppler criteria: Absolute indications for delivery in all randomised arms:
  - >32+0 weeks deliver if reversed umbilical artery EDF.
  - >34+0 weeks deliver if absent umbilical artery EDF.

- Ductus venosus measurements can be used per local protocol for timing of delivery.
- Maternal indication may also be an indication for delivery.

6. INVESTIGATIONAL PRODUCT

6.1.Name and description of investigational product(s)

Sildenafil 25 mg (as citrate) tablets and placebo tablets for oral administration will be produced by Tiofarma according to GMP. The placebo tablets will have identical appearance as the sildenafil tablets More detailed description about the IMP will be provided in the IMPD.

6.2 Summary of findings from non-clinical studies

There was one systematic review of the literature in 2007.[2] This review described studies evaluating the effects of sildenafil on uterine vessels or myometrium either in vitro or in experimental animal models, as well as clinical trials and case reports on the outcome of pregnant women treated with sildenafil. The information was obtained from: three in vitro studies, five studies performed in experimental animal models, four studies on women with fertility and sterility disorders receiving 100 mg/day of sildenafil intravaginally, and two case reports of pregnant women who received sildenafil for the treatment of pulmonary hypertension. Incubation with sildenafil of different in vitro preparations resulted in vasodilator and uterine relaxant effects. No evidence of teratogenicity was observed in the studies performed in mice, rats and dogs. Sildenafil increased fetal weight in rats. In women, contradictory results on uterine blood flow and endometrial development were reported after the intravaginal administration of sildenafil. No adverse fetal outcomes were reported in the two pregnant women with pulmonary hypertension receiving sildenafil late in their pregnancy.

After 2007, 21 more studies have been published that were identified in the present search.
There were 11 experimental animal model studies. One study showed 1.5 times more body weight gain in the offspring of guinea pigs exposed to sildenafil during gestation, and favored fetal tolerability to induced intrapartum asphyxia.[3] In another study of healthy Sprague-Dawley rats sildenafil had no effect on pup number and size.[4]This was also not the case in a study in L-NAME treated Sprague-Dawley rats.[5] A fourth study in healthy pregnant Wistar rats showed increased fetal size, but no decreased survival rates.[6] In a fifth study, a pre-eclampsia model of L-NAME treated Sprague-Dawley rats, sildenafil reduced the plasma levels of anti-angiogenic factors involved in the maternal syndrome of placental insufficiency.[7] The next study demonstrated improved endothelial function in L-NAME treated Wistar rats, a rat pre-eclampsia model.[8] In another study, sildenafil citrate treatment dose-dependently increased fetal weight in an ovine model.[9] In contrast, in a single umbilical artery ligation sheep model of growth restriction, there was no positive effect of sildenafil.[10] In a Wistar pregnant rat pre-eclampsia model sildenafil improved uterine artery blood flow and fetal weight gain and increased survival rates without fetotoxic effects, and reversed maternal hypertension and proteinuria.[11] Moreover, the offspring from these sildenafil-treated rat mothers had normalised learning ability.[12] Finally, in a mouse model of pre-eclampsia (a catecholamine knock-out model) abnormal fetal weight normalized and abnormal umbilical Doppler waveforms improved after sildenafil treatment.[13]

There were 4 new human in vitro studies of vasoactivity. One older study (already in the 2007 review) showed increased myometrial small artery vasoconstriction and decreased endothelium-dependent vasodilatation in small vessels taken at delivery from women with versus without fetal growth restriction, which was significantly antagonized by sildenafil administration.[14] Two other similar studies in women with pre-eclampsia versus controls failed to demonstrate this effect.[15, 16] In another study, it was shown that sildenafil evoked relaxation responses in the umbilical arteries from neonates born from preeclamptic mothers that were almost similar to the responses in umbilical arteries from a normal pregnancy.[17] A similar study in arteries dissected from placentas of normal pregnancies, sildenafil citrate produced dose dependent vasodilatation.[18]

For more detailed information about the registered product, see attachment SPC Viagra.

6.3 Summary of findings from clinical studies

There were two studies of pharmacokinetics and safety profiles. One study showed rapid clearance of the drug in neonates having been administered sildenafil for persistant pulmonary hypertension, but a longer half-life in neonates compared to adult controls.[19] There were no increased ocular complications from sildenafil administration.[20]
There are two clinical studies, one randomized clinical trial (RCT) in preeclampsia, and one cohort study in fetal growth restriction. The RCT was a small study of sildenafil citrate (doses 20-40-80 mg three times daily) versus placebo in 35 women with preeclampsia at gestational ages 24-34 weeks. The drug was well tolerated with no increase in maternal or fetal adverse outcomes, but without beneficial effects in this population.[16]The cohort study was in women with severe fetal growth restriction below 25 weeks or 600 grams and dismal prognosis. Women were offered sildenafil 25mg three times daily until delivery. Those who were actually treated (n=10) were compared with controls (n=17). Fetal estimated growth improved significantly more often in treated patients, and there was a non-significant trend towards improvement in intact survival: 50% versus 29% (study underpowered for this outcome).[1] Finally, there was one case report showing resolution of high uterine artery pulsatility index and notching following sildenafil citrate treatment in a growth-restricted 26 week pregnancy, that went on to delivery at 33 weeks.[21]
For more detailed information about the registered product, see attachment SPC Viagra.

6.4 Summary of known and potential risks and benefits

For more detailed information about the registered product, see attachment SPC Viagra. Known side effects of Sildenafil are: headache, blushing, dyspepsia, visual disturbances, stuffy nose, dizziness and disturbances in color vision.

There are major cardiovascular events associated with the use of sildenafil. There is also a recent suggestion of an association of sildenafil use with melanoma development. [22] All major events are very unlikely to be of any significance in this patient group and the perinatal risks of the pathological process under study far outweigh the theoretical risks. Of course, patients in the study will be closely monitored for these outcomes.

6.5 Description and justification of route of administration and dosage

For more detailed information about the registered product, see attachment SPC Viagra.

Our understanding is that there is no evidence of using Sildenafil in pregnancy and therefore no experience in term of dose range but Sildenafil doses already used for pulmonary hypertension in neonates and young children are much smaller than 25mg (25-500micrograms per kg of body weight).

The choice for the dosage of 25 mg three times a day is based on the existing research on sildenafil in IUGR. Von Dadelszen et al [1] used 25 mg three times daily as intervention and showed increased AC growth compared with sildenafil naïve patients. Samangaya et al [16] concluded that Sildenafil in the escalating dose regimen 20–80 mg three times daily was well tolerated, with no increase in maternal or fetal morbidity or mortality but did not prolong pregnancy duration in women with preeclampsia.

6.6 Dosages, dosage modifications and method of administration

For more detailed information about the registered product, see attachment SPC Viagra.

6.7 Preparation and labelling of Investigational Medicinal Product

Sildenafil and placebo tablets will be produced by Tiofarma B.V. Information about the composition and production will be provided in the Investigational Medicinal Product Dossier (IMPD). The bottles containing 90 tablets sildenafil (as citrate) or placebo will be produced and labeled as IMP in compliance with GMP and other applicable regulatory requirements. The sponsor will arrange delivery of sildenafil and placebo to the trial sites. No investigational medicinal product will be shipped until the sponsor has verified that all regulatory required documents and approvals for the site are available.

The investigational medicinal product should be stored according to the information provided on the label and in such a manner that accidental loss or destruction or access by an unauthorized person is prevented.

6.8 Drug accountability

The investigator, or a pharmacist or other appropriate individual who is designated by the investigator, should maintain records of the product's delivery to the trial site, the inventory at the site, the use by each patient, and the return to the sponsor or alternative disposition of unused product(s). These records should include dates, quantities, lot numbers, expiration dates and patient study identification (if applicable). Investigators should maintain records that document adequately that the patients were provided the doses specified by the protocol and reconcile all investigational product(s) received from the sponsor

Partially used investigational medicinal product should not be redispensed to either the same or another patient after it has been returned.

The trial site should destroy used or partially used study drug containers after drug accountability records have been completed. Destruction should be documented.

7. NON-INVESTIGATIONAL PRODUCT

N/A

8. METHODS

8.1 Study parameters/endpoints

8.1.1. Main study parameter/endpoint

Intact neonatal survival until term age

8.1.2. Secondary study parameters/endpoints (if applicable)

1) to evaluate whether Sildenafil citrate, compared to placebo, increases the likelihood of improved fetal growth velocity assessed by ultrasound abdominal circumference measurements (AC);
2) to evaluate whether Sildenafil citrate, compared to placebo, increases the likelihood of age-adequate performance on the two-year Bayley scales of infant development (BSID)-III (composite cognitive score and composite motor score);
3) to assess co-incidence and severity of the maternal syndrome of pre-eclampsia / HELLP-syndrome

8.1.3. Other study parameters (if applicable)

Fetal Doppler studies, PlGF point-of-care assessment

8.2 Randomisation, blinding and treatment allocation

Randomisation will be centrally controlled using an on-line computerised randomisation service. Centres will be able to access the randomisation service 24hr/day. Masking to the next allocated treatment will be achieved by central randomisation and random block sizes of 2 to 6. The trial will be double-blinded and placebo-controlled. Subjects will be randomized in a 1:1 ratio to study medication (Sildenafil citrate) and placebo. Randomisation will be stratified by centre (to prevent any imbalance between groups in aspects of maternal or neonatal care that may differ between centres). All personnel, except for the pharmacist, and the patient will remain blinded to the treatment being received. In case of SUSAR's the randomisation code could be broken prematurely, for cessation of the intervention drug (sildenafil or placebo) will be adequate.

8.3 Study procedures

The intervention will be oral Sildenafil citrate 25mg tid. Study medication (Viagra® or indistinguishable placebo tablets, produced and supplied by Tiofarma) will be taken 3 times daily from randomization until delivery (maximum 32 weeks).

MANAGEMENT

Co-interventions will be documented: fetal surveillance, lifestyle modification, betamethasone for fetal lung maturation (once gestational age and/or EFW deemed appropriate by treating clinicians), detection of superimposed pre-eclampsia, $MgSO_4$ for perinatal neuroprotection, and delivery (indication, timing, and route). The STRIDER Trial protocol allows centres to provide their usual 'real-world' care.

All women with severe growth restriction (also those who do not participate) will receive increased fetal and maternal surveillance according to local protocols, consisting of fetal ultrasound surveillance at randomisation, 2 days, 5 days, 10 days and 14 days after recruitment; and subsequently every 12-16 days below viability limits; every 6-8 days beyond viability limits. Ultrasound surveillance will include umbilical artery, middle cerebral artery, ductus venosus and aortic isthmus Doppler indices; fetal biometry; amniotic fluid index; deepest vertical amniotic fluid pocket. Fetal surveillance for current fetal condition will be with computerised CTG (depending on unit practice; only at and beyond the limits of viability), timed depending on ultrasound findings and admission status.

Standard maternal surveillance will include, at least, the measurement of blood pressure, proteinuria (dipstick and random protein:creatinine ratio), pulse oximetry, complete blood count, creatinine, uric acid, aspartate transaminase, bilirubin, and albumin. In addition experimental biochemical markers of placental function will be taken alongside this trial and stored. Tests will be repeated at least every 12-16 days in outpatients and every 6-8 days in inpatients.

BP management decisions should not be made based on home BP measurements alone. Clinicians should treat hypertension with labetalol, nifedipine or methyldopa, as reflected in national and international guidelines. The following must not be used: atenolol (which may impair intrauterine fetal growth), or ACE inhibitors, angiotensin receptor antagonists, or direct renin inhibitors (which are or are presumed to be fetotoxic). Sildenafil, itself an effective antihypertensive agent in pregnancy, is not a contraindication for other antihypertensive use, but management should be adjusted according to observed effects.

There is no standard of care for many obstetric interventions. As such, the STRIDER Trial protocol allows centres to provide their usual form of care. This may involve differential monitoring which will reflect the way the treatments would be applied in normal clinical practice. However, randomisation is stratified by center and data are collected on potential co-interventions (e.g., bed rest, hospitalisation, and antihypertensives [other than Sildenafil]) and their impact on outcome will be examined by secondary analyses. No special restrictions will apply with regards to diet, activities or other lifestyle items, unless so indicated by the treating physician.

During data collection, data management teams, investigators, site personnel and subjects will remain blinded to whether women received the study medication or placebo throughout the entire study period.
For the primary outcome a team of observers will jointly review the charts for consensus on the presence or absence of the outcomes. Severe central nervous system (CNS) injury (diagnosed by ultrasound and/or magnetic resonance imaging [MRI]) is defined by periventricular leukomalacia (PVL) grade II or more or IntraCerebral Hemorrhage (ICH) grade III or more or Hydrocephalus. Non-CNS severe morbidity is defined by

bronchopulmonary dysplasia (BPD; the requirement of ambulatory oxygen therapy > 36 weeks corrected gestational age), retinopathy of prematurity (ROP requiring treatment such as laser therapy; grade 2/3 or more), or necrotising enterocolitis (NEC; requiring surgery). This evaluation will also include focus on the detection of unexpected fetoxicity from sildenafil.

For the secondary hypothesis - that Sildenafil citrate therapy increases the likelihood of increased growth velocity (measured by abdominal circumference [ultrasound]) for fetuses of pregnancies complicated by severe early-onset FGR -, fetal growth velocity will be defined as the average daily increase in ultrasound-estimated AC. The AC measured pre-randomisation will be compared with the most recent informative measurement of the AC at least 12 days previously. AC measurements will be compared with the gestational age [in days since last menstrual period (LMP)] at which that measurement lies most closely to the 50th percentile. Therefore, should the interval between scans be 18 days, and AC have grown by the equivalent of 9 days, then the average daily growth will be 0.5.

For the specific secondary hypothesis - that Sildenafil citrate therapy increases the likelihood of age-adequate performance (both MDI [Mental Developmental Index] and PDI [Psychomotor Developmental Index] >85) on the two-year Bayley scales of infant development (BSID)-III - children will be seen and tested at two years by trained paediatric psychologists. This will also be tested with the Bayley scores of both groups as continuous measures, to test if a clinically significant difference is present (>7 points difference between groups, i.e. ½ standard deviation). The study will have sufficient power for these outcomes. Also, neurological development will be tested for CP (cerebral palsy) rate including severity scaling with Gross Motor Function Classification Scale (GMFCS) and a CBCL-questionnaire (Child Behaviour CheckList) will be administered. Difference in incidence of head circumference <-1 SDS will also be tested.

There will be planned subgroup analyses for differential effects to evaluate the effect of Sildenafil on the abovementioned outcome measures in subgroups defined by a) an abnormal or normal serum level of placental growth factor (PlGF); b) other patient characteristics available at baseline such as gestational age, estimated fetal weight;

It is the intention (outside the scope of this proposal subject to secondary evaluation by the ethics committee) to perform several additional studies in this complex patient group. Firstly, paediatric follow-up according to the Dutch National protocol at five years (including IQ, CBCL, movement-ABC, and pediatric check-up). Secondly, pathophysiological MRI-studies regarding the development of white matter damage in children, and the association between volumetric studies at 36-40 weeks corrected gestational age and long-term outcomes. Both studies will also include focus on the detection of unlikely fetoxicity from sildenafil. Thirdly, fundamental research on biomedical diagnostic tools protein, RNA-markers, epigenetic changes, for example longitudinal PlGF protein levels, or plasma RNA-markers (CSH1, GH1, ADAM12). These are promising

biochemical markers of placental insufficiency (among which PlGF) that will be tested alongside this trial. Analysis will be performed after closure of the trial for prediction on fetal outcome, placental and fetal growth.

Placenta(bed) investigations will be performed. In many cases a caesarean section will be the mode of delivery with the opportunity to take placentabed biopsies (uterine wall including vessels) after delivery of the placenta. Approximately 50% of these biopsies will contain spiral arteries (ref Veerbeek) in which sildenafil causes vasodilatation through the enhancement of the NO pathway (ref Wareing). Furthermore biopsies of basal plate and parenchyma of the placenta will be taken and processed for further analysis, such as methylation studies, immunologic cell subset analysis and vascular markers. After these fresh biopsies placenta will processed to be presented to the pathologist for standard investigations after FGR. Results of the placenta investigations will be related to outcome and follow up.

8.4 Withdrawal of individual subjects

Subjects may withdraw at any time or be withdrawn by the investigator if the woman violates the study plan or for administrative and /or safety reasons. Patients who withdraw from the study will remain in their treatment group for the intent-to-treat analysis. Every effort will be made to obtain complete information on each patient randomized. The only reason for not obtaining complete information is that the patient was lost to follow up or that she withdraws consent to access her medical chart after delivery. Once a woman has been randomized, even though she stops taking the study medication for any reason, follow-up will be continued including the planned visits, maternal and fetal surveillance. If a woman refuses to complete her follow-up visits with the research nurse (RN), the RN will confirm permission to consult her hospital chart in order to be able to complete information on the primary outcome of the study. In this case, her data will be considered in the final analysis. If a woman stops the medication because of nausea, she will be encouraged to resume taking the medication as soon as she feels the nausea has subsided.

8.4.1. Specific criteria for withdrawal (if applicable)

Not applicable.

8.5 Replacement of individual subjects after withdrawal

Not applicable.

8.6 Follow-up of subjects withdrawn from treatment

If a woman refuses to complete her follow-up visits with the research nurse (RN), the RN will confirm permission to consult her hospital chart in order to be able to complete information on the primary outcome of the study. In this case, her data will be considered in the final analysis.

8.7 Premature termination of the study

The DSMB can decide to terminate the study prematurely in case of safety concerns. The DSMB can decide to terminate the study prematurely in case the interim analysis shows clear benefit or harm of either one of the treatments (sildenafil or placebo) or due to external evidence. One interim analysis will be performed after 177 cases, with $p < .01$.

9. SAFETY REPORTING

9.1 Section 10 WMO event

In accordance to section 10, subsection 4, of the WMO, the sponsor will suspend the study if there is sufficient ground that continuation of the study will jeopardise subject health or safety. The sponsor will notify the accredited METC without undue delay of a temporary halt including the reason for such an action. The study will be suspended pending further review by the accredited METC. The investigator will take care that all subjects are kept informed.

9.2 AEs, SAEs and SUSARs

9.2.1. Adverse events (AEs)

Adverse events are defined as any undesirable experience occurring to a subject during the study, whether or not considered related to sildenafil. All adverse events reported spontaneously by the subject or observed by the investigator or his staff will be recorded.

9.2.2 Serious adverse events (SAEs)

A serious adverse event is any untoward medical occurrence or effect that at any dose:

- results in death;
- is life threatening (at the time of the event);
- requires hospitalisation or prolongation of existing inpatients' hospitalisation;
- results in persistent or significant disability or incapacity;
- is a congenital anomaly or birth defect;
- Any other important medical event that may not result in death, be life threatening, or require hospitalization, may be considered a serious adverse experience when, based upon appropriate medical judgement, the event may jeopardize the subject or may require an intervention to prevent one of the outcomes listed above.

Whether or not related to the study medication, any SAE which occurs to patients from the time the consent is signed through 30 days after completion of the study must be reported within 24 hours to one of the individual(s) listed on the Contact Information Sheet, the Principal Investigator of the study. The DSMB should be informed after each 3 SAEs.

The sponsor will report the SAEs through the web portal *ToetsingOnline* to the accredited METC that approved the protocol, within 15 days after the sponsor has first knowledge of the serious adverse reactions. Context specific SAEs (specified in paragraph 9.2.3) will not be reported for each individual case through the web portal *ToetsingOnline*. Context specific SAEs will be reported to the DSMB by line listing after completion of the study by each 50 participants. Context specific SAEs will be reported to the METC by line listing yearly.

Non context-specific SAEs that result in death or are life threatening should be reported expedited through the web portal *ToetsingOnline*. The expedited reporting will occur not later than 7 days after the responsible investigator has first knowledge of the adverse reaction. This is for a preliminary report with another 8 days for completion of the report.

9.2.3 Context-specific SAE reporting

This study population (possibly critically ill preterm infants) has a high risk of serious complications (so-called “context-specific SAE’s”), which are inherent to their vulnerable condition and unrelated to the intervention which is under evaluation in this trial.
These complications are included in the primary and secondary outcomes of this study and are recorded in the Case Report Form. This documentation will include the classification/gradation of the complication, type of action taken if appropriate (with some complications a wait and see approach is warranted).
In light of the above, immediate and individual reporting of all these condition related complications will not enhance the safety of study. Therefore we will report only maternal SAEs and fetal or neonatal death immediately through toetsingonline. The DSMB should be informed after each 3 non context-specific SAEs, including fetal or neonatal death. The neonatal context-specific SAEs will be reported at on beforehand determined evaluation moments of DSMB, after completion of the study by each 50 participants.
The context-specific SAEs that will be identified include the following events:

- Maternal hospital admission for observation and delivery
- Maternal hypertensive disorder
- NICU admission
- RDS requiring surfactant/BPD
- Persistent pulmonary hypertension of the newborn requiring inhalation NO.
- Mechanical ventilation, requiring postnatal steroids
- Supplemental oxygen
- Hypo- or hypertension
- Hyperglycaemia requiring the use of insulin therapy
- Nosocomial infection, like sepsis, meningitis and pneumonia
- Pulmonary hemorrhage, pneumothorax and pulmonary interstitial emphysema
- Hemodynamic significant patent ductus arteriosus for which medical intervention or surgical ligation is needed
- Necrotising enterocolitis (NEC), diagnosed at surgery, autopsy or by radiographic finding of pneumotosis intestinalis or hepatobiliary gas (Bell stage II)
- Gastrointestinal bleeding
- Isolated gastrointestinal perforation diagnosed on abdominal radiography
- Intraventricular haemorrhage (IVH) and/or periventricular leucomalacia (PVL), including grading on cerebral ultrasonography according to protocol defined by Ment et.al. [23]

- Retinopathy of prematurity, including grading following international classification

9.2.4 Suspected unexpected serious adverse reactions (SUSARs)

Adverse reactions are all untoward and unintended responses to an investigational product related to any dose administered.

Unexpected adverse reactions are SUSARs if the following three conditions are met:

1. the event must be serious (see chapter 9.2.2);
2. there must be a certain degree of probability that the event is a harmful and an undesirable reaction to the medicinal product under investigation, regardless of the administered dose;
3. the adverse reaction must be unexpected, that is to say, the nature and severity of the adverse reaction are not in agreement with the product information as recorded in:
   - Summary of Product Characteristics (SPC) for an authorised medicinal product;
   - Investigator's Brochure for an unauthorised medicinal product.

The sponsor will report expedited the following SUSARs through the web portal *ToetsingOnline* to the METC:

- SUSARs that have arisen in the clinical trial that was assessed by the METC;
- SUSARs that have arisen in other clinical trials of the same sponsor and with the same medicinal product, and that could have consequences for the safety of the subjects involved in the clinical trial that was assessed by the METC.

The remaining SUSARs are recorded in an overview list (line-listing) that will be submitted once every half year to the METC. This line-listing provides an overview of all SUSARs from the study medicine, accompanied by a brief report highlighting the main points of concern.

The expedited reporting of SUSARs through the web portal ToetsingOnline is sufficient as notification to the competent authority.

The sponsor will report expedited all SUSARs to the competent authorities in other Member States, according to the requirements of the Member States.

The expedited reporting will occur not later than 15 days after the sponsor has first knowledge of the adverse reactions. For fatal or life threatening cases the term will be maximal 7 days for a preliminary report with another 8 days for completion of the report.

Whether or not related to the study medication, any SUSAR which occurs to patients from the time the consent is signed through 30 days after completion of the study must be reported within 24 hours to one of the individual(s) listed on the Contact

Information Sheet, the Principal Investigator of the study and then the DSMB should be immediately informed. All subjects with SUSAR must be followed-up for outcome by the DSMB.

9.3 Annual safety report

In addition to the expedited reporting of SUSARs, the sponsor will submit, once a year throughout the clinical trial, a safety report to the accredited METC, competent authority, and competent authorities of the concerned Member States.
This safety report consists of:

- a list of all suspected (unexpected or expected) serious adverse reactions, along with an aggregated summary table of all reported serious adverse reactions, ordered by organ system, per study;
- a report concerning the safety of the subjects, consisting of a complete safety analysis and an evaluation of the balance between the efficacy and the harmfulness of the medicine under investigation.

9.4 Follow-up of adverse events

All AEs will be followed until they have abated, or until a stable situation has been reached. Depending on the event, follow up may require additional tests or medical procedures as indicated, and/or referral to the general physician or a medical specialist.
SAEs need to be reported till end of study within the Netherlands, as defined in the protocol

9.5 Data Safety Monitoring Board (DSMB) / Safety Committee

Data Safety Monitoring Board (DSMB) that will be called upon to monitor compliance and cross-over, and review any unexpected adverse events. The DSMB will include prof. dr. J.G.P. Tijssen, MD, PhD - statistician (chair), prof. dr. F.M. Helmerhorst, MD, PhD – gynaecologist LUMC, prof. dr. M.P.M. Burger, MD, PhD – gynaecologist AMC, dr. J.H. van der Lee, MD, PhD – epidemiologist AMC, dr D.P. van der Ham – gynaecologist Martini Ziekenhuis and dr. T.R. de Haan, MD, PhD – neonatologist AMC.
The DSMB will meet as required, by teleconference, to review any unexpected adverse events. The DSMB has the right to review any variables that may have an impact on the trial.

One interim analysis on safety parameters (neonatal mortality and morbidity) will be performed for the first 177 cases, with a $p < .01$. Interim analysis will be done after 177 deliveries.

The advice(s) of the DSMB will only be sent to the sponsor of the study. Should the sponsor decide not to fully implement the advice of the DSMB, the sponsor will send the

advice to the reviewing METC, including a note to substantiate why (part of) the advice of the DSMB will not be followed.

10. STATISTICAL ANALYSIS

The University of British Columbia (UBC) will host data management. Through the UBC Perinatal Clinical Trials Unit data are collected. All data will be analysed on an intention-to-treat basis: that is, for the purpose of analysis, all women will be included in the group to which they have been randomised. If women are lost to follow-up, they will be included in their randomised group for all outcomes for which we have information. Descriptive statistics will be calculated to check for major dissimilarities between study groups with respect to baseline information. Sensitivity analyses (including imputation strategies) will be performed to estimate the potential effects of missing data.

Statistical analysis will be with Chi-square tests (two-sided) and non-parametric Mann–Whitney tests, where appropriate, using the latest version of SPSS (SPSS Chicago, Illinois, USA). The null hypothesis assumes equivalence in the primary outcome. Subgroup analysis for the effect of low PlGF at inclusion, abnormal fetal Dopplers at inclusion, and for the effect of other baseline parameters (gestational age, estimated fetal weight), and for participating centre on healthy survival will be performed. One interim analysis on safety parameters (neonatal mortality and morbidity) will be performed for the first 177 cases, with a $p < .01$. Interim analysis will be done after 177 deliveries.

CONSORTIUM

The logistics of the consortium studies, including this study, are taken care of by cluster coordinators and research nurses/midwives. Each cluster of medical centers in The Netherlands is linked to a perinatological center, of which the cluster coordinator is responsible for the logistics, data gathering, and data completion within that cluster. Research nurses/midwives inform all participating professionals about the ongoing studies within their medical center, are the contact persons for the participating patients and professionals, and perform the data entry of study data. The trial bureau of the consortium is responsible for the approval of the studies within the consortium at the medical ethical commissions of the participating hospitals.

OUTCOME ASSESSMENT

During data collection, data management teams, investigators, site personnel and subjects will remain blinded to whether women received the study medication or placebo throughout the entire study period.

10.1 Primary study parameter(s)

For the primary outcome a team of observers will jointly review the charts for consensus on the presence or absence of the outcomes. Severe central nervous system (CNS) injury (diagnosed by ultrasound and/or magnetic resonance imaging [MRI]) is defined by periventricular leukomalacia (PVL) grade II or more or IntraCerebral Hemorrhage (ICH) grade III or more or Hydrocephalus. Non-CNS severe morbidity is defined by bronchopulmonary dysplasia (BPD; the requirement of ambulatory oxygen therapy > 36 weeks corrected gestational age), retinopathy of prematurity (ROP requiring treatment

such as laser therapy; grade 2/3 or more), or necrotising enterocolitis (NEC; requiring surgery). This evaluation will also include focus on the detection of unexpected fetoxicity from sildenafil.

10.2 Secondary study parameter(s)

For the secondary hypothesis - that Sildenafil citrate therapy increases the likelihood of increased growth velocity (measured by abdominal circumference [ultrasound]) for fetuses of pregnancies complicated by severe early-onset FGR -, fetal growth velocity will be defined as the average daily increase in ultrasound-estimated AC. The AC measured pre-randomisation will be compared with the most recent informative measurement of the AC at least 12 days previously. AC measurements will be compared with the gestational age [in days since last menstrual period (LMP)] at which that measurement lies most closely to the 50th percentile. Therefore, should the interval between scans be 18 days, and AC have grown by the equivalent of 9 days, then the average daily growth will be 0.5.

For the specific secondary hypothesis - that Sildenafil citrate therapy increases the likelihood of age-adequate performance (both MDI [Mental Developmental Index] and PDI [Psychomotor Developmental Index] >85) on the two-year Bayley scales of infant development (BSID)-III - children will be seen and tested at two years by trained paediatric psychologists. This will also be tested with the Bayley scores of both groups as continuous measures, to test if a clinically significant difference is present (>7 points difference between groups, i.e. ½ standard deviation). The study will have sufficient power for these outcomes. Also, neurological development will be tested for CP (cerebral palsy) rate including severity scaling with Gross Motor Function Classification Scale (GMFCS) and a CBCL-questionnaire (Child Behaviour CheckList) will be administered. Difference in incidence of head circumference <-1 SDS will also be tested.

10.3 Other study parameters

There will be planned subgroup analyses for differential effects to evaluate the effect of Sildenafil on the abovementioned outcome measures in subgroups defined by a) an abnormal or normal serum level of placental growth factor (PlGF); b) other patient characteristics available at baseline such as gestational age, estimated fetal weight.

It is the intention (outside the scope of this proposal) to perform several additional studies in this complex patient group. Firstly, paediatric follow-up according to the Dutch National protocol at five years (including IQ, CBCL, movement-ABC, and pediatric check-up). Secondly, pathophysiological MRI-studies regarding the development of white matter damage in children, and the association between volumetric studies at 36-40 weeks corrected gestational age and long-term outcomes. Both studies will also include focus on the detection of unlikely fetoxicity from sildenafil. Thirdly, fundamental research on biomedical diagnostic tools protein, RNA-markers, epigenetic changes, for example

longitudinal PIGF protein levels, or plasma RNA-markers (CSH1, GH1, ADAM12). These are promising biochemical markers of placental insufficiency (among which PIGF) that will be tested alongside this trial. Analysis will be performed after closure of the trial for prediction on fetal outcome, placental and fetal growth

10.4 Interim analysis (if applicable)

One interim analysis on safety parameters (neonatal mortality and morbidity) will be performed for the first 177 cases, with a $p < .01$. Interim analysis will be done after 177 deliveries.

11. ETHICAL CONSIDERATIONS

11.1 Regulation statement

The study will be conducted according to the principles of the Declaration of Helsinki (see for the most recent version: www.wma.net) and in accordance with the Medical Research Involving Human Subjects Act (WMO) and other guidelines, regulations and Acts.

11.2 Recruitment and consent

Women are referred to the 10 perinatal tertiary care centers, as part of routine care. After establishing eligibility, women and their partners will be counselled by the supervising doctor or by the venter-appointed research midwife/nurse. They will also receive written information (see appendices). They will have ample time (1-7 days) to consider their decision.

11.3 Objection by minors or incapacitated subjects (if applicable)

Not applicable.

11.4 Benefits and risks assessment, group relatedness

The expected/potential benefit for participants is the hypothesis that sildenafil improves the intact neonatal survival until term age and increases the likelihood of increased growth velocity (measured by abdominal circumference [ultrasound]) for fetuses.
Participants could experience side effects of the study medication. Known side effects of Sildenafil are described in section 12.1. There are no other specific risks for participants.

11.5 Compensation for injury

The sponsor/investigator has a liability insurance which is in accordance with article 7, subsection 6 of the WMO.

The sponsor (also) has an insurance which is in accordance with the legal requirements in the Netherlands (Article 7 WMO and the Measure regarding Compulsory Insurance for Clinical Research in Humans of 23th June 2003). This insurance provides cover for damage to research subjects through injury or death caused by the study.

1. € 450.000,-- (i.e. four hundred and fifty thousand Euro) for death or injury for each subject who participates in the Research;
2. € 3.500.000,-- (i.e. three million five hundred thousand Euro) for death or injury for all subjects who participate in the Research;
3. € 5.000.000,-- (i.e. five million Euro) for the total damage incurred by the organisation for all damage disclosed by scientific research for the Sponsor as ‘verrichter’ in the meaning of said Act in each year of insurance coverage.

The insurance applies to the damage that becomes apparent during the study or within 4 years after the end of the study.

11.6 Incentives (if applicable)

Not applicable.

12. ADMINISTRATIVE ASPECTS, MONITORING AND PUBLICATION

12.1 Handling and storage of data and documents

The patients will receive a unique code, based on the order they have been included in the study. This code will be anonymous and not be based on name or date of birth of the patient.
The CRF will be electronical and the persons who will have access to the data are: the study coordinator, the study investigator and the study nurse.

12.2 Monitoring and Quality Assurance

Monitoring will be performed in compliance with Good Clinical Practice (GCP) and other rules and regulations in order to achieve high quality research and secure patient safety. An independent monitor from the NVOG Consortium will have access to the data and source documents of the trial. Based on the Site Specific Monitoring program of the NVOG Consortium, site evaluation visits will be performed to review the quality of the participating sites, as was recently audited and endorsed by the UMCU Medical Ethics Committee.

12.3 Amendments

A ‘substantial amendment’ is defined as an amendment to the terms of the METC application, or to the protocol or any other supporting documentation, that is likely to affect to a significant degree:

- the safety or physical or mental integrity of the subjects of the trial;
- the scientific value of the trial;
- the conduct or management of the trial; or
- the quality or safety of any intervention used in the trial.

All substantial amendments will be notified to the METC and to the competent authority.

Non-substantial amendments will not be notified to the accredited METC and the competent authority, but will be recorded and filed by the sponsor.

12.4 Annual progress report

The sponsor/investigator will submit a summary of the progress of the trial to the accredited METC once a year. Information will be provided on the date of inclusion of the first subject, numbers of subjects included and numbers of subjects that have completed the trial, serious adverse events/ serious adverse reactions, other problems, and amendments.

12.5 End of study report

The sponsor will notify the accredited METC and the competent authority of the end of the study within a period of 90 days. The end of the study is defined as the last patient's last visit.

In case the study is ended prematurely, the sponsor will notify the accredited METC and the competent authority within 15 days, including the reasons for the premature termination.

Within one year after the end of the study, the investigator/sponsor will submit a final study report with the results of the study, including any publications/abstracts of the study, to the accredited METC and the Competent Authority.

12.6 Public disclosure and publication policy

After completing the trial and data analysis, the results of the trial will be published as soon as possible in an international journal on obstetrics. We aim publishing within 1 year after completing the trial.

13. STRUCTURED RISK ANALYSIS

13.1 Potential issues of concern

For product information, see the attached Summary of Product Characteristics.

a. Level of knowledge about mechanism of action
There is ample in vitro knowledge of the mechanism of action. This has been evidenced by in vitro mechanistic studies and in vivo pathophysiological studies. The in vivo studies have been both in animal models as well as human effect models. The specific test for the mechanism of action in the pregnant uterus with normal and abnormal uteroplacental circulation is limited and the reason for this study. The results of the field of sildenafil-research however can be extrapolated to this setting.

The mechanism of action of sildenafil is known for the indication of erectile dysfunction:

Sildenafil is an oral therapy for erectile dysfunction. In the natural setting, i.e. with sexual stimulation, it restores impaired erectile function by increasing blood flow to the penis. The physiological mechanism responsible for erection of the penis involves the release of nitric oxide (NO) in the corpus cavernosum during sexual stimulation. Nitric oxide then activates the enzyme guanylate cyclase, which results in increased levels of cyclic guanosine monophosphate (cGMP), producing smooth muscle relaxation in the corpus cavernosum and allowing inflow of blood. Sildenafil is a potent and selective inhibitor of cGMP specific phosphodiesterase type 5 (PDE5) in the corpus cavernosum, where PDE5 is responsible for degradation of cGMP. Sildenafil has a peripheral site of action on erections. Sildenafil has no direct relaxant effect on isolated human corpus cavernosum but potently enhances the relaxant effect of NO on this tissue. When the NO/cGMP pathway is activated, as occurs with sexual stimulation, inhibition of PDE5 by sildenafil results in increased corpus cavernosum levels of cGMP. Therefore sexual stimulation is required in order for sildenafil to produce its intended beneficial pharmacological effects.

Studies *in vitro* have shown that sildenafil is selective for PDE5, which is involved in the erection process. Its effect is more potent on PDE5 than on other known phosphodiesterases. There is a 10-fold selectivity over PDE6 which is involved in the phototransduction pathway in the retina. At maximum recommended doses, there is an 80-fold selectivity over PDE1, and over 700-fold over PDE2, 3, 4, 7, 8, 9, 10 and 11. In particular, sildenafil has greater than 4,000-fold selectivity for PDE5 over PDE3, the cAMP-specific phosphodiesterase isoform involved in the control of cardiac contractility.

b. Previous exposure of human beings with the test product(s) and/or products with a similar biological mechanism
There are numerous reports of the use of the drug sildenafil for different indications. There are sufficient reports of exposure of pregnant women and their fetuses to sildenafil, being given for another indication, without knowledgeable side-effects. The information of use of sildenafil in this population is scarce and the reason for this study

c. Can the primary or secondary mechanism be induced in animals and/or in *ex-vivo* human cell material?
No.

d. Selectivity of the mechanism to target tissue in animals and/or human beings

The mechanism of action is well-defined. Since the process that sildenafil influences is universal in the human system (smooth muscle relaxation) the drug is not selective for a specific organ but it is fairly selective for smooth muscle relaxation.

e. Analysis of potential effect

Randomized controlled trial.

f. Pharmacokinetic considerations

Absorption

Sildenafil is rapidly absorbed. Maximum observed plasma concentrations are reached within 30 to 120 minutes (median 60 minutes) of oral dosing in the fasted state. The mean absolute oral bioavailability is 41% (range 25-63%). After oral dosing of sildenafil AUC and Cmax increase in proportion with dose over the recommended dose range (25-100 mg). When sildenafil is taken with food, the rate of absorption is reduced with a mean delay in tmax of 60 minutes and a mean reduction in Cmax of 29%.

Distribution

The mean steady state volume of distribution (Vd) for sildenafil is 105 l, indicating distribution into the tissues. After a single oral dose of 100 mg, the mean maximum total plasma concentration of sildenafil is approximately 440 ng/mL (CV 40%). Since sildenafil (and its major circulating
N-desmethyl metabolite) is 96% bound to plasma proteins, this results in the mean maximum free plasma concentration for sildenafil of 18 ng/mL (38 nM). Protein binding is independent of total drug concentrations.
In healthy volunteers receiving sildenafil (100 mg single dose), less than 0.0002% (average 188 ng) of the administered dose was present in ejaculate 90 minutes after dosing.

Biotransformation

Sildenafil is cleared predominantly by the CYP3A4 (major route) and CYP2C9 (minor route) hepatic microsomal isoenzymes. The major circulating metabolite results from N-demethylation of sildenafil.
This metabolite has a phosphodiesterase selectivity profile similar to sildenafil and an *in vitro* potency for PDE5 approximately 50% that of the parent drug. Plasma concentrations of this metabolite are approximately 40% of those seen for sildenafil. The N-desmethyl metabolite is further metabolised, with a terminal half-life of approximately 4 h.

Elimination

The total body clearance of sildenafil is 41 L/h with a resultant terminal phase half-life of 3-5 h. After either oral or intravenous administration, sildenafil is excreted as metabolites predominantly in the faeces (approximately 80% of administered oral dose) and to a lesser extent in the urine (approximately 13% of administered oral dose).

Pharmacokinetics in special patient groups

Elderly

Healthy, elderly volunteers (65 years or over) had a reduced clearance of sildenafil, resulting in approximately 90% higher plasma concentrations of sildenafil and the active N-desmethyl metabolite compared to those seen in healthy younger volunteers (18-45 years). Due to age-differences in plasma protein binding, the corresponding increase in free sildenafil plasma concentration was approximately 40%.

Renal insufficiency

In volunteers with mild to moderate renal impairment (creatinine clearance = 30-80 mL/min), the pharmacokinetics of sildenafil were not altered after receiving a 50 mg single oral dose. The mean AUC and Cmax of the N-desmethyl metabolite increased 126% and 73% respectively, compared to age-matched volunteers with no renal impairment. However, due to high inter-subject variability, these differences were not statistically significant. In volunteers with severe renal impairment (creatinine clearance <30 mL/min), sildenafil clearance was reduced, resulting in mean increases in AUC and Cmax of 100% and 88% respectively compared to age-matched volunteers with no renal impairment. In addition, N-desmethyl metabolite AUC and Cmax values were significantly increased 79% and 200% respectively.

Hepatic insufficiency
In volunteers with mild to moderate hepatic cirrhosis (Child-Pugh A and B) sildenafil clearance was reduced, resulting in increases in AUC (84%) and Cmax (47%) compared to age-matched volunteers with no hepatic impairment. The pharmacokinetics of sildenafil in patients with severely impaired hepatic function have not been studied.

g. Study population
Women with a singleton pregnancy between 20 and 30 weeks with severe fetal growth restriction of likely placental origin, and with estimated significant likelihood of perinatal death.

h. Interaction with other products
- Effects of other medicinal products on sildenafil

In vitro studies
Sildenafil metabolism is principally mediated by the cytochrome P450 (CYP) isoforms 3A4 (major route) and 2C9 (minor route). Therefore, inhibitors of these isoenzymes may reduce sildenafil clearance and inducers of these isoenzymes may increase sildenafil clearance.

In vivo studies
Population pharmacokinetic analysis of clinical trial data indicated a reduction in sildenafil clearance when co-administered with CYP3A4 inhibitors (such as ketoconazole, erythromycin, cimetidine).
Although no increased incidence of adverse events was observed in these patients, when sildenafil is administered concomitantly with CYP3A4 inhibitors, a starting dose of 25 mg should be considered.
Co-administration of the HIV protease inhibitor ritonavir, which is a highly potent P450 inhibitor, at steady state (500 mg twice daily) with sildenafil (100 mg single dose) resulted in a 300% (4-fold) increase in sildenafil Cmax and a 1,000% (11-fold) increase in sildenafil plasma AUC. At 24 hours, the plasma levels of sildenafil were still approximately 200 ng/mL, compared to approximately 5 ng/ml when sildenafil was administered alone. This is consistent with ritonavir's marked effects on a broad range of P450 substrates. Sildenafil had no effect on ritonavir pharmacokinetics. Based on these pharmacokinetic results co-administration of sildenafil with ritonavir is not advised (see section 4.4) and in any event the maximum dose of sildenafil should under no circumstances exceed 25 mg within 48 hours.

Co-administration of the HIV protease inhibitor saquinavir, a CYP3A4 inhibitor, at steady state (1200 mg three times a day) with sildenafil (100 mg single dose) resulted in a 140% increase in sildenafil Cmax and a 210% increase in sildenafil AUC. Sildenafil had no effect on saquinavir pharmacokinetics (see section 4.2). Stronger CYP3A4 inhibitors such as ketoconazole and itraconazole would be expected to have greater effects.

When a single 100 mg dose of sildenafil was administered with erythromycin, a moderate CYP3A4 inhibitor, at steady state (500 mg twice daily. for 5 days), there was a 182% increase in sildenafil systemic exposure (AUC). In normal healthy male volunteers, there was no evidence of an effect of azithromycin (500 mg daily for 3 days) on the AUC, Cmax, tmax, elimination rate constant, or subsequent half-life of sildenafil or its principal circulating metabolite. Cimetidine (800 mg), a cytochrome P450 inhibitor and non-specific CYP3A4 inhibitor, caused a 56% increase in plasma sildenafil concentrations when co-administered with sildenafil (50 mg) to healthy volunteers.

Grapefruit juice is a weak inhibitor of CYP3A4 gut wall metabolism and may give rise to modest increases in plasma levels of sildenafil.
Single doses of antacid (magnesium hydroxide/aluminium hydroxide) did not affect the bioavailability of sildenafil.
Although specific interaction studies were not conducted for all medicinal products, population pharmacokinetic analysis showed no effect of concomitant treatment on sildenafil pharmacokinetics when grouped as CYP2C9 inhibitors (such as tolbutamide, warfarin, phenytoin), CYP2D6 inhibitors (such as selective serotonin reuptake inhibitors, tricyclic antidepressants), thiazide and related diuretics, loop and potassium sparing diuretics, angiotensin converting enzyme inhibitors, calcium channel blockers, beta-adrenoreceptor antagonists or inducers of CYP450 metabolism (such as rifampicin, barbiturates). In a study of healthy male volunteers, co-administration of the endothelin antagonist, bosentan, (an inducer of CYP3A4 [moderate], CYP2C9 and possibly of CYP2C19) at steady state (125 mg twice a day) with sildenafil at steady state (80 mg three times a day) resulted in 62.6% and 55.4% decrease in sildenafil AUC and Cmax, respectively. Therefore, concomitant administration of strong CYP3A4 inducers, such as rifampin, is expected to cause greater decreases in plasma concentrations of sildenafil.
Nicorandil is a hybrid of potassium channel activator and nitrate. Due to the nitrate component it has the potential to result in a serious interaction with sildenafil.

- Effects of sildenafil on other medicinal products

In vitro studies
Sildenafil is a weak inhibitor of the cytochrome P450 isoforms 1A2, 2C9, 2C19, 2D6, 2E1 and 3A4 (IC50 > 150 µM). Given sildenafil peak plasma concentrations of approximately 1 µM after recommended doses, it is unlikely that VIAGRA will alter the clearance of substrates of these isoenzymes.
There are no data on the interaction of sildenafil and non-specific phosphodiesterase inhibitors such as theophylline or dipyridamole.

In vivo studies
Consistent with its known effects on the nitric oxide/cGMP pathway (see section 5.1), sildenafil was shown to potentiate the hypotensive effects of nitrates, and its co-administration with nitric oxide donors or nitrates in any form is therefore contraindicated (see section 4.3 of SPC).
Concomitant administration of sildenafil to patients taking alpha-blocker therapy may lead to symptomatic hypotension in a few susceptible individuals. This is most likely to occur within 4 hours post sildenafil dosing (see sections 4.2 and 4.4). In three specific drug-drug interaction studies, the alpha-blocker doxazosin (4 mg and 8 mg) and sildenafil (25 mg, 50 mg, or 100 mg) were administered simultaneously to patients with benign prostatic hyperplasia (BPH) stabilized on doxazosin therapy. In these study populations, mean additional reductions of supine blood pressure of 7/7 mmHg, 9/5 mmHg, and 8/4 mmHg, and mean additional reductions of standing blood pressure of 6/6 mmHg, 11/4 mmHg, and 4/5 mmHg, respectively, were observed. When sildenafil and doxazosin were administered simultaneously to patients stabilized on doxazosin therapy, there were

infrequent reports of patients who experienced symptomatic postural hypotension. These reports included dizziness and light-headedness, but not syncope.
No significant interactions were shown when sildenafil (50 mg) was co-administered with tolbutamide (250 mg) or warfarin (40 mg), both of which are metabolised by CYP2C9. Sildenafil (50 mg) did not potentiate the increase in bleeding time caused by acetyl salicylic acid (150 mg).

Sildenafil (50 mg) did not potentiate the hypotensive effects of alcohol in healthy volunteers with mean maximum blood alcohol levels of 80 mg/dl.
Pooling of the following classes of antihypertensive medication: diuretics, beta-blockers, ACE inhibitors, angiotensin II antagonists, antihypertensive medicinal products (vasodilator and centrally-acting), adrenergic neurone blockers, calcium channel blockers and alpha-adrenoceptor blockers, showed no difference in the side effect profile in patients taking sildenafil compared to placebo treatment. In a specific interaction study, where sildenafil (100 mg) was co-administered with amlodipine in hypertensive patients, there was an additional reduction on supine systolic blood pressure of 8 mmHg. The corresponding additional reduction in supine diastolic blood pressure was 7 mmHg. These additional blood pressure reductions were of a similar magnitude to those seen when sildenafil was administered alone to healthy volunteers (see section 5.1).
Sildenafil (100 mg) did not affect the steady state pharmacokinetics of the HIV protease inhibitors, saquinavir and ritonavir, both of which are CYP3A4 substrates.
In healthy male volunteers, sildenafil at steady state (80 mg t.i.d.) resulted in a 49.8% increase in bosentan AUC and a 42% increase in bosentan Cmax (125 mg b.i.d.).

i. Predictability of effect

j. Can effects be managed?

k. Effects on the fetus

Little research has been done to assess the effect of sildenafil on the fetus, while used during pregnancy. The existing literature states that, based on observational studies [24] and experimental animal studies [25] sildenafil does not appear to increase the risk of congenital malformations in exposed pregnancies and no embryo-fetal harm has been observed. Two recent case reports support this statement. [21, 26]
Based on the existing literature we do not expect more severe adverse events than one would expect based on the natural course of the clinical situation and prognosis of this patient population.

13.2 Synthesis

There is ample in vitro knowledge of the mechanism of action of sildenafil. This has been evidenced by in vitro mechanistic studies and in vivo pathophysiological studies. The in vivo studies have been both in animal models as well as human effect models. The specific test for the mechanism of action in the pregnant uterus with normal and abnormal uteroplacental circulation is limited and the reason for this study. The results of the field of sildenafil-research however can be extrapolated to this setting.
The risk for study participants is estimated as moderate.

14. Ancillary studies

14.1 Questionnaires: Influence of Sildenafil on sexuality

To monitor the influence of Sildenafil on sexuality of pregnant women, participants of the trial will be asked to complete a questionnaire on female sexuality: 'McCoy Female Sexuality Questionnaire' (short version). This questionnaire consisting of nine questions about experiencing sexuality will be answered three times during participation of the study: at inclusion, after four weeks and after eight weeks. If participants are not using the study medication anymore or have delivered their baby, the second and/or third questionnaire will not be offered.
The questionnaire is offered in the online program LimeSurvey, hosted by Clinical Research Unit in Academic Medical Hospital, Amsterdam. Participants of STRIDER study need to fill in a separate informed consent for the questionnaires. If a participant consents to participate in the questionnaires, she will receive a personal link to the LimeSurvey questionnaire so she can fill in the questionnaire at a time and place that is convenient for her.

14.2 Evaluation of cardiac function

Introduction
Intrauterine growth restriction (IUGR) is associated with increased risk of perinatal complications and neonatal morbidity[27]. Epidemiological data have shown an association between IUGR and cardiovascular morbidity and mortality during adulthood, including increased rates of coronary heart disease, stroke, hypertension and non-insulin-dependent diabetes[28-33].These findings support the existence of a maladaptive programming process *in utero*, whereby a stimulus or insult at a critical, sensitive period of early life has permanent effects on structure, physiology and metabolism[32,34]. In the case of IUGR the programming may result from adaptations to a condition where placental nutrient supply fails to match fetal demand. Recently compensatory feto-placental up-regulation of the nitric oxide system during fetal growth restriction (FGR) has been shown. These events are followed by nitric oxide pathway down-regulation postnatally, increasing susceptibility to cardiovascular disorders later in life[32].
Phosphodiesterase inhibition has been reported to cause placental vasodilation in pregnancies with FGR. Sildenafil citrate works by inhibiting type 5 phosphodiesterase (PDE5) which is responsible for the degradation of cGMP to guanosine monophosphate[35]. The inhibition of PDE5 delays the breakdown of cGMP and increases vasodilation[36]. During the Sildenafil Therapy In Dismal prognosis Early-onset intrauterine growth Restriction (STRIDER)[37] study mothers with IUGR fetuses will be treated with sildenafil in an attempt to improve placental perfusion and thus fetal growth. This study allows for a unique opportunity to examine the cardiac function of IUGR fetuses and neonates and the cardiac effects of sildenafil on fetal and cardiac function in these individuals.
Changes in cardiac function have been observed in both IUGR and small for gestational age (SGA) fetuses[27,29,38,39-50]. Changes in cardiac geometry have been reported in human fetuses and animal models. Cardiothoracic ratio and atrial areas were increased in the IUGR fetuses compared with control subjects. Left and right

sphericity indexes were decreased significantly in IUGR fetuses with thicker myocardial walls, when compared with control subjects[38]. The most probable pathophysiological mechanism for these changes is the increased myocardial workload *in utero*. Chronic intrauterine hypoxia results in increased placental vascular resistance, redistribution of cardiac output in favor of the left fetal heart, alterations of arterial structure (intima and medial thickening) and vascular tone regulation, and disturbances that contribute to the development of cardiac hypertrophy. The remodeling may be an adaptive response to the reduced substrate supply mediated through the modulation of specific gene expression at the myocardial level[38].
A few studies measuring cardiac function in IUGR fetuses using echocardiography have been performed. Both stroke volume (SV) and cardiac output (CO) have been shown to be increased in IUGR fetuses[38]. The use of M-mode and tissue Doppler imaging (TDI) has demonstrated a significantly reduced mitral annular-plane systolic excursion (MAPSE), tricuspid annular-plane systolic excursion (TAPSE), and S' in the IUGR fetuses compared with control subjects. Both the isovolumetric contraction time (IVCT) and ejection time (ET) were increased in the IUGR group. Diastolic function parameters also showed differences with decreased E' velocities and increased early/late trans-valvular filling velocities and E/E' ratios, isovolumetric relaxation time (IVRT), and E deceleration time in IUGR fetuses compared with control subjects[38]. The mean myocardial performance index (MPI) for the left ventricle was found to be significantly higher in IUGR fetuses with an abnormal umbilical artery (UA) Doppler compared to healthy fetuses[45]. The IUGR fetuses with the abnormal MPI also showed a significantly worse perinatal outcome and increased morbidity compared to those with a normal MPI (irrespective of the UA Doppler findings) and compared to the normal controles[45]. Another study showed that the MPI is raised in SGA fetuses before the arterial and venous Doppler abnormalities that characterize hypoxia are evident[47]. A higher left atrial pressure has been demonstrated in fetuses with IUGR, as indicated by the lower mobility of the septum primum, accompanied by higher ratios between early and late diastolic myocardial velocities on TDI. Placental dysfunction could be correlated to septal E'/A' ratios suggesting that this method could be useful to assess severity of placental dysfunction and fetal distress[48]. Fetal TDI is difficult however the use of the MAPSE and TAPSE can be useful as an alternative to evaluate fetal cardiac function[40]. In fetal echocardiography, M-mode longitudinal motion has previously been described as a feasible measurement with good reproducibility, and reference ranges have been published[41].

Changes in the cardiovascular system have also been observed in neonates and children who suffered IUGR or were SGA[52-58]. The fetal TAPSE, right sphericity index, IVRT, and cerebroplacental ratio have been shown to be strong predictors of postnatal vascular remodeling and infant hypertension[38]. Follow-up of SGA and IUGR fetuses into childhood showed that they had more globular hearts, reduced longitudinal motion, and impaired relaxation with an increase in radial function. Both groups showed increased blood pressure and carotid intima-media thickness. There was a linear tendency to worse cardiovascular results in IUGR as compared with SGA children[41]. SV, CO and left ventricular (LV) thickening were significantly increased in cases as compared with controls. Cases also showed a tendency to higher heart rate (HR) and LV ejection fraction. Longitudinal motion in systole was significantly reduced in IUGR group with a non-significant tendency to lower results in the SGA group. Both IUGR and SGA children showed signs of diastolic dysfunction,

mainly measured as an increase in E deceleration, decrease in diastolic annular velocities, and a tendency to higher IVRTs. There was a significant linear tendency to worse cardiac function in IUGR as compared with SGA children.

Aim
The purpose of this study is to:

1) document the cardiac function of IUGR fetuses enrolled in the STRIDER study – is there evidence for cardiac dysfunction?
2) document changes in cardiac function after starting sildenafil – is there a difference between before and after starting sildenafil and is there a difference between the treated and untreated fetuses?
3) document postnatal cardiac function (including blood pressure) – is there a difference between the treated and untreated fetuses?

Proposed Study:
A prospective study of human fetal cardiac function in fetuses with IUGR pre and post maternal sildenafil therapy.
Cardiac function will be evaluated using Doppler echocardiography before starting sildenafil therapy and one week thereafter, also patients receiving the placebo will undergo two echoes.
A postnatal evaluation of cardiac function (including TDI) will also take place.
Cardiac function will be determined as in the previous studies of Clur *et al*[59-61] with the addition of the measurement of the isovolumetric contraction and relaxation times (IVCT, IVRT), MAPSE and TAPSE, excursion index of the septum primum (EISP) and aortic isthmus Doppler and pulsatility index (PI). The IVCT and IVRT will be expressed in milliseconds and as percentages of the cardiac cycle. The ductus venosus (DV), MCA, UA and hepatic artery Dopplers will also be recorded.

Hypotheses:
1) Cardiac dysfunction is present in fetuses with IUGR enrolled in the STRIDER study.
2) Fetal cardiac dysfunction is improved by maternal intake of sildenafil in fetuses with IUGR.
3) The fetal cardiac function of the treated fetuses is better than those treated with placebo.
3) Neonatal cardiac function is better in those IUGR fetuses treated with sildenafil prenatally.

Participating Centres:
Academic Medical Center, Amsterdam; Leids Universitair Medisch Centrum, Leiden
A separate informed consent will be asked to participants.
Methods:
Study population:
30 fetuses included in the STRIDER study will be included. Of these fetuses at least one antepartum and one postpartum measurement should be available.
Measurements:
Fetal echocardiography will be performed according to standard recommendations[62] using the following equipment; Voluson E8 (GE Medical Systems, Milwaukee, WI, USA).

Recordings should be stored as uncompressed files and will all be reviewed by SAC who will also coordinate the data collection and database.
Examinations will be performed by a fetal medicine specialists with experience in performing a fetal echocardiogram or a pediatric cardiologist (SAC). After confirming the cardiac anatomy, Doppler flow evaluation using the four-chamber and outflow-tract views will be performed. To ensure the best repeatability and safety possible the following requirements will be strictly adhered to when making the measurements:

1) Mechanical and thermal indices will be <1 throughout;
2) A 3-mm pulse Doppler gate will be placed over the tricuspid valve (TV), mitral valve (MV) and pulmonary valve (PV) or positioned in the internal wall of the ascending aorta, close to the internal wall of the ascending aorta below the aortic valve (AoV), allowing concurrent display of the MV inflow and AoV outflow;
3) As the time intervals for the RV cannot usually be measured simultaneously, we will include measurements of TV inflow and PV outflow with similar HRs and, where possible, recorded in a sweep from the TV towards the PV;
4) Angle correction may be applied as appropriate up to a maximum of 15 degrees over the valves;
5) Sweep speed of 5 cm/s will be used, allowing for at least 4 consecutive heart beat registrations per recording;
6) The Wall Motion Filter (WMF) will be set on 300 Hz[37-65];
7) Gain will be adjusted to an intermediate level so as to allow for the best visualization of thin closing valve clicks without loss of the Doppler flow images;
8) The measurements will be made at the peaks of the clicks and not at the bases;
9) The 2D measurements of the semilunar valves (mm) will be measured in systole and of the atrio-venrticular (AV)-valves in diastole (i.e. with the valves open), from hinge point to hinge point, and with the use of magnification.
10) Sphericity Index: Ventricular base-to-apex lengths and transverse diameters will be measured on 2-dimentional images from an apical 4-chamber view at end-diastole. Ventricular sphericity indexes will be calculated as base-to-apex length/transverse diameter of the LV and RV, respectively[66,67];
11) MAPSE and TAPSE: will be measured by M-mode in real-time using a 2–6-MHz linear curved-array transducer in an apical or basal four-chamber view, by placing the cursor at right angles to the AV junction, marked by the valve rings; mitral (medical) and tricuspid (lateral), respectively. The maximum amplitude of motion will be taken as the extent of displacement between end-systole and end-diastole (measured in mm). For both measurements insonation by the ultrasound beam will be kept at an angle of <30 degrees to the orientation of the ventricular wall or the interventricular septum, with no angle correction applied;
12) EISP will be calculated as the ratio between the maximal excursion of the septum primum into the left atrium during diastole and the maximal diastolic diameter of the left atrium. Left and right atrial areas will be delineated on 2-dimensional images from an apical or basal 4-chamber view at end-ventricular systole (maximum point of atrial distension)[48];
13) The cardiothoracic ratio was measured from a 4-chamber view, by the area method previously described(ref);
14) The cerebroplacental ratio will be calculated by division of MCA and UA PI.

15) Ventricular end-diastolic septal and free wall thicknesses were measured by M-mode from a transverse 4-chamber view[68].

The following recordings will be made and stored for later analysis:

1) Doppler over the TV and MV in 4-chamber view.
2) Doppler recordings of MV and AoV in 5-chamber view.
3) PV Doppler in the RV outflow-tract view.
4) M-mode from a transverse 4-chamber view.
5) MAPSE and TAPSE in 4-chamber view.
6) Apical 4-chamber view at end systole (AV-valves open) and end-diastole (AV-valves closed).
7) The DV Doppler and PI.
8) The MCA Doppler and PI.
9) The UA Doppler and PI.
10) Aortic isthmus Doppler and PI.
11) The hepatic artery Doppler and PI.
12) The Ductus Venosus Doppler and PI.

Doppler flow over AV- and semilunar valves: –
The following measurements will be made on the stored recordings:-
Peak E-(early ventricular filling) and A-(atrial contraction) wave velocities (cm/s), E/A velocity ratio and time velocity integral (TVI) (cm) over the AV-valvess will be measured to assess diastolic function[69,70].
The time between AV-valve inflows (ms), semilunar valve ejection time (ms) and HR will be measured and used to calculate the MPI according to the formula a–b/b of Tei *et al.*[74], where a=time between AV-valve inflow and b=the ejection time over the semilunar valve. MPI is a measure of global cardiac function incorporating the IVCT reflecting systolic function and the IVRT reflecting early diastolic function.
The IVCT and IVRT will be measured and expressed in milliseconds and as a percentage of the cardiac cycle, e.g. IVCT 45 ms, IVRT 15ms. HR 150/min. One cardiac cycle is 400ms then IVCT is 11,25% of cardiac cycle.
The presence or absence of AV-valve regurgitation will be noted.
Systolic function will be expressed in terms of: peak velocity (m/s) and acceleration time (AT) (ms) over the semilunar valves, stroke volume (ml), cardiac output (ml/min), cardiac index (CO/kg), MAPSE/TAPSE and IVCT.
The great vessel cross-sectional area (CSA) will be calculated using the formula $CSA=\pi(diameter/2)^2$. The diameter is the annulus of the valve. Stroke volume (SV)=TVI X CSA and cardiac output (CO)=SV X HR[69,72].

2)Venous flow patterns:
DV flow patterns will be recorded by the fetal medicine specialist as previously described[74]. The sample volume will be kept as small as possible in order to avoid interferences from nearby vessels. The mean DV pulsatility index for the veins (PIV) will be calculated and evaluated according to published reference ranges[74].

3)Hepatic artery Doppler: HA flow patterns will be recorded by the fetal medicine specialist as previously described[75]. The PI and peak velocity will be recorded.

In inter-observer and intra-observer reproducibility

Special attention to keep the reproducibility as high as possible will be made as mentioned above. All measurements will be digitally stored and analyzed by one observer.

Outcome data will be collected from hospital notes/Astraia. A full postnatal echocardiogram will be performed on day 1 to 3 postapartum and day 7 to 10 postpartum. A blood pressure will also be recorded postnatally.
Outcome data on mortality and most relevant morbidity will be collected, mainly IRDS, dry lung, PPHN, chorioamnionitis, sepsis, NEC, BPD, IVH and ROP.

Statistical analysis:
Depending on the distribution of the measured values the student-t test, (normally distributed), or Mann-Whitney U test, (not normally distributed), using SPSS18[76]. A p value <0.05 will be considered statistically significant.

14.3 The effect of antenatal Sildenafil in fetal growth restriction on neonatal renal oxygen saturation

During fetal growth restriction (FGR) as a consequence of placental dysfunction, redistribution of the fetal blood circulation often occurs, favoring brain perfusion[77]. This compensatory mechanism may reverse after some time during chronic fetal hypoxia[78]. Long-term fetal hypoxia has shown in animal studies to eventually cause under vascularisation of the cerebral white matter[79]. This may account for the fact that infants born small for gestational age (SGA) shown delayed neurodevelopment growing up[80]. This intrauterine process of preferential blood flow to the brain may continue after birth[81]. Infants born after FGR have shown to have a higher cerebral tissue oxygen saturation and a lower fractional tissue oxygen extraction measured by near-infrared spectroscopy (NIRS), which may suggest increased cerebral perfusion, or decreased cerebral metabolism[82]. This phenomenon of increased cerebral oxygenation has also been seen in infants born after intrauterine exposure to maternal antihypertensive drugs, which often co-occurs with placental dysfunction and FGR[83].
Furthermore, both in SGA infants and in infants born after intrauterine exposure to antihypertensive drugs, a decreased oxygenation of the renal area has been observed, to simultaneously occur with the increased cerebral oxygenation. This has been expressed as an increased cerebro-renal oxygenation ratio (CROR)[81]. These recent findings suggest a continuous or recurrent compensational mechanism comparable to the intrauterine redistribution of blood and oxygen to the brain, also referred to as brain-sparing. Little is known about the clinical consequences of this phenomenon.

Near-infrared spectroscopy (NIRS) is a non-invasive, safe way to continuously assess tissue oxygen saturation. Tissue oxygen saturation reflects oxygen saturation of blood in three departments of the underlying tissue: arterial (estimated 25%), capillary (5%), en mainly venous blood (70%)[84]. Near-infrared light can penetrate tissue. Part of the light is then absorbed by hemoglobin (Hb) and another part with slightly different wavelength by oxygenated Hb (HbO2). Due to the different absorption specters of Hb and HbO2 for near-infrared light, the sensor detects the reflected light from two wavelengths, and the ratio of Hb and HbO2 can be calculated, which is the oxygen saturation of the underlying tissue (rSO2). By relating the fraction of tissue oxygen saturation of arterial oxygen saturation measured peripherally, the FTOE can be calculated (SpO_2- rSO_2) / SpO_2. FTOE reflects the balance between oxygen delivery and consumption. Assuming oxygen consumption in the newborn brain remains relatively stable, FTOE reflects actual perfusion state of the brain[85]. Though increasingly validated, the NIRS technique has still some uncertainties, lacking absolute precision and accuracy[86]. Also, different devices and sensors use various techniques and algorithms, hampering comparison. However, these differences have now mostly been established[87][88]. NIRS is most useful as trend monitor, but cerebral oxygen saturation values measured by NIRS of < 50% have been associated with cerebral damage[89, 90]. Furthermore, cerebral oxygen saturation values measured by NIRS have recently been shown to relate to neurodevelopmental outcome at two-three years of age, in preterm born infants[91]. NIRS for cerebral oxygenation measurements is currently increasingly being used as part of standard care for newborn infants admitted to the neonatal intensive care unit (NICU). All participating centers of this Strider sub study have already implemented cerebral oxygenation measurements using NIRS as part of standard care for newborn infants admitted to their NICU.

Besides measuring cerebral oxygen saturation using NIRS, NIRS may also be a valuable method to assess systemic organ oxygenation. A decrease in systemic organ oxygenation may be an early sign for circulatory insufficiency, for systemic oxygenation may deteriorate prior to cerebral oxygenation in case of circulatory insufficiency, due to cerebrovascular autoregulation[92, 93]. This would make the continuous assessment of systemic organ perfusion clinically useful and is a focus of current research. Abdominal oxygenation measured by NIRS is strongly correlated to central venous oxygen saturation[94] and flow in the superior mesenteric artery, measured by Doppler[95]. Also, abdominal oxygen saturation is related to venous lactate measures[96], and to the occurrence of NEC[97]. All this circumstantial evidence by lack of golden standard for organ perfusion, supports the assumption that the NIRS technique can be used to assess systemic perfusion[98].

Measurement of renal oxygen saturation by positioning the NIRS sensor in the flank region, has been validated mainly in infants with congenital heart defects. Furthermore, the CROR has previously been used as an indicator for the distribution of blood to either the brain or the lower body[81]. Finally, somatic (renal) NIRS measurements have shown to be feasible, safe and not distressing in the sick newborn infant, resulting in an increasing use in daily care[99].

Aim of this Strider sub study:
To show an effect of the administration of Sildenafil to pregnant women with severe FGR on neonatal regional cerebral (primary) and renal oxygen saturation and –extraction. Furthermore, to find an effect on the redistribution of blood after birth expressed as CROR.

Hypothesis: Sildenafil administration during FGR in pregnancy will through improvement of placental function reduce the need for brain sparing, resulting in a lower cerebral oxygen saturation, a higher renal oxygen saturation, a higher cerebral FTOE and a lower renal FTOE, end finally a lower CROR, compared to the placebo exposed infants.

Methods
After parental consent renal (and cerebral) oxygen saturation will be measured in infants admitted to the NICU according to this time schedule:
Day 1-5: 203 hours a day, between two standard moments of care, without extra disturbances for the infants, then once a week on Days 7, 14, 21, 28, 36 (+/1 2 days), or until discharge.
The sensor will be placed in the left flank (if not possible: right flank). Be means of two-sided adhesive tape suitable for the extreme sensitive skin (Mepitel®). The adhesive of the sensor will not be used in order to prevent irritation of the skin. The tip of the sensor will be placed right next to the spine. The diaper may be wrapped around the sensor. The infant is allowed to be positioned in any way suitable. Standard cerebral measurements will be performed by placing the sensor parietotemporally.
Every participating center can make use of their own available NIRS devices and sensors. We will correct for known differences in differences in hind side. One device is used per infant per measurement.
Every measurement-day during the start of the two-three hour measurement, several parameters need to be documented:

- PaCO2, Hb (during measurement, or from maximal 12 hours prior to measurement)
- blood pressure (systole/diastole/mean arterial pressure)
- type/doses catecholamines

- ventilator mode and pressures
- position (prone/supine, left/right)
- timing (start-stop), amount, and type of feeding.

Statistics

We will test for differences between mean oxygen saturation of the renal region (and cerebral region) of all available data per day, after removing artifacts. We will compare between both groups: rcSO2, rrSO2, cFTOE, rFTOE, CROR using a Student-t-test.

Sample size calculation

In order to detect a relevant difference between cerebral oxygen saturation (SD 7%) of 5% between both groups[100], with 90% power, an alpha of 5%, we will need to include 2x42 infants.

In order to have enough data every measurement day, at least the First five days after birth, we account for a dropout rate of approximately 10%. We will therefore include 92 infants from the Strider study.

Participating centers: UMCG, UMCU, Erasmus MC, Radboud UMC, VUMC, Maxima MC.

14.4 Analysis of the placenta.

Placental dysfunction is often the underlying mechanism of FGR. Placental pathology has been described in relation to both early and late FGR, usually in retrospective cohort studies [101]. Sildenafil induces vasodilatation on both sides of the placenta: in the maternal placental bed and fetal vasculature (parenchymal/chorionic vessels) with potential positive effects on growth [101-103].Effects of these specific vascular changes on placenta bed, basal plate and placental parenchyma in relation to changes in methylation patterns (maternal fetal as well as placental), immunologic changes and placental pathology need further analysis in relation to clinical outcome. Also analysis of maternal and fetal (cord) blood regarding markers of these changes need analysis.

Objectives of this sub study in the strider study:

1. To analyse the specific effect of Sildenafil on the spiral arteries (placentabed vessels) in relation to pathology the placenta.

2. To analyse the pathogenesis of severe early FGR (placebo and non-randomised group)

3. To relate the results of both groups to outcome and follow up

4. To try to come to (bio)markers of FGR

The investigations oft the placenta will comprise standard macroscopy and microspopy (immunohistochemic staining) according to local protocol. Tissue blocks will be stored (standard procedure) and presented to the pathologists of the University Medical Center Groningen or Maastricht University Medical Center for central revision of microscopy. Additional system biologic investigations such as methylation studies in both maternal vascular tissue and parenchymal tissue, immunologic cel subsets (such as M1/M2 macrophages) and vascular markers will be analysed in these two centers as well. Tissue biopsies of placenta bed, basal plate and placental parenchyma will be taken just after delivery and processed by fixing in RNA later and frozen in -80 degrees in all centers separately and stored until transport to UMCG or MUMC for analysis. Maternal and fetal (cord) blood will be processed and used for biomarkers and in applicable centers used for flowcytometry (immunologic changes, needs immediate processing)

Participating centres: all participating centres of the STRIDER study.

14.5 Pharmacokinetics/ pharmacodynamics

No sildenafil target concentration and PK/PD relationship for pre-eclampsia and fetal growth retardation have been determined yet. As a consequence it is important to analyse the plasma levels of sildenafil and metabolite desmethylsildenafil in serum of pregnant women and in the umbilical cord at delivery in the current study if delivery is during or within 72 hours after stop of study medication treatment.
Additional very small amount of blood (1.0 ml) will be taken during routine blood sampling from all pregnant woman, for example at the day of delivery or when routine blood sampling is done for hypertensive disorders. Additionally, blood from the umbilical cord will be taken after delivery. All blood samples will be collected in EDTA micro-tubes and centrifuged at each site. Plasma will be stored at -20 degrees Celsius. All samples will be analysed centrally in the pharmacy of the Erasmus MC. A LC-MSMS method for analysis of sildenafil and desmethylsildenafil is already available. For this method 100 uL plasma per sample is necessary.

14.6 Placenta

In order to acquire knowledge about the pathofysiology of severe fetal growth restriction and the effects of sildenafil on placental vascular function, a placental perfusion investigation will be performed. Understanding of the vascular morphology and comparing perfusion of the placenta of the sildenafil treated and sildenafil naive patients, might contribute to future treatment options in fetal growth restriction due to placental dysfunction.
In participating centres with the needed facilities, tissue samples from the placenta and umbilical cord will be cleaned from blood and 1) snap frozen in -80 and/or stored in RNA-later and 2) kept in formalin and embedded in formalin for histological analysis, directly after birth. Via a punch biopsy (not applicable if vaginal delivery) at the caesarian section, tissue with spiral arteries will be collected. These tissues will be used for further analyses as vascular morphology alterations (vascular remodelling, as e.g. fibrin, medial hypertrophy/hyperplasia, inflammatory infiltrates) due to sildenafil administration, with particular interest to the NO pathway, as activity of eNOS, iNOS, ROS, NO-inducers (estrogen, VEGF, shear stress (e.g. focal adhesion molecules), endothelial dysfunction and the availability of NO, ONOO and nitrotyrosine. The placenta will be directly transported to the laboratory for ex-vivo placenta perfusion if available. In this model the maternal and fetal circulation will be restored in a laboratory setting.This way we can simulate the perfusion of sildenafil and assess the vasoreactivity. We will investigate if there are functional differences in perfusion capacity between sildenafil and placebo treated women. Also, assessments will be made to understand if the duration of sildenafil treatment, gestational age and gender result in differences in perfusion capacity.

Participating centre: Erasmus MC

15. REFERENCES

1. von Dadelszen, P., et al., *Sildenafil citrate therapy for severe early-onset intrauterine growth restriction.* BJOG, 2011. **118**(5): p. 624-8.
2. Villanueva-Garcia, D., et al., *A systematic review of experimental and clinical studies of sildenafil citrate for intrauterine growth restriction and pre-term labour.* J Obstet Gynaecol, 2007. **27**(3): p. 255-9.
3. Sanchez-Aparicio, P., et al., *Effects of sildenafil on the fetal growth of guinea pigs and their ability to survive induced intrapartum asphyxia.* Am J Obstet Gynecol, 2008. **198**(1): p. 127 e1-6.
4. Sasser, J.M. and C. Baylis, *Effects of sildenafil on maternal hemodynamics and fetal growth in normal rat pregnancy.* Am J Physiol Regul Integr Comp Physiol, 2010. **298**(2): p. R433-8.
5. Nassar, A.H., et al., *Effects of sildenafil in Nomega-nitro-L-arginine methyl ester-induced intrauterine growth restriction in a rat model.* Am J Perinatol, 2012. **29**(6): p. 429-34.
6. Pellicer, B., et al., *Haemodynamic effects of long-term administration of sildenafil in normotensive pregnant and non-pregnant rats.* BJOG, 2011. **118**(5): p. 615-23.
7. Ramesar, S.V., et al., *Sildenafil citrate decreases sFlt-1 and sEng in pregnant l-NAME treated Sprague-Dawley rats.* Eur J Obstet Gynecol Reprod Biol, 2011. **157**(2): p. 136-40.
8. Turgut, N.H., et al., *The effect of sildenafil on the altered thoracic aorta smooth muscle responses in rat pre-eclampsia model.* Eur J Pharmacol, 2008. **589**(1-3): p. 180-7.
9. Satterfield, M.C., et al., *Sildenafil citrate treatment enhances amino acid availability in the conceptus and fetal growth in an ovine model of intrauterine growth restriction.* J Nutr, 2010. **140**(2): p. 251-8.
10. Miller, S.L., et al., *The effects of sildenafil citrate (Viagra) on uterine blood flow and well being in the intrauterine growth-restricted fetus.* Am J Obstet Gynecol, 2009. **200**(1): p. 102 e1-7.
11. Herraiz, S., et al., *Sildenafil citrate improves perinatal outcome in fetuses from pre-eclamptic rats.* BJOG, 2012. **119**(11): p. 1394-402.
12. Cauli, O., et al., *Treatment with sildenafil prevents impairment of learning in rats born to pre-eclamptic mothers.* Neuroscience, 2010. **171**(2): p. 506-12.
13. Stanley, J.L., et al., *Sildenafil citrate rescues fetal growth in the catechol-O-methyl transferase knockout mouse model.* Hypertension, 2012. **59**(5): p. 1021-8.
14. Wareing, M., et al., *Sildenafil citrate (Viagra) enhances vasodilatation in fetal growth restriction.* J Clin Endocrinol Metab, 2005. **90**(5): p. 2550-5.

15. Wareing, M., et al., *Phosphodiesterase-5 inhibitors and omental and placental small artery function in normal pregnancy and pre-eclampsia.* Eur J Obstet Gynecol Reprod Biol, 2006. **127**(1): p. 41-9.

16. Samangaya, R.A., et al., *A randomised, double-blinded, placebo-controlled study of the phosphodiesterase type 5 inhibitor sildenafil for the treatment of preeclampsia.* Hypertens Pregnancy, 2009. **28**(4): p. 369-82.

17. Karasu, E., et al., *Endothelial dysfunction in the human umbilical artery due to preeclampsia can be prevented by sildenafil.* Clin Exp Hypertens, 2012. **34**(2): p. 79-85.

18. Maharaj, C.H., et al., *Effects and mechanisms of action of sildenafil citrate in human chorionic arteries.* Reprod Biol Endocrinol, 2009. **7**: p. 34.

19. Mukherjee, A., et al., *Population pharmacokinetics of sildenafil in term neonates: evidence of rapid maturation of metabolic clearance in the early postnatal period.* Clin Pharmacol Ther, 2009. **85**(1): p. 56-63.

20. Kehat, R., et al., *Ocular findings of oral sildenafil use in term and near-term neonates.* J AAPOS, 2010. **14**(2): p. 159-62.

21. Lin, T.H., et al., *Resolution of high uterine artery pulsatility index and notching following sildenafil citrate treatment in a growth-restricted pregnancy.* Ultrasound Obstet Gynecol, 2012. **40**(5): p. 609-10.

22. Li, W.Q., et al., *Sildenafil Use and Increased Risk of Incident Melanoma in US Men: A Prospective Cohort Study.* JAMA Intern Med, 2014.

23. Ment, L.R., et al., *Practice parameter: neuroimaging of the neonate: report of the Quality Standards Subcommittee of the American Academy of Neurology and the Practice Committee of the Child Neurology Society.* Neurology, 2002. **58**(12): p. 1726-38.

24.Briggs, G.G.F., Roger K.; Yaffe, Sumner J., *Drugs in Pregnancy and Lactation: A Reference Guide to Fetal and Neonatal Risk, 9th Edition*. 2011, Lippincott Williams & Wilkins.

25.http://reprotox.org/Members/PrintAgentDetail.aspx?a=4086. *Agent detail - Sildenafil*.

26.Terek, D., et al., *Pulmonary arterial hypertension and pregnancy.* J Res Med Sci, 2013. **18**(1): p. 73-6.

27. Chawengsettakul S, Russameecharoen K, Wanitpongpan P. Fetal cardiac function measured by myocardial performance index of small-for-gestational age fetuses. *J Obstet Gynaaecol Res*. 2015; 41: 222-228.

28. Barker DJ. The intrauterine origins of cardiovascular and obstructive lung disease in adult life. The Marc Daniels Lecture 1990. *J R Coll Physicians Lond* 25: 129 –133, 1991.

29. Barker DJ, Osmond C, Golding J, Kuh D, Wadsworth ME. Growth in utero, blood pressure in childhood and adult life, and mortality from cardiovascular disease. *BMJ* 298: 564 –567, 1989.

30. Kaijser M, Bonamy AK, Akre O, Cnattingius S, Granath F, Norman M, Ekbom A. Perinatal risk factors for ischemic heart disease: disentangling the roles of birth weight and preterm birth. *Circulation* 117: 405– 410, 2008.
31. Curhan GC, Willett WC, Rimm EB, Spiegelman D, Ascherio AL, Stampfer MJ. Birth weight and adult hypertension, diabetes mellitus, and obesity in US men. *Circulation* 94: 3246–3250, 1996.
32. Pisaneschi S1, Boldrini A, Genazzani AR, Coceani F, Simoncini T. Feto-placental vascular dysfunction as a prenatal determinant of adult cardiovascular disease. *Intern Emerg Med.* 2013 Apr;8 Suppl 1:S41-5. doi: 10.1007/s11739-013-0925-y.
33. Ross MG, Beall MH. Adult sequelae of intrauterine growth restriction. *Semin Perinatol* 2008;32:213–8.
34. Velten M1, Gorr MW, Youtz DJ, Velten C, Rogers LK, Wold LE. Adverse perinatal environment contributes to altered cardiac development and function. *Am J Physiol Heart Circ Physiol.* 2014 May;306(9):H1334-40. doi: 10.1152/ajpheart.00056.2014. Epub 2014 Mar 7.
35. Wareing M, Myers JE, O'Hara M, Baker PN. Sildenafil citrate (Viagra) enhances vasodilatation in fetal growth restriction. J *Clin Endocrinol Metab*. 2005; 90(5): 2550-2555.).
36. Ballard SA, Gingell CJ, Tang K, Turner LA, Price ME, Naylor AM. Effects of sildenafil on the relaxation of human corpus cavernosum tissue in vitro and on the activities of cyclic nucleotide phosphodiesterase isozymes. *J Urol*. 1998; 159(6): 2164-71.)
37. Ganzevoort W, Alfirevic Z, von Dadelszen P, Kenny L, Papageorghiou A, van Wassenaer-Leemhuis A, Gluud C, Mol BW, Baker PN. STRIDER: Sildenafil Therapy In Dismal prognosis Early-onset intrauterine growth Restriction-a protocol for a systematic review with individual participant data and aggregate data meta-analysis and trial sequential analysis. *Syst Rev.* 2014 Mar 11; 3: 23. doi: 10.1186/2046-4053-3-23. Review.
38. Cruz-Lemini M, Crispi F, Valenzuela-Alcaraz B, Figueras F, Gómez O, Sitges M, Bijnens B, Gratacós E. A fetal cardiovascular score to predict infant hypertension and arterial remodeling in intrauterine growth restriction. *Am J Obstet Gynecol.* 2014 Jun;210(6):552.e1-552.e22. doi: 10.1016/j.ajog.2013.12.031. Epub 2013 Dec 22.
39. Comas M, Crispi F, Cruz-Martinez R, Martinez JM, Figueras F, Gratacos E. Usefulness of myocardial tissue Doppler vs conventional echocardiography in the evaluation of cardiac dysfunction in early-onset intrauterine growth restriction. *Am J Obstet Gynecol* 2010; 203: 45. e1-7.
40. Cruz-Lemini M, Crispi F, Valenzuela- Alcaraz B, et al. Value of annular M-mode displacement versus tissue Doppler velocities to assess cardiac function in intrauterine growth restriction. *Ultrasound Obstet Gynecol* 2013; 42: 175-81.

41. Crispi F, Figueras F, Cruz-Lemini M, Bartrons J, Bijnens B, Gratacos E. Cardiovascular programming in children born small for gestational age and relationship with prenatal signs of severity. *Am J Obstet Gynecol.* 2012 Aug; 207(2): 121.e1-9. doi: 10.1016/j.ajog.2012.05.011. Epub 2012 May 16.
42. Comas M, Crispi F, Gomez O, Puerto B, Figueras F, Gratacos E. Gestational age- and estimated fetal weight-adjusted reference ranges for myocardial tissue Doppler indices at 24-41 weeks' gestation. *Ultrasound Obstet Gynecol* 2011; 37: 57-64.
43. Hecher K, Campbell S, Doyle P, Harrington K, Nicolaides K. Assessment of fetal compromise by Doppler ultrasound investigation of the fetal circulation: arterial, intracardiac, and venous blood flow velocity studies. *Circulation* 1995; 91: 129-138.
44. Makikallio K, Vuolteenaho O, Jouppila P, Rasanen J. Ultrasonographic and biochemical markers of human fetal cardiac dysfunction in placental insufficiency. *Circulation* 2002; 105: 2058-2063.
45. Nassr AA, Yousssef AA, Zakherah MS, Ismail AM, Brost BC. Clinical application of myocardial performance index in the evaluation of fetal growth restruction. *J Perinat Med* 2014; April 4 [epub ahead of print].
46. Cruz-Martinez R1, Figueras F, Hernandez-Andrade E, Oros D, Gratacos E. Changes in myocardial performance index and aortic isthmus and ductus venosus Doppler in term, small-for-gestational age fetuses with normal umbilical artery pulsatility index. *Ultrasound Obstet Gynecol*. 2011 Oct;38(4):400-5. doi: 10.1002/uog.8976. Epub 2011 Jul 26.
47. Hassan WA, Brockelsby J, Alberry M, Fanelli T, Wladimiroff J, Lees CC. Cardiac function in early onset small for gestational age and growth restricted fetuses. *Eur J Obstet Gynecol Reprod Biol.* 2013 Dec; 171(2): 262-5. doi: 10.1016/j.ejogrb.2013.09.020. Epub 2013 Sep 29.
48. Naujorks AA, Zielinsky P, Klein C, Nicoloso LH, Piccoli AL Jr, Becker E, Frajndlich R, Pizzato P, Barbisan C, Busato S, Lopes M. Myocardial velocities, dynamics of the septum primum, and placental dysfunction in fetuses with growth restriction. *Congenit Heart Dis.* 2014 Mar-Apr; 9(2): 138-43. doi: 10.1111/chd.12099. Epub 2013 Jun 10.
49. Gores G, Raith W, Ravekes W, Koestenberger M. Relevance of longitudinal systolic heart function in fetuses with intrauterine growth restriction. *Ultrasound Obstet Gynecol.* 2014 Jan;43(1):108-9. doi: 10.1002/uog.13260.
50. Leipala JA, Boldt T, Turpeinen U, Vuolteenaho O, Fellman V. Cardiac hypertrophy and altered hemodynamic adaptation in growth-restricted preterm infants. *Pediatr Res* 2003;162:426–427.
51. Koestenberger M, Nagel B, Ravekes W, Urlesberger B, Raith W, Avian A, Halb V, Cvirn G, Fritsch P, Gamillscheg A. Systolic right ventricular function in preterm and term neonates: reference values of the tricuspid annular plane systolic excursion (TAPSE) in 258 patients and calculation of Z-score values. *Neonatology* 2011; 100: 85–92.

52. Fouzas S, Karatza AA, Davlouros PA, Chrysis D, Alexopoulos D, Mantagos S, Dimitriou G. Neonatal cardiac dysfunction in intrauterine growth restriction. *Pediatr Res*. 2014 May;75(5):651-7. doi: 10.1038/pr.2014.22. Epub 2014 Feb 12.
53. Verburg BO, Jaddoe VW, Wladimiroff JW, Hofman A, Witteman JC, Steegers EA. Fetal hemodynamic adaptive changes related to intrauterine growth: the Generation R Study. *Circulation* 2008;117:649–59.
54. Rosenberg A. The IUGR newborn. *Semin Perinatol* 2008;32:219–24.
55. Skilton MR, Evans N, Griffiths KA, Harmer JA, Celermajer DS. Aortic wall thickness in newborns with intrauterine growth restriction. *Lancet* 2005; 365:1484-6.
56. Crispi F, Bijnens B, Figueras F, et al. Fetal growth restriction results in remodeled and less efficient hearts in children. *Circulation* 2010;1 21: 2427-2436.
57. Jarvisalo MJ, Jartti L, Nanto-Salonen K, et al. Increased aortic intima-media thickness: a marker of preclinical atherosclerosis in high-risk children. *Circulation* 2001;104:2943-7.
58. Rossi P, Tauzin L, Marchand E, Boussuges A, Gaudart J, Frances Y. Respective roles of preterm birth and fetal growth restriction in blood pressure and arterial stiffness in adolescence. *J Adolesc Health* 2011; 48: 520-522.
59. Clur SAB, Oude Rengerink K, Mol BWJ, Ottenkamp J, Bilardo CM. Fetal cardiac function between 11 and 35 weeks' gestation and nuchal translucency thickness. *Ultrasound Obstet Gynecol* 2011; 37: 48-56.
60. Clur SAB, Oude Rengerink K, Mol BWJ, Ottenkamp J, Bilardo CM. Is fetal cardiac function gender dependent? *Prenat Diagn* 2011; 31: 536-542.
61. Clur SAB, Oude Rengerink K, Ottenkamp J, Bilardo CM. Cardiac function in Trisomy 21 fetuses. *Ultrasound Obstet Gynecol* 2011; 37: 163-71.
62. Lee W, Allan L, Carvalho JS, Chaoui R, Copel J, Devore G, Hechter K, Munoz H, Nelson T, Paladini D, Yagel S. ISUOG consensus statement: what constitutes a fetal echocardiogram? *Ultrasound Obstet Gynecol* 2008; 32: 239-242.
63. Simpson JM, Cook A. Repeatability of echocardiographic measurements in the human fetus. *Ultrasound Obstet Gynecol* 2002; 20: 332-339.
64. Raboisson MJ, Bourdages M, Fouron JC. Measuring left ventricular myocardial performance in fetuses. *Am J Cardiol* 2003; 91: 919-921.
65. Hernandez-Andrade E, Lopez-Tenorio J, Figueroa-Diesel H, Sanin-Blair J, Carreras E, Cabero L, Gratacos E. A modified myocardial performance (Tei) index based on the use of valve clicks improves reproducibility of fetal left cardiac function measurement. *Ultrasound Obstet Gynecol* 2005; 26: 227-232.
66. Lang RM, Bierig M, Devereux RB, et al. Recommendations for chamber quantification: a report from the American Society of Echo- cardiography's Guidelines and Standards Committee and the Chamber Quantification Writing Group, developed in conjunction with the

European Association of Echocardiography, a branch of the European Society of Cardiology. *J Am Soc Echocardiogr* 2005;18:1440-63.

67. Kono T, Sabbah HN, Rosman H, Alam M, Jafri S, Goldstein S. Left ventricular shape is the primary determinant of functional mitral regurgitation in heart failure. *J Am Coll Cardiol* 1992;20: 1594-8.

68. DeVore GR, Siassi B, Platt LD. Fetal echocardiography: IV, M-mode assessment of ventricular size and contractility during the second and third trimesters of pregnancy in the normal fetus. *Am J Obstet Gynecol* 1984;150:981-8.

69. Geva T. Echocardiography and Doppler ultrasound. In Garson A, Bricker JT, Fisher DJ editors. *The science and practice of pediatric cardiology.* Baltimore:Williams and Wilkins; 1998. p834-844.

70. Pacileo G, Paladini D, Pisacane C, Palmieri S, Russo MG, Calabrio R. Role of changing loading condition on atrioventricular flow velocity patterns in normal human fetuses. *Am J Cardiol* 1994; 73: 991-993.

71. Tei C, Ling L, Hodge D, Bailey K, Ok J, Rodenheffer R, Tajik A, Steward J. New index of combined systolic and diastolic myocardial performance: a simple and reproducible measure of cardiac function. – a study in normal and dilated cardiomyopathy. *J Cardiol* 1995; 26: 357-366.

72. St John Sutton M, Gill T, Plappert T , Saltzman DH, Doubilet P. Assessment of right and left ventricular function in terms of force development with gestational age in the normal fetus. *Br Heart J* 1991; 66: 285-289.

73. Bilardo CM, Müller MA, Zikulnig L, Schipper M, Hecher K. Ductus venosus studies in fetuses at high risk for chromosomal or heart abnormalities: relationship with nuchal translucency measurement and fetal outcome. *Ultrasound Obstet Gynecol* 2001; 17: 288-294.

74. Teixeira LS, Leite J, Viegas MJ, Faria MM, Chaves AS, Teixeira RC, Pires MC, Pettersen H. Ductus venosus Doppler velocimetry in the first trimester: a new finding. *Ultrasound Obstet Gynecol* 2008; 31: 261-265.

75. Bilardo CM, Timmerman E, Robles de Medina PG, Clur SA. Low -resistance hepatic artery flow in first trimester fetuses: an ominous sign. *Ultrasound Obstet Gynecol*. 2011; 37: 438-443.

76. SPSS18. SPSS Inc, Chicago, Il, USA.

77. McMillen IC, Adams MB, Ross JT, Coulter CL, Simonetta G, Owens JA, Robinson JS, Edwards LJ: Fetal growth restriction: adaptations and consequences. Reproduction 2001;122:195-204.

78. Hernandez-Andrade E, Figueroa-Diesel H, Jansson T, Rangel-Nava H, Gratacos E: Changes in regional fetal cerebral blood flow perfusion in relation to hemodynamic

deterioration in severely growth-restricted fetuses. Ultrasound Obstet Gynecol 2008;32:71-76.
79. Castillo-Melendez M, Yawno T, Allison BJ, Jenkin G, Wallace EM, Miller SL: Cerebrovascular adaptations to chronic hypoxia in the growth restricted lamb. Int J Dev Neurosci 2015.
80. Murray E, Fernandes M, Fazel M, Kennedy SH, Villar J, Stein A: Differential effect of intrauterine growth restriction on childhood neurodevelopment: a systematic review. BJOG 2015;122:1062-1072.
81. Tanis JC, Boelen MR, Schmitz DM, Casarella L, van der Laan ME, Bos AF, Bilardo CM: Prenatal Doppler flow patterns and neonatal circulation in fetal growth restriction. Ultrasound Obstet Gynecol 2015.
82. Cohen E, Baerts W, Alderliesten T, Derks J, Lemmers P, van Bel F: Growth restriction and gender influence cerebral oxygenation in preterm neonates. Arch Dis Child Fetal Neonatal Ed 2015.
83. Verhagen EA, Kooi EM, van den Berg PP, Bos AF: Maternal antihypertensive drugs may influence cerebral oxygen extraction in preterm infants during the first days after birth. J Matern Fetal Neonatal Med 2013;26:871-876.
84. Jobsis FF: Noninvasive, infrared monitoring of cerebral and myocardial oxygen sufficiency and circulatory parameters. Science 23 December 1977: 1264-1267 [DOI:10 1126/science 929199] 1977:1264.
85. Naulaers G, Meyns B, Miserez M, Leunens V, Van Huffel S, Casaer P, Weindling M, Devlieger H: Use of tissue oxygenation index and fractional tissue oxygen extraction as non-invasive parameters for cerebral oxygenation. A validation study in piglets. Neonatology 2007;92:120-126.
86. Sorensen LC, Greisen G: Precision of measurement of cerebral tissue oxygenation index using near-infrared spectroscopy in preterm neonates. J Biomed Opt 2006;11:054005.
87. Dix L, Lemmers P, Baerts W, van Bel F: Comparing different NIRS devices and their sensors for monitoring regional cerebral oxygen saturation in neonates.
88. Hessel TW, Hyttel-Sorensen S, Greisen G: Cerebral oxygenation after birth - a comparison of INVOS((R)) and FORE-SIGHT near-infrared spectroscopy oximeters. Acta Paediatr 2014;103:488-493.
89. Kurth CD, McCann JC, Wu J, Miles L, Loepke AW: Cerebral oxygen saturation-time threshold for hypoxic-ischemic injury in piglets. Anesth Analg 2009;108:1268-1277.
90. Kurth CD, Levy WJ, McCann J: Near-infrared spectroscopy cerebral oxygen saturation thresholds for hypoxia-ischemia in piglets. J Cereb Blood Flow Metab 2002;22:335-341.

91. Verhagen EA, Van Braeckel KN, van der Veere CN, Groen H, Dijk PH, Hulzebos CV, Bos AF: Cerebral oxygenation is associated with neurodevelopmental outcome of preterm children at age 2 to 3 years. Dev Med Child Neurol 2015;57:449-455.
92. Caicedo A, Naulaers G, Lemmers P, van Bel F, Wolf M, Van Huffel S: Detection of cerebral autoregulation by near-infrared spectroscopy in neonates: performance analysis of measurement methods. J Biomed Opt 2012;17:117003.
93. Verhagen EA, Hummel LA, Bos AF, Kooi EM: Near-infrared spectroscopy to detect absence of cerebrovascular autoregulation in preterm infants. Clin Neurophysiol 2014;125:47-52.
94. Said MM, Niforatos N, Rais-Bahrami K: Validation of near infrared spectroscopy to measure abdominal somatic tissue oxygen saturation in neonates. J Neonatal Perinatal Med 2013;6:23-30.
95. Gillam-Krakauer M, Cochran CM, Slaughter JC, Polavarapu S, McElroy SJ, Hernanz-Schulman M, Engelhardt B: Correlation of abdominal rSO2 with superior mesenteric artery velocities in preterm infants. J Perinatol 2013;33:609-612.
96. Mittnacht AJ: Near infrared spectroscopy in children at high risk of low perfusion. Curr Opin Anaesthesiol 2010;23:342-347.
97. Patel AK, Lazar DA, Burrin DG, Smith EO, Magliaro TJ, Stark AR, Brandt ML, Zamora IJ, Sheikh F, Akinkuotu AC, Olutoye OO: Abdominal near-infrared spectroscopy measurements are lower in preterm infants at risk for necrotizing enterocolitis. Pediatr Crit Care Med 2014;15:735-741.
98. Cerbo RM, Maragliano R, Pozzi M, Strocchio L, Mostert M, Manzoni P, Stronati M: Global perfusion assessment and tissue oxygen saturation in preterm infants: where are we?. Early Hum Dev 2013;89 Suppl 1:S44-6.
99. Schat TE, van der Laan ME, Schurink M, Hulscher JB, Hulzebos CV, Bos AF, Kooi EM: Abdominal near-infrared spectroscopy in preterm infants: a comparison of splanchnic oxygen saturation measurements at two abdominal locations. Early Hum Dev 2014;90:371-375.
100. Keating P, Verhagen E, van Hoften J, ter Horst H, Bos AF: Effect of indomethacin infused over 30 minutes on cerebral fractional tissue oxygen extraction in preterm newborns with a patent ductus arteriosus. Neonatology 2010;98:232-237.
101. Mifsud W, Sebire NJ: Placental pathology in early-onset and late-onset fetal growth restriction. Fetal Diagn Ther. 2014;36(2):117-28.
102. Wareing M, Myers JE, O'Hara M, Baker PN: Sildenafil citrate (Viagra) enhances vasodilatation in fetal growth restriction. J Clin Endocrinol Metab. 2005 May;90(5):2550-5.
103. Maharaj CH, O'Toole D, Lynch T, Carney J, Jarman J, Higgings BD, Morrison JJ, Laffey JG: Effects and mechanisms of action of sildenafil citrate in human chorionic arteries. Reprod Biol Endocrinol. 2009 Apr 23;7:34.

Pels et al. Trials (2019) 20:42
https://doi.org/10.1186/s13063-018-3136-z

Trials

UPDATE Open Access

Detailed statistical analysis plan for the Dutch STRIDER (Sildenafil TheRapy In Dismal prognosis Early-onset fetal growth Restriction) randomised clinical trial on sildenafil versus placebo for pregnant women with severe early onset fetal growth restriction

Anouk Pels[1*], Janus C. Jakobsen[2,3], Wessel Ganzevoort[1], Christiana A. Naaktgeboren[1], Wes Onland[4], Aleid G. van Wassenaer-Leemhuis[4] and Christian Gluud[2]

Abstract

Objective: The objective of the Dutch Sildenafil therapy in dismal prognosis early onset fetal growth restriction (STRIDER) randomised clinical trial is to assess the beneficial and harmful effects of sildenafil versus placebo on fetal and neonatal mortality in pregnant women with severe early-onset fetal growth restriction. The objective of this detailed statistical analysis plan is to minimize the risks of selective reporting and data-driven analysis.

Setting: The setting is 10 tertiary care hospitals and one secondary care hospital in The Netherlands.

Participants: The participants will be 360 pregnant women with severe early-onset fetal growth restriction.

Interventions: The intervention is sildenafil 25 mg or placebo orally three times a day.

Primary and secondary outcome measures: The primary outcome is a composite of death or major neonatal morbidity assessed at hospital discharge. The secondary outcomes are neurodevelopmental impairment; mean scores of the Bayley III cognitive and motor assessment; the proportion of patients experiencing either preeclampsia or haemolysis, elevated liver enzymes, and low platelets syndrome; pulsatility index of uterine arteries, umbilical artery, and middle cerebral artery; birthweight; and gestational age at either delivery or intra-uterine death.

Results: A detailed statistical analysis is presented, including pre-defined exploratory outcomes and planned subgroup analyses. One interim analysis after 180 patients had completed the study was planned and a strategy to minimise the risks of type I errors due to repetitive testing is presented. During review of this manuscript the interim analysis was performed by the Data Safety Monitoring Board and early stopping of the trial was recommended. Final analyses will be conducted independently by two statistically qualified persons following the present plan.

(Continued on next page)

* Correspondence: a.pels@amc.uva.nl
[1]Department of Obstetrics and Gynecology, Amsterdam UMC, University of Amsterdam, Amsterdam, The Netherlands
Full list of author information is available at the end of the article

© The Author(s). 2019 **Open Access** This article is distributed under the terms of the Creative Commons Attribution 4.0 International License (http://creativecommons.org/licenses/by/4.0/), which permits unrestricted use, distribution, and reproduction in any medium, provided you give appropriate credit to the original author(s) and the source, provide a link to the Creative Commons license, and indicate if changes were made. The Creative Commons Public Domain Dedication waiver (http://creativecommons.org/publicdomain/zero/1.0/) applies to the data made available in this article, unless otherwise stated.

(Continued from previous page)
Conclusion: This pre-specified statistical analysis plan was written and submitted without knowledge of the unblinded data and updated after stopping of the trial at interim analysis.

Trial registration: ClinicalTrials.gov, NCT02277132. Registered on 29 September 2014.
Original protocol for the study: doi:https://doi.org/10.5281/zenodo.56148

Keywords: Fetal growth restriction, Placental insufficiency, Sildenafil, Randomised placebo-controlled trial, Statistical analysis plan

Background

The Dutch Sildenafil therapy in dismal prognosis early-onset fetal growth restriction (STRIDER) randomised clinical trial is a blinded trial was recruiting patients recently, assessing the benefits and harms of sildenafil versus placebo in pregnant women with severe early-onset fetal growth restriction (FGR) and their offspring. The primary outcome is mortality and morbidity of the children. Fetal growth restriction is a condition in which a fetus does not reach its designated growth potential and thus is too small for gestational age (SGA), mostly defined as either estimated fetal weight or abdominal circumference determined by ultrasound below the third percentile or gestational age below the tenth percentile. However, no unanimously agreed definition has yet been adopted [1].

The predominant cause of fetal growth restriction, particularly at early onset (< 32 weeks), is placental dysfunction with high resistance, low-flow, placental circulation, due to inadequate spiral artery remodelling early in pregnancy [2]. Depending on the gestational age at development, the fetus has a substantial risk of mortality and morbidity [3]. As the phosphodiesterase 5- (PDE5-) inhibitor sildenafil causes vasodilatation, it might improve the utero-placental circulation in fetal growth restriction resulting in improved growth and increased chances of healthy survival of the fetus [4–20].

A recent meta-analysis of sildenafil in fetal growth restriction has been published [21]. This meta-analysis included only one randomised clinical trial of sildenafil in which a single administration of 50 mg sildenafil versus placebo was given to pregnant women with fetal growth restriction between 24 and 37 weeks of gestation [22]. An improvement of the Doppler measurements of the umbilical artery and middle cerebral artery was seen in the sildenafil group compared with the placebo group [22]. However, no patient-centred or clinically relevant outcomes (such as morbidity and mortality) were assessed and patients only received a single dose of sildenafil. The review, furthermore, described a non-randomised comparative study in which 10 women received sildenafil 25 mg three times a day compared to 17 women without sildenafil administration [23]. This observational study indicated an increase in fetal abdominal circumference growth and a trend toward better survival in the sildenafil group compared to the group that was untreated [23]. The review does not identify other clinical trials of sildenafil in fetal growth restriction and concludes that more randomised clinical trials are needed [21].

Besides the short-term randomised clinical trial and the observational study mentioned above, we identified one recently published clinical trial where 35 patients with fetal growth restriction were randomised to three groups, receiving either oral sildenafil, transdermal nitroglycerin, or oral placebo [24]. The outcomes were non-validated surrogate outcomes [25], i.e. Doppler ultrasound measurements of the uterine arteries, umbilical artery, and middle cerebral artery were evaluated after administration of the trial interventions. Positive effects of sildenafil and nitroglycerin were seen in the pulsatility index of the uterine artery and the umbilical artery, while no effect was seen in the placebo group [24].

A couple of randomised clinical trials on sildenafil have been conducted in women with diagnosed preeclampsia. A randomised clinical trial including 100 women with preeclampsia showed a statistically significant difference in pregnancy prolongation of 4 days in favour of the sildenafil group compared with the placebo group [26]. In another randomised clinical trial, 35 patients with preeclampsia received sildenafil in increasing dose versus placebo. This trial did not find a significant difference in pregnancy prolongation after treatment with sildenafil compared with placebo [12].

Apart from sildenafil, interest has also focused on L-arginine, which is an amino-acid that interacts in the same pathway as sildenafil and theoretically could have a similar clinical effect. The aforementioned meta-analysis of Chen and colleagues included eight randomised clinical trials and one quasi-randomised study (total 576 patients) assessing L-arginine versus placebo or no therapy [21]. The analysis showed that L-arginine seems to have a significant beneficial effect on birthweight, gestational age at delivery, intracranial haemorrhage, and neonatal respiratory distress syndrome [21]. However, the authors of the meta-analysis state that four of the nine studies were of uncertain quality and there is a high risk of bias

[27–30]. Furthermore, the number of randomised patients in the trials is relatively small.

By reviewing the existing literature, high-quality evidence is pending for a pharmacological treatment of fetal growth restriction. Apart from the Dutch STRIDER, four other STRIDER trials are presently conducted or are in different phases of preparation, recruitment, and analysis [31]. The results of the UK STRIDER trial have been published recently [32] and did not show a difference in pregnancy prolongation between patients allocated to sildenafil versus placebo. To minimise the risks of selective reporting and data-driven analyses, we will here shortly describe the plans for interim analysis and in detail our statistical analysis plans of the Dutch STRIDER trial and how the results will be reported. At first submission of this manuscript, the Dutch STRIDER trial was still recruiting patients and collecting the data; however, during the review of this manuscript, the trial was stopped early based on advice of the DSMB.

Trial overview

Please see the published protocol of the trial for a detailed description of the methodology [33]. In short, the Dutch STRIDER trial compares 25 mg sildenafil three times daily orally with matching placebo three times daily in women with severe early-onset fetal growth restriction. The placebo matches the sildenafil in form, size, colour, smell, and solubility. The patients eligible for inclusion are women from 20 weeks and 0 days of gestation until 29 weeks and 6 days, with fetal growth restriction and signs of placental insufficiency, without an alternative explanation for the fetal growth restriction. Participants will use study medication until 32 weeks of gestation or delivery, whichever comes first. The participants, the treatment providers, the outcome assessors, the statisticians, and the conclusion drawers were planned to be blinded for the treatment allocation [27, 28, 34–40]. The treatment allocation was unblinded on early stopping of the trial. The participants, treatment providers, and outcome assessors were blinded up to stopping the trial at the interim analysis.

The original protocol of the Dutch STRIDER trial was approved by the local ethical committee on 22 July 2014. The first patient was included on 20 January 2015. The trial was conducted according to the principles of the Declaration of Helsinki Medical, Dutch legislation on medical research involving human subjects [41–44] and good clinical practice guidelines (GCP) [45]. Patients could only be included in the trial after written informed consent from the pregnant woman was obtained. All study sites are monitored by an independent clinical research associate of the Nederlandse Vereniging voor Obstetrie en Gynaecologie Consortium. An independent data safety monitoring board (DSMB) monitored the study progress, with a special focus on safety (see below). The trial will be reported according to the Consolidated standards of reporting trials (CONSORT) guidelines [46].

Intervention period and data collection

The intervention is sildenafil 25 mg three times daily orally versus placebo three times daily up to 32 weeks gestation or delivery, whichever comes first. Clinical outcome data will be recorded from mother and neonate until discharge to home. Follow up of the child will be assessed at 2 years of age in an outpatient setting.

Concomitant treatments

Patients who participate in the Dutch STRIDER trial will furthermore be treated according to local protocol. The caregivers, blinded to the allocated therapy, will make decisions on the administration of corticosteroids for fetal lung maturity at the moment of delivery, based on fetal and maternal condition and maternal treatment of hypertensive disorder, according to the clinical practice in that particular centre, as if patients were not participating in a trial.

Baseline variables

The baseline criteria that are considered to be relevant and are planned to be reported are listed in Table 1. The baseline characteristics will be presented by treatment allocation. Binary and categorical outcomes will be expressed in frequencies and percentages. In the case of missing data, there will be a note on how many data were available. Continuous variables will be expressed by either mean and standard deviation (normal distribution) or median and IQR (non-normal distribution). Differences in the treatment arms will not be statistically tested.

Data collection and storage

Data management was implemented according to GCP guidelines. Patient data up to hospital discharge and long-term follow up data are entered via an electronic case record form (CRF) in a central GCP-proof web-based database to facilitate on-site data entry (RedCap). Security is guaranteed with login names, login codes, and encrypted data transfer. Data collection is performed at multiple time points: at the time of inclusion and randomisation, during the study medication treatment period, at hospital discharge of the child, and at 2 years of corrected age for follow up. Data on eligible patients not included in the study are also recorded, including patient characteristics and the primary outcome (death or survival with major morbidities).

Serum placental growth factor (PlGf) will be analysed after completion of the study. The PlGf analysis currently

Table 1 Baseline criteria

	Sildenafil (*n* =)	Placebo (*n* =)
Age (years)		
BMI (kg/m^2)		
Ethnicity		
Caucasian (%)		
African descent (%)		
Asian (%)		
Other (%)		
Highest completed educational level mother		
High (%)		
Middle (%)		
Low (%)		
Unknown (%)		
Highest completed educational level father/partner		
High (%)		
Middle (%)		
Low (%)		
Unknown (%)		
Language spoken at home		
Only Dutch		
Only other language than Dutch		
More than one language, including Dutch		
Maternal smoking (%)		
Gestational age at inclusion (weeks + days)		
Estimated fetal weight at ultrasound (gram)		
Fetal abdominal circumference at ultrasound (mm)		
Notching uterine artery (one-or two-sided) (%)		
PI umbilical artery > 95th centile (%)		
PI middle cerebral artery < 5th centile (%)		
End-diastolic flow		
Positive (%)		
Absent (%)		
Reversed (%)		
Pregnancy-induced hypertension (%)		
Preeclampsia (%)		
HELLP syndrome (%)		
Systolic blood pressure (mmHg)		
Diastolic blood pressure (mmHg)		

BMI body mass index, *PI* pulsatility index, *HELLP* haemolysis, elevated liver enzymes, and low platelets syndrome

is not part of standard care and is not often performed. To investigate the predictive value of PlGf for adverse outcomes in FGR, blood serum samples at inclusion are collected and stored. Samples will not be used before the inclusion of participants in the study and data collection is complete.

Primary outcome

The primary outcome is a composite outcome consisting of either:

1. Neonatal mortality assessed at the time point when the neonate is discharged from the hospital or
2. Major neonatal morbidity defined as
   – Intraventricular haemorrhage (IVH) grade 3 or more or
   – Periventricular leukomalacia (PVL) grade 2 or more or
   – Moderate or severe bronchopulmonary dysplasia (BPD) or
   – Necrotising enterocolitis (NEC) grade 2 or more or
   – Retinopathy of prematurity (ROP) treated by surgery or laser therapy

- Intraventricular haemorrhage (IVH) and periventricular leukomalacia (PVL) will be assessed in neonates were born at a gestational age < 32 weeks or with birth weight < 1500 g. These neonates will have an ultrasound scan of the brain as standard. Brain magnetic resonance imaging (MRI) will be performed in case different types of abnormalities are seen on ultrasound or in the clinical behaviour of the neonate. The timing and the number of investigations is dependent on the gestational age at birth, the abnormalities seen, and the clinical behaviour of the neonate. Investigations will be performed according to Dutch national recommendations [47]. If a neonate is evaluated by ultrasound, the scan showing the most severe abnormalities will be used to assess neurological morbidity. If a neonate does not have an ultrasound scan because it is born (near-)term and there is no clinical suspicion of neurological morbidity, this will be diagnosed as “no neurological morbidity”.
- Bronchopulmonary dysplasia is assessed at 36 weeks postmenstrual age (PMA) according to the Dutch guideline for BPD and the National Institute of Child Health and Human Development (NICHD) consensus statement using the classification of severity and, if indicated, the oxygen reduction test as described by Walsh et al. [48–53]. Neonates that will be born after 36 weeks gestational age will be diagnosed as “no bronchopulmonary dysplasia”.
- Retinopathy of prematurity (ROP) screening will take place according to the Dutch guideline for ROP

[54]. Screening will be performed by an ophthalmologist in neonates born < 30 weeks gestational age and/or with birthweight < 1250 g. Neonates born between 30 and 32 weeks and with birthweight between 1250 and 1500 g will in some situations be screened for retinopathy of prematurity as well. The timing and number of assessments is dependent on the gestational age at birth and the abnormalities found at assessment. Neonates that will not be screened for ROP according to the guideline, will be diagnosed as "no retinopathy of prematurity".

- Necrotising enterocolitis is a clinical diagnosis and staging will be according to the Bell system [55]. Whether a neonate will have had an episode of necrotising enterocolitis requiring surgery will be assessed and reported at the time of discharge from the neonatal intensive care.

Secondary outcomes

The secondary outcomes are:

1. The proportion of neonates with neurodevelopmental impairment at 2 years of age, assessed on the two-year Bayley scales of infant development (BSID)-III [56]. Neurodevelopmental follow up will be at the outpatient clinic at the corrected age of the infant of 2 years (2 years after the term age), which is standard in The Netherlands for children born < 30 weeks gestation or born with weight < 1000 g. Neurodevelopmental impairment will be defined using two measures: first, as a cognitive Bayley III score < 85 (or an estimated cognitive delay of more than 3 months when a Bayley test cannot be carried out), composite motor score < 85, cerebral palsy, with a Gross Motor Function Classification System (GMFCS) grade > 1, hearing loss needing hearing aids, or severe visual loss (legally certifiable as blind or partially sighted). The second definition of NDI is similar except it does not include the motor score < 85. Second, we will describe the different components of the composite outcome, including all cases of CP and their GMFCS classifications.
2. The mean composite cognitive Bayley III score (continuous outcome), assessed at the 2-year Bayley scales of infant development BSID-III [56].
3. The mean composite motor score for the Bayley scales of infant development BSID-III [56], and the mean standard scores on the fine and gross motor subscales.
4. The proportion of mothers experiencing either preeclampsia or haemolysis, elevated liver enzymes, and low platelets (HELLP) syndrome. Preeclampsia is defined as hypertension in combination with proteinuria. Hypertension is defined as systolic blood pressure > 140 mmHg and/or diastolic blood pressure > 90 mmHg (Korotkoff V), measured at least twice, after 20 weeks of gestation in a patient that had no hypertension before. Proteinuria is defined as ≥ 300 mg protein measured on 24-h urine collection [57]. HELLP syndrome is defined as elevated lactate dehydrogenase (LDH); either elevated aspartate aminotransferase (AST) or alanine aminotransferase (ALT); and low platelets, according to local laboratory reference values [58]. Second, the proportion of patients with preeclampsia and the proportion of patients with HELLP syndrome will be reported individually as well.
   Whether or not a patient will have had preeclampsia or HELLP syndrome will be assessed when the mother is discharged to go home after delivery. Development of preeclampsia or HELLP syndrome after discharge home for which readmission is necessary will be considered as a serious adverse event (SAE) and will be line-listed, as described in "Severe adverse events".
5. Pulsatility index of umbilical artery: we will use the first pulsatility index measured on ultrasound performed > 24 h after starting study medication.
6. Birthweight (grammes): we will separately describe the birthweight of live-born neonates and the birthweight of fetuses that experienced intra-uterine death.
7. Gestational age of either delivery or intra-uterine death (weeks and days).

Exploratory outcomes

The relevant exploratory outcomes we plan to report, are listed in Table 2 for mother and fetus/neonate.

The percentage of infants that have been assessed for each particular diagnosis will be described for all neonatal outcomes. A table will be presented with line-listing of the primary causes of neonatal death as well. Frequencies and the proportion of total neonatal deaths will be reported.

Severe adverse events

Severe adverse events (SAEs) were pre-defined as any medical occurrence that results in death, is life-threatening, causes or prolongs hospital admission, results in persistent or significant disability or incapacity, or results in congenital anomaly. Due to the characteristics of the included patient group, mortality, morbidity, and hospital admission are common. In the study protocol maternal and fetal/neonatal SAEs were divided into a group of "context-specific" and "non--context-specific" SAEs. Fetal/neonatal context-specific SAEs consist of the events that are explained by and related to the

prematurity and dysmaturity due to fetal growth restriction, for example intra-uterine death, neonatal death due to complications of prematurity/dysmaturity. Non-context-specific SAEs will be considered to be unfavourable events that are not explained by the prematurity/dysmaturity as a result of the fetal growth restriction. Hospital admission for delivery, hypertensive disorders or fetal monitoring will be considered as context-specific maternal SAEs. Other maternal SAEs will be considered to be non-context-specific. All SAEs are evaluated by the Data Monitoring Committee: the context-specific SAEs are monitored during the safety analysis and performed after every 50 patients that completed the study. Non-context-specific SAEs will be sent to and evaluated by the committee right away.

Due to the character and the expected high prevalence of SAEs we did not define SAEs as primary or secondary outcome and will not perform statistical testing on the SAEs, but report them through line-listing.

Adverse effects

Patients are asked to keep note of the adverse effects they experience during the use of study medication in order to evaluate the percentage of women experiencing adverse effects and evaluate the character of experienced adverse effects.

Subgroup analysis

Pre-defined subgroup analyses are:

- An abnormal or normal serum level of placental growth factor (PlGF), defined as PlGF < 5th percentile of the reference value and ≥ 5th percentile of the reference value
- Placental growth factor (PlGF) < 25th percentile of all samples of the study population and PlGF ≥ 25th percentile of all samples of the study population
- Gestational age at inclusion, categorized as < 25 weeks of gestation and ≥ 25 weeks of gestation.
- Estimated fetal weight (EFW) at inclusion, categorised as < 300 g, 300–599 g, and ≥ 600 g.
- Neonates that appear to have a congenital anomaly, which was not known in the antenatal period, and thus at the time of randomisation, will be included in the final analysis. However, we propose a subgroup analysis in this group of patients and if we find a significant difference in the primary outcome of these neonates, we will consider excluding them.

We plan to perform a prognostic study and aim to have the methodology published in a separate statistical analysis plan.

Stratification and design variables

The only stratification variable in the randomisation will be trial site (hospital). 11 Hospitals participated in the study.

Sample size and power estimations

The sample size of the Dutch STRIDER trial has been previously estimated [59]. With an acceptable risk of type I error of 5% and risk of type II error of 80% we aim to investigate a decrease on the primary outcome from 71% [23] in the control group to 56% in the experimental group, which is equal to a relative risk reduction just above 21%. Allowing for one interim analysis according to the O'Brien-Fleming spending function ($p <$ 0.005), 175 women are needed per group. This sensitivity analysis was taken into account in the sample size analysis, if the anticipated inclusion target is reached the final analysis will still powered at 80% to test at a significance level of 0.05. We will include an extra 10 women to account for loss to follow up. The total sample size has been modified to 360 women.

A total of 796 patients will be participating if all STRIDER trials include the number of patients indicated in the sample size calculations. With this number of participants, we will have 80% power to detect a difference of 8.6% in the primary outcome between the intervention and placebo group, having a risk of 5% type I error.

Power estimations for secondary outcomes: based on the estimated sample size of 360 women and an acceptable risk of type I error of 5%, we estimated the statistical power of the secondary outcomes:

1. Neurodevelopmental impairment: 60% power to confirm or reject an increase in neurodevelopmental impairment from 10% [60] in the control group to 20% in the experimental group, equal to a relative risk reduction of just above 21%, having a risk of 5% for type I error.
2. Bayley III score: 80% power to confirm or reject a minimal relevant difference of 5.5 points on the mean composite motor score of the Bayley scales of infant development BSID-III [56], when assuming that 148 children will be alive at 2 years of age and that the mean composite score in the placebo group is 99 (SD 12), with an acceptable risk of 5% for type I error [60].
3. The proportion of mothers experiencing either preeclampsia or HELLP syndrome: 80% power to detect an increase from 50% [23, 26, 61] in the placebo group to 65% in the sildenafil group.
4. Pulsatility index (PI) of the umbilical artery: 80% power to confirm or reject a mean difference of

Table 2 Maternal and fetal/neonatal outcomes

	Intention to treat			Intention to treat, adjusted for GA and EFW at inclusion			Per protocol		
	Sildenafil (*n* =)	Placebo (*n* =)	*P* value	Sildenafil (*n* =)	Placebo (*n* =)	*P* value	Sildenafil (*n* =)	Placebo (*n* =)	*P* value
Maternal outcomes									
Treatment duration (days)									
Gestational age at delivery (weeks + days)									
Pregnancy prolongation after randomisation (days)									
Abdominal circumference at ultrasound closest to 2 weeks after randomisation (mm)									
Mode of delivery									
Caesarean section on fetal indication (%)									
Caesarean section on maternal indication (%)									
Induced vaginal delivery on fetal indication (%)									
Induced vaginal delivery on maternal indication (%)									
Spontaneous vaginal delivery (%)									
Induction of labour after intra-uterine death (%)									
Pregnancy induced hypertension (%)									
Preeclampsia (%)									
HELLP syndrome (%)									
Maternal use of antihypertensive treatment antenatal or postnatal									
One antihypertensive									
Two antihypertensives									
Three or more antihypertensives									
Maternal need for magnesium sulphate for hypertension (%)									
Neonate born between 48 h and 14 days after antenatal corticosteroids course (complete course) (%)									
Neonate born between 0 and 48 h after antenatal corticosteroids course (incomplete course) (%)									
Neonate born during maternal administration of intravenous magnesium sulphate (%)									
Fetal/neonatal outcomes									
Intra-uterine death (%)									
Neonatal death (%)									
Survival at hospital discharge (%)									
Survival with relevant morbidity at hospital discharge (%)									
Survival without relevant morbidity at hospital discharge (%)									
Birth weight of neonates with intra-uterine death (grammes)									
Birth weight of neonates with live birth (grammes)									
Postmenstrual age at first discharge home (weeks + days)									
IVH grade III or IV (%)									
PVL grade II or more (%)									
Moderate or severe BPD (%)									

Table 2 Maternal and fetal/neonatal outcomes *(Continued)*

	Intention to treat			Intention to treat, adjusted for GA and EFW at inclusion			Per protocol		
	Sildenafil (*n* =)	Placebo (*n* =)	*P* value	Sildenafil (*n* =)	Placebo (*n* =)	*P* value	Sildenafil (*n* =)	Placebo (*n* =)	*P* value
No BPD (%)									
ROP treated by laser or surgery (%)									
One or more culture-proven episode of infection or clinical episode of infection with antibiotic treatment necessary ≥ 5 days (%)									
NEC grade II or more (%)									

GA gestational age, *EFW* estimated fetal weight, *HELLP* haemolysis, elevated liver enzymes, and low platelets syndrome, *IVH* intraventricular hemorrhage, *PVL* periventricular leukomalacia, *BPD* bronchopulmonary dysplasia, *ROP* retinopathy of prematurity, *NEC* necrotising enterocolitis

0.03 in PI, when assuming that PI before sildenafil administration is 1.13 (SD 0.10) [22] with an acceptable risk of 5% for type I error.

5. Birthweight (grammes): 80% power to confirm or reject a mean difference of 45 g in the birthweight, when assuming the mean birthweight in the placebo group is 422 g (SD 159) with an acceptable risk of 5% for type I error [23].
6. Gestational age at either delivery or intra-uterine death: 94% power to confirm or reject a mean difference of one week in the gestational age at delivery (SD 2.7 weeks [26]).

Interim analysis

Safety analyses are planned after every 50 patients completing the trial (defined as hospital discharge of the neonate) in which no statistical testing will be performed. The Data Safety Monitoring Committee (DSMB) consists of gynecologists and neonatologist and an independent statistician [62]. One interim analysis is planned after outcomes are available for the first half of the anticipated 180 patients have completed the trial. During the interim analysis, the trial will be stopped if a significant difference in primary outcome between the two treatment arms is observed ($p < 0.005$ according to the O'Brian-Fleming rule) [63]. The study can be stopped at any time in case the safety of the patients or the fetus is considered to be in danger. Also, evidence from other trials and data from the ongoing STRIDER trials will be considered during interim analysis [64].

Statistical analysis

Data on all outcomes will be analysed by two independent statisticians blinded to treatment allocation. Two independent statistical reports will be sent to a third statistician and if there are discrepancies, then the three statistical experts will discuss possible reasons and identify the most correct result.

General analysis principles

The analysis of the Dutch STRIDER trial will be an intention-to-treat analysis, including all patients randomised in the trial. Random intercept models will be used for all primary analyses to account for a centre effect. This method assumes that the effect is constant across the centres, but that the background risks differ. Additionally, we will secondly also adjust all primary analyses for design variables by adding them to the regression model. The design variables will be estimated fetal weight at inclusion and gestational age at inclusion. The course of pregnancy can be difficult to predict. In some women, there will unexpectedly be signs of fetal distress or worsening of the maternal condition due to a hypertensive disorder and therefore emergency delivery might be necessary, even before starting study medication. Therefore, a per-protocol analysis is planned as well, including only patients that used at least one tablet of study medication.

STATA 15 will be used for the statistical analysis and analysis is planned to follow the 5-step procedure for evaluation of intervention effects in randomised clinical trials, as proposed by Jakobsen et al. [65]. The five steps consist of (1) reporting the confidence intervals and the exact *P* values for the primary, secondary, and exploratory outcomes; (2) reporting Bayes factor for the primary outcome; (3) adjusting the confidence intervals and the statistical significance threshold if the trial is stopped early or if interim analyses have been conducted [66, 67]; (4) adjusting the confidence intervals and the *P* values for multiplicity due to number of outcome comparisons; and (5) assessing clinical significance of the trial results.

We plan to publish the results of the trial in a primary publication, reporting the primary and secondary outcomes

assessed at discharge home of the neonate. The results of the 2-year neurodevelopmental assessment will be published separately.

The Bayes factor is the ratio between the probability of obtaining the result assuming the null hypothesis (H_0) is true divided by the probability of obtaining the result assuming the alternative hypothesis (H_A) is true. This factor will be calculated, as the *P* value may be misleading in the case of a low probability of the trial results being compatible with the hypothetical intervention effect in the sample size calculation, even though the *P* value is below the pre-specified threshold [68]. A result < 1.0 supports the conclusion that the sildenafil improves healthy survival in fetal growth restriction, while a Bayes factor > 1.0 supports the inverse conclusion. The suggested threshold in the literature is 0.1 for Bayes factor as an indicator of a high probability of an intervention effect similar to or even greater than the hypothetical intervention effect used in the sample size calculation.

Dichotomised outcomes will be presented as proportions of participants in each group with the event, and risk ratios with 95% confidence intervals. Relative risks will be analysed using generalised linear models (bireg) using a log link function [69]. Additionally, absolute risk reductions and number needed to treat will be presented for interpretability.

Continuous outcomes will be presented as means, standard deviations, and 95% confidence intervals or medians and interquartile ranges for each group and mean differences, standard deviations, and 95% confidence intervals for the difference between the groups. Continuous outcomes will be analysed using linear regression.

Missing data

In the case of missing data, we will follow the principles described by Jakobsen et al. [70] and decide how to handle missing data based on the type of variable or outcome, type of missingness, and proportion of missing data. Either complete case analysis or single or multiple imputation are possible solutions for missing data.

As we expect to have some missing data on the secondary outcome of neurodevelopment, we expect to perform imputation on this outcome. Imputation will not be performed for baseline criteria.

Outline of figures and tables

Figure 1 will be the CONSORT diagram with the flow chart of eligible and randomised patients.

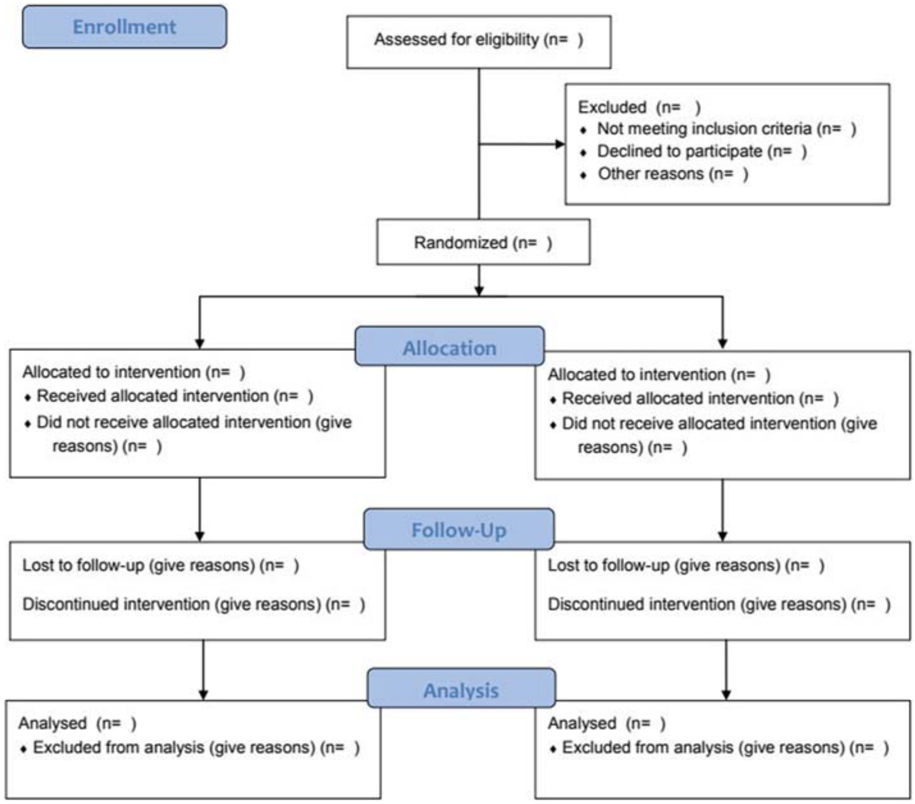

Fig. 1 CONSORT 2010 flow diagram

Table 3 Doppler measurements at inclusion and first measurement > 24 h after start medication

	Sildenafil (*n* =)		Placebo (*n* =)	
	At inclusion	After starting medication	At inclusion	After starting medication
Mean PI uterine artery				
PI umbilical artery				
PI middle cerebral artery				
PI ductus venosus				

PI pulsatility index

Table 1 will be the table with baseline criteria. The maternal and fetal/neonatal outcomes will be expressed in Table 2, showing both the intention-to-treat and the per-protocol analysis. The neonatal outcomes will not be available for all patients, as some patients will have died before assessing a certain variable, for example bronchopulmonary dysplasia, which is assessed at 36 weeks of gestation. In the table will be noted how many neonates have been assessed for that specific variable. A table will be presented with line-listing of the primary causes of neonatal death as well. Frequencies and proportion of total neonatal deaths will be shown.

Table 3 will express the Doppler measurements at inclusion and first measurement after starting medication (at least 24 h after starting medication) will be expressed for treatment allocation and will only show the women who at least had one Doppler measurement after inclusion.

Non-context-specific maternal and fetal/neonatal SAE's in both treatment groups will be line-listed in a table (Table 4) and the maternal side effects of the study medication will be expressed in Table 5 per treatment allocation. Table 6 will express the 2-year neurodevelopmental outcomes and Table 7 the physical outcomes at 2 years. Tables 6 and 7 will not be part of the primary publication, but will be published separately.

Changes between the protocol and the statistical analysis

The primary outcome in the original protocol is stated as "intact survival at term age". For the purpose of the analysis we will express the primary outcome as a composite outcome of mortality and survival with major morbidity. In the outcome table the distinction will be made between the proportion of patients that have intra-uterine death and that have neonatal death. Also, survival without major morbidity and the proportions of neonates surviving with the different morbidities including the grades will be reported separately.

Other changes between the original protocol and the proposed statistical analysis presented here are the sample size calculation, as the stopping rule was changed from Haybittle- Peto to the Lan-DeMets-O'Brian Fleming-rule to avoid early stopping of the trial if sildenafil seems to be more effective than placebo [67].

Table 4 Line-listing of non-context specific SAEs

	Sildenafil (*n* =)	Placebo (*n* =)
Maternal		
…		
…		
Other, namely: …		
Fetal/neonatal		
…		
…		
Other, namely: …		

SAE serious adverse event

Patient and public involvement

The development of the research question, outcome measures, and trial design was based on expert consensus in an international collaboration [31]. No patients were involved in the design stage of the randomised controlled trial. However, patient representatives of the relevant patient organizations were consulted for the funding application and they eagerly supported the trial and recommended it for funding. No patients were involved in the recruitment to and conduct of the study. After completion of the study, study participants will be informed by the study team about the results and the drug allocation received. The burden of the intervention was not assessed by patients themselves. The dissemination of the results will also be through the relevant patient organisations.

Table 5 Adverse effects of study medication

	Sildenafil (*n* =)	Placebo (*n* =)
Headache (%)		
Flushing (%)		
Stuffy nose (%)		
…		
Other		

Table 6 Two-year neurodevelopmental outcomes

	Intention to treat			Intention to treat, adjusted for GA and EFW at inclusion			Per protocol		
	Sildenafil (*n* =)	Placebo (*n* =)	*P* value	Sildenafil (*n* =)	Placebo (*n* =)	*P* value	Sildenafil (*n* =)	Placebo (*n* =)	*P* value
Cognitive composite score (mean)									
Motor score (mean)									
Fine motor score (mean)									
Gross motor score (mean)									
Bayley III cognitive composite score and motor score									
< 70									
70–84									
85–99									
≥ 100									
Bayley III motor composite score and motor score									
< 70									
70–84									
85–99									
≥ 100									
Cerebral palsy, all*									
GMFCS grade 1									
GMFCS grade 2									
GMFCS grade 3									
GMFCS grade 4									
GMFCS grade 5									
Normal vision									
Impaired vision despite glasses or lenses									
Mildly abnormal vision despite glasses or lenses									
No useful vision									
Strabismus or amblyopia with normal (corrected) vision									
Normal hearing									
Subnormal hearing for those cases that do need aids and have mild hearing loss at time of testing at age 2 years (i.e. mostly conductive in origin)									
Hearing loss (partly) corrected with aids									
Hearing loss not corrected with aids									
Normal communication									
No normal communication									
Growth									
height mean z-score, corrected age									
Weight mean z-score corrected age BMI z-score corrected age									
Head circumference mean z-score corrected age									
Neurodevelopmental impairment I and II**									

GA gestational age, *EFW* estimated fetal weight, *GMFCS* Gross Motor Function Classification System
*We will score all cerebral palsy (CP) cases and then subdivide them in GMFCS levels; a child that does not have CP will not have a GMFCS score
**Defined as either a cognitive Bayley III score < 85 or estimated cognitive delay > 3 months, cerebral palsy, with a GMFCS > 1, hearing loss needing hearing aids, or severe visual loss (legally certifiable as blind or partially sighted)

Table 7 Physical outcomes at 2 years

	Intention to treat			Intention to treat, adjusted for GA and EFW at inclusion			Per protocol		
	Sildenafil (*n* =)	Placebo (*n* =)	*P* value	Sildenafil (*n* =)	Placebo (*n* =)	P value	Sildenafil (*n* =)	Placebo (*n* =)	P value
Number of readmissions since primary discharge									
Number of surgery procedures since primary discharge									
Number of medications used in last year									
Current medication use									

Current trial status

At the moment of submission of this manuscript, the number of inclusions was 186, which corresponds to 52% of anticipated sample size. However, during interim analysis performed on 19 July 2018, evaluating the results of the first 183 patients, the DSMB had advised stopping the trial due to safety concerns and a lack of evidence of positive effects. At that time, 216 patients (60% of anticipated sample size) were recruited in in the trial. The patients that were still using study medication stopped taking the tablets. The treatment allocation of all patients was unblinded and was seen by the researchers. This manuscript was submitted on 15 March and was under review.

Despite the smaller sample size and early unblinding of the drug allocation, we will try as much as possible to perform the analyses according to the previously described statistical analysis plan. The consequence is that our study might not have enough power for the primary and all of the secondary outcomes. The performance of the previously planned IPD meta-analysis with the other STRIDER trials will become more important. We plan to analyse patients that stopped taking the study medication due to the stopping of the trial, in both the intention-to-treat and in the per-protocol analyses. However, we will perform subgroup analysis in which we will exclude these patients to see whether this will change the primary and secondary outcomes significantly.

Discussion

With the described statistical analysis plan we tried to minimise the risks of reporting bias and data-drive analysis in reporting the main results of the Dutch STRIDER trial. We described the pre-defined baseline criteria and primary and secondary outcomes and the analysis plan per outcome.

Four other STRIDER trials with similar inclusion criteria, intervention, and outcome measures are undertaken simultaneously. By performing an individual patient data (IPD) meta-analysis over the results of the five trials, more reliable conclusions can be drawn than from this single trial. However, until all the trials have been performed and individually analysed, we hope that the described statistical approach for the Dutch STRIDER trial will help give a temporary answer to the question of whether or not sildenafil increases the chance of healthy survival in women with severe early-onset fetal growth restriction and whether or not this therapy needs to be applied in clinical practice.

Conclusions

The Dutch STRIDER trial investigates if sildenafil compared with placebo increases the chance of intact neonatal survival at term age in pregnancies complicated by fetal growth restriction. The present statistical analysis plan for the main outcomes of this trial is presented to minimise the risk of reporting bias and data-driven analysis. The results may have profound effects on the health and quality of life of 700–900 patients in The Netherlands each year, and globally the number could be 700,000 patients.

Abbreviations

ALT: Alanine aminotransferase; AST: Aspartate aminotransferase; BMI: Body mass index; BPD: Bronchopulmonary dysplasia; BSID: Bayley Scales of Infant Development; CONSORT: Consolidated standards of reporting trials; CP: Cerebral palsy; CRF: Case record form; DSMB: Data safety monitoring board; EFW: Estimated fetal weight; FGR: Fetal growth restriction; GCP: Good clinical practice; GMFCS: Gross Motor Function Classification System; HELLP: Haemolysis, elevated liver enzymes, and low platelets syndrome; IQR: Inter quartile range; IVH: Intraventricular hemorrhage; LDH: Lactate dehydrogenase; Mg: Milligram; MRI: Magnetic resonance imaging; NDI: Neurodevelopmental impairment; NEC: Necrotising enterocolitis; NICHB: National Institute of Child Health and Human Development; PDE-5: Phosphodiesterase-5; PI: Pulsatility index; PlGf: Placental growth factor; PVL: Periventricular leukomalacia; ROP: Retinopathy of prematurity; SAE: Severe adverse event; SD: Standard deviation; SGA: Small for gestational age; STRIDER: Sildenafil therapy in dismal prognosis early-onset fetal growth restriction

Acknowledgements

None.

Funding

The STRIDER trial is funded by ZonMW 80–83600–98-20081.

Availability of data and materials

The authors aim to share the results of the randomised clinical trial upon request. Of the current detailed statistical analysis plan no original data are available.

Authors' contributions
Designing the original study protocol: AP, WG, CAN, WO. Preparing manuscript: AP, JCJ, WG, CAN, WO, AGWL, CG. All authors read and approved the final manuscript.

Ethics approval and consent to participate
The original protocol of the Dutch STRIDER trial was approved by the local ethical committee of Academisch Medisch Centrum on 22 July 2014. Reference number 2014_131.

Consent for publication
Not applicable.

Competing interests
The authors declare that they have no competing interests.

Publisher's Note

Springer Nature remains neutral with regard to jurisdictional claims in published maps and institutional affiliations.

Author details
[1]Department of Obstetrics and Gynecology, Amsterdam UMC, University of Amsterdam, Amsterdam, The Netherlands. [2]The Copenhagen Trial Unit, Centre for Clinical Intervention Research, Rigshospitalet, Copenhagen University Hospital, Copenhagen, Denmark. [3]Department of Cardiology, Holbæk Hospital, Holbaek, Denmark. [4]Department of Neonatology, Emma Children's Hospital/Academisch Medisch Centrum, Amsterdam, The Netherlands.

Received: 12 March 2018 Accepted: 14 December 2018
Published online: 11 January 2019

References

1. Gordijn SJ, et al. Consensus definition of fetal growth restriction: a Delphi procedure. Ultrasound Obstet Gynecol. 2016;48(3):333–9.
2. Nardozza LM, et al. Fetal growth restriction: current knowledge. Arch Gynecol Obstet. 2017;295(5):1061–77.
3. Severi FM, et al. Intrauterine growth retardation and fetal cardiac function. Fetal Diagn Ther. 2000;15(1):8–19.
4. Cauli O, et al. Treatment with sildenafil prevents impairment of learning in rats born to pre-eclamptic mothers. Neuroscience. 2010;171(2):506–12.
5. Herraiz S, et al. Sildenafil citrate improves perinatal outcome in fetuses from pre-eclamptic rats. BJOG. 2012;119(11):1394–402.
6. Karasu E, et al. Endothelial dysfunction in the human umbilical artery due to preeclampsia can be prevented by sildenafil. Clin Exp Hypertens. 2012;34(2):79–85.
7. Maharaj CH, et al. Effects and mechanisms of action of sildenafil citrate in human chorionic arteries. Reprod Biol Endocrinol. 2009;7:34.
8. Miller SL, et al. The effects of sildenafil citrate (Viagra) on uterine blood flow and well being in the intrauterine growth-restricted fetus. Am J Obstet Gynecol. 2009;200(1):102 e1–7.
9. Nassar AH, et al. Effects of sildenafil in Nomega-nitro-L-arginine methyl ester-induced intrauterine growth restriction in a rat model. Am J Perinatol. 2012;29(6):429–34.
10. Pellicer B, et al. Haemodynamic effects of long-term administration of sildenafil in normotensive pregnant and non-pregnant rats. BJOG. 2011;118(5):615–23.
11. Ramesar SV, et al. Sildenafil citrate decreases sFlt-1 and sEng in pregnant l-NAME treated Sprague-Dawley rats. Eur J Obstet Gynecol Reprod Biol. 2011;157(2):136–40.
12. Samangaya RA, et al. A randomised, double-blinded, placebo-controlled study of the phosphodiesterase type 5 inhibitor sildenafil for the treatment of preeclampsia. Hypertens Pregnancy. 2009;28(4):369–82.
13. Sanchez-Aparicio P, et al. Effects of sildenafil on the fetal growth of guinea pigs and their ability to survive induced intrapartum asphyxia. Am J Obstet Gynecol. 2008;198(1):127 e1–6.
14. Sasser JM, Baylis C. Effects of sildenafil on maternal hemodynamics and fetal growth in normal rat pregnancy. Am J Physiol Regul Integr Comp Physiol. 2010;298(2):R433–8.
15. Satterfield MC, et al. Sildenafil citrate treatment enhances amino acid availability in the conceptus and fetal growth in an ovine model of intrauterine growth restriction. J Nutr. 2010;140(2):251–8.
16. Stanley JL, et al. Sildenafil citrate rescues fetal growth in the catechol-O-methyl transferase knockout mouse model. Hypertension. 2012;59(5):1021–8.
17. Turgut NH, et al. The effect of sildenafil on the altered thoracic aorta smooth muscle responses in rat pre-eclampsia model. Eur J Pharmacol. 2008;589(1–3):180–7.
18. Villanueva-Garcia D, et al. A systematic review of experimental and clinical studies of sildenafil citrate for intrauterine growth restriction and pre-term labour. J Obstet Gynaecol. 2007;27(3):255–9.
19. Wareing M, et al. Sildenafil citrate (Viagra) enhances vasodilatation in fetal growth restriction. J Clin Endocrinol Metab. 2005;90(5):2550–5.
20. Wareing M, et al. Phosphodiesterase-5 inhibitors and omental and placental small artery function in normal pregnancy and pre-eclampsia. Eur J Obstet Gynecol Reprod Biol. 2006;127(1):41–9.
21. Chen J, et al. Effect of L-arginine and sildenafil citrate on intrauterine growth restriction fetuses: a meta-analysis. BMC Pregnancy Childbirth. 2016;16:225.
22. Dastjerdi MV, Hosseini S, Bayani L. Sildenafil citrate and uteroplacental perfusion in fetal growth restriction. J Res Med Sci. 2012;17(7):632–6.
23. von Dadelszen P, et al. Sildenafil citrate therapy for severe early-onset intrauterine growth restriction. BJOG. 2011;118(5):624–8.
24. Trapani A Jr, et al. Comparison between transdermal nitroglycerin and sildenafil citrate in intrauterine growth restriction: effects on uterine, umbilical and fetal middle cerebral artery pulsatility indices. Ultrasound Obstet Gynecol. 2016;48(1):61–5.
25. Gluud C, et al. Hepatology may have problems with putative surrogate outcome measures. J Hepatol. 2007;46(4):734–42.
26. Trapani A Jr, et al. Perinatal and hemodynamic evaluation of sildenafil citrate for preeclampsia treatment: a randomized controlled trial. Obstet Gynecol. 2016;128(2):253–9.
27. Hrobjartsson A, et al. Bias due to lack of patient blinding in clinical trials. A systematic review of trials randomizing patients to blind and nonblind sub-studies. Int J Epidemiol. 2014;43(4):1272–83.
28. Savovic J, et al. Influence of reported study design characteristics on intervention effect estimates from randomized, controlled trials. Ann Intern Med. 2012;157(6):429–38.
29. Schulz KF, et al. Empirical evidence of bias. Dimensions of methodological quality associated with estimates of treatment effects in controlled trials. JAMA. 1995;273(5):408–12.
30. Sutton AJ, et al. Empirical assessment of effect of publication bias on meta-analyses. BMJ. 2000;320(7249):1574–7.
31. Ganzevoort W, et al. STRIDER: sildenafil therapy in dismal prognosis early-onset intrauterine growth restriction--a protocol for a systematic review with individual participant data and aggregate data meta-analysis and trial sequential analysis. Syst Rev. 2014;3:23.
32. Andrew Sharp CC, Jackson R, Harrold J, Turner MA, Kenny LC, Baker PN, Johnstone ED, Khalil A, von Dadelszon P, Papageorghiou AT, Alfirevic Z, on behalf of the STRIDER group. Maternal sildenafil for severe fetal growth restriction (STRIDER): a multicentre, randomised, placebo-controlled, double-blind trial. Lancet Child Adolesc Health Published Online. 2017. Lancet Child Adolesc Health. 2018;2(2):93-102.
33. Ganzevoort, W., Dutch STRIDER study protocol. 2014. https://clinicaltrials.gov/ct2/show/NCT02277132.
34. Boutron I, et al. Reporting methods of blinding in randomized trials assessing nonpharmacological treatments. PLoS Med. 2007;4(2):e61.
35. Hrobjartsson A, et al. Observer bias in randomised clinical trials with binary outcomes: systematic review of trials with both blinded and non-blinded outcome assessors. BMJ. 2012;344:e1119.
36. Hrobjartsson A, et al. Observer bias in randomized clinical trials with measurement scale outcomes: a systematic review of trials with both blinded and nonblinded assessors. CMAJ. 2013;185(4):E201–11.
37. Hrobjartsson A, et al. Observer bias in randomized clinical trials with time-to-event outcomes: systematic review of trials with both blinded and non-blinded outcome assessors. Int J Epidemiol. 2014;43(3):937–48.
38. Page MJ, et al. Empirical evidence of study design biases in randomized trials: systematic review of meta-epidemiological studies. PLoS One. 2016;11(7):e0159267.

39. Savovic J, et al. Influence of reported study design characteristics on intervention effect estimates from randomised controlled trials: combined analysis of meta-epidemiological studies. Health Technol Assess. 2012;16(35):1–82.
40. Wood L, et al. Empirical evidence of bias in treatment effect estimates in controlled trials with different interventions and outcomes: meta-epidemiological study. BMJ. 2008;336(7644):601–5.
41. WMA Declaration of Helsinki – ethical principles for medical research involving human subjects. 2013. [Available from: https://www.wma.net/policies-post/wma-declaration-of-helsinki-ethical-principles-for-medical-research-involving-human-subjects/.
42. Wet medisch-wetenschappelijk onderzoek met mensen. 2017; Available from: http://wetten.overheid.nl/BWBR0009408/2017-03-01.
43. Medical Research Involving Human Subjects (Medical Research (Human Subjects) Act. Available from: https://english.ccmo.nl/investigators/legal-framework-for-medical-scientific-research.
44. De experimentenwet (7 mei 2004) en Uitvoeringsbesluit KB (koninklijk besluit) (30 juni 2004). Available from: https://www.fagg-afmps.be/en/human_use/medicines/medicines/research_development/clinical_trials.
45. Guideline for Good Clinical Practice. Available from: http://www.ich.org/fileadmin/Public_Web_Site/ICH_Products/Guidelines/Efficacy/E6/E6_R1_Guideline.pdf.
46. Kessler KM. The CONSORT statement: explanation and elaboration. Consolidated Standards of Reporting Trials. Ann Intern Med. 2002;136(12):926–7 author reply 926-7.
47. L.M. Leijser, G.M., S.M. Mulder-de Tollenaer, Aanbeveling neonatale neuroimaging versie 1.5. 2015.
48. Bancalari E, Claure N. Definitions and diagnostic criteria for bronchopulmonary dysplasia. Semin Perinatol. 2006;30(4):164–70.
49. Finer NN, Bates R, Tomat P. Low flow oxygen delivery via nasal cannula to neonates. Pediatr Pulmonol. 1996;21(1):48–51.
50. Jobe AH, Bancalari E. Bronchopulmonary dysplasia. Am J Respir Crit Care Med. 2001;163(7):1723–9.
51. Walsh MC, et al. Safety, reliability, and validity of a physiologic definition of bronchopulmonary dysplasia. J Perinatol. 2003;23(6):451–6.
52. Walsh MC, et al. Impact of a physiologic definition on bronchopulmonary dysplasia rates. Pediatrics. 2004;114(5):1305–11.
53. Kindergeneeskunde, N.V.v. Richtlijn Bronchopulmonaire dysplasie. 2013.
54. (NOG), N.O.G. Richtlijn Prematuren Retinopathie. 2012.
55. Bell MJ, et al. Neonatal necrotizing enterocolitis. Therapeutic decisions based upon clinical staging. Ann Surg. 1978;187(1):1–7.
56. Bayley N. Bayley Scales of Infant and Toddler Development—Third Edition: Administration manual; 2006.
57. (NVOG), N.V.v.O.e.G., Hypertensieve aandoeningen in de zwangerschap. 2011.
58. Saphier CJ, Repke JT. Hemolysis, elevated liver enzymes, and low platelets (HELLP) syndrome: a review of diagnosis and management. Semin Perinatol. 1998;22(2):118–33.
59. Onderzoeksprotocol The Dutch STRIDER https://doi.org/10.5281/zenodo.56148.
60. Lees CC, et al. Two-year neurodevelopmental and intermediate perinatal outcomes in infants with very preterm fetal growth restriction (TRUFFLE): a randomised trial. Lancet. 2015;385(9983):2162–72.
61. Lees C, et al. Perinatal morbidity and mortality in early-onset fetal growth restriction: cohort outcomes of the trial of randomized umbilical and fetal flow in Europe (TRUFFLE). Ultrasound Obstet Gynecol. 2013;42(4):400–8.
62. Dutch STRIDER: Data Monitoring Committee Charter https://doi.org/10.5281/zenodo.56147.
63. O'Brien PC, Fleming TR. A multiple testing procedure for clinical trials. Biometrics. 1979;35(3):549–56.
64. Ashby D, Machin D. Stopping rules, interim analyses and data monitoring committees. Br J Cancer. 1993;68(6):1047–50.
65. Jakobsen JC, et al. The thresholds for statistical and clinical significance - a five-step procedure for evaluation of intervention effects in randomised clinical trials. BMC Med Res Methodol. 2014;14:34.
66. Wetterslev J, Jakobsen JC, Gluud C. Trial sequential analysis in systematic reviews with meta-analysis. BMC Med Res Methodol. 2017;17(1):39.
67. Jakobsen, J.C., Systematic reviews of randomised clinical trials examining the effects of psychotherapeutic interventions versus 'no intervention' for major depressive disorder and a randomised trial examining the effects of 'third wave' cognitive therapy versus mentalization-based treatment for major depressive dis-order. Ph.D. Thesis, 2013: p. 1–62.
68. Goodman SN. Introduction to Bayesian methods I: measuring the strength of evidence. Clin Trials. 2005;2(4):282–90 discussion 301–4, 364–78.
69. McNutt LA, et al. Estimating the relative risk in cohort studies and clinical trials of common outcomes. Am J Epidemiol. 2003;157(10):940–3.
70. Jakobsen JC, et al. When and how should multiple imputation be used for handling missing data in randomised clinical trials - a practical guide with flowcharts. BMC Med Res Methodol. 2017;17(1):162.

Ready to submit your research? Choose BMC and benefit from:

- fast, convenient online submission
- thorough peer review by experienced researchers in your field
- rapid publication on acceptance
- support for research data, including large and complex data types
- gold Open Access which fosters wider collaboration and increased citations
- maximum visibility for your research: over 100M website views per year

At BMC, research is always in progress.

Learn more biomedcentral.com/submissions

BMC
