# Supplementary material for: Maternal Sildenafil vs Placebo in Pregnant Women With Severe Early-Onset Fetal Growth Restriction: A Randomized Clinical Trial
Source: JAMA Netw Open. 2020 Jun 17;3(6):e205323. doi: 10.1001/jamanetworkopen.2020.5323 (PMC7301225; doi:10.1001/jamanetworkopen.2020.5323)
Supplement: Supplement 2. — eAppendix 1. Letter of DSMB Regarding Result of Interim Analysis eAppendix 2. Differences Between the Predefined Statistical Analysis Plan and the Final Analysis eAppendix 3. Trial Sequential Analyses eTable 1. Doppler Measurements at Randomization and First Measurement More Than 24 Hours After Start of Medication eTable 2. Types of Pulmonary Hypertension Within Live Born Neonates in the Dutch STRIDER Trial per Randomization Allocation eTable 3. Characteristics of Neonates With and Without Pulmonary Hypertension eTable 4. Non–Context Specific Serious Adverse Events eTable 5. Side Effects of Study Medication as Reported by the Participants eTable 6. Primary Causes of Neonatal Death eTable 7. Congenital Anomalies eReferences [file jamanetwopen-e205323-s002.pdf]

Supplementary Online Content

Pels A, Derks J, Elvan-Taspinar A, et al; the Dutch STRIDER Trial Group. Maternal sildenafil vs placebo in pregnant women with severe early-onset fetal growth restriction: a randomized clinical trial. *JAMA Netw Open*. 2020;3(6):e205323. doi:10.1001/jamanetworkopen.2020.5323

eAppendix 1. Letter of DSMB Regarding Result of Interim Analysis

eAppendix 2. Differences Between the Predefined Statistical Analysis Plan and the Final Analysis

eAppendix 3. Trial Sequential Analyses

eTable 1. Doppler Measurements at Randomization and First Measurement More Than 24 Hours After Start of Medication

eTable 2. Types of Pulmonary Hypertension Within Live Born Neonates in the Dutch STRIDER Trial per Randomization Allocation

eTable 3. Characteristics of Neonates With and Without Pulmonary Hypertension

eTable 4. Non–Context Specific Serious Adverse Events

eTable 5. Side Effects of Study Medication as Reported by the Participants

eTable 6. Primary Causes of Neonatal Death

eTable 7. Congenital Anomalies

eReferences

This supplementary material has been provided by the authors to give readers additional information about their work.

eAppendix 1. Letter of DSMB Regarding Result of Interim Analysis

consortium 2.0

DSMB Recommendation STRIDER Study (NL)

July 19, 2018

On July 11th 2018, the NVOG DSMB for the STRIDER study met to discuss the planned (planned at completion of 175 subjects) interim analysis for the second time. At the first discussion on May 23rd it was decided to make sure safety data from the STRIDER study was as complete as possible and to obtain (mainly safety) data of the two completed sister studies (UK and New Zeeland and Australia) before arriving at a final recommendation. The NVOG DSMB allowed only a maximum time of one month for completion of the data to minimise risks to participants. For the meeting on July 11th the interim analysis was updated, with data cut off July 5th.

The interim analysis included endpoint data from 183 patients, of whom for 182 (89 on placebo, 93 on sildenafil) the primary endpoint of was available.

The DSMB has carefully evaluated the totality of data based on the interim analysis report and included the results from the two sister studies in its considerations, however not deviating from the principle that the interim data from the present STRIDER study should guide its recommendation. The DSMB also requested several additional analyses to ensure its interpretation of the data was correct.

The DSMB recommends stopping recruitment and treatment for the STRIDER study at the earliest possible occasion that allows proper communication to the participants and other stakeholders. The main consideration for the DSMB to recommend stopping is that there is serious concern that sildenafil may cause harm to the newborn children, whereas given the results of 182 children it is extremely unlikely that any benefit can be shown on the primary endpoint of *intact neonatal survival until term age* if the trial is continued to its completion (conditional power < 0.01).

The main potential harm observed is persistent pulmonary hypertension, with an incidence of 17/64 (26.6%) on sildenafil and 3/58 (5.2%) on placebo.

The following results summarize the key results underlying the DSMB recommendation.

	Sildenafil		*Placebo*	
Intact neonatal survival until term age	*32/93*	*34.4%*	*39/89*	*43.8%*
Death prior to discharge	*19/71*	*26.8%*	*9/63*	*14.3%*
Persistent pulmonary hypertension	*17/64*	*26.6%*	*3/58*	*5.2%*

Concluding, the DSMB recommends not to continue the trial from a safety perspective, while sufficient data appears to be available to assess benefit - risk of sildenafil for this treatment objective.

Prof Kit C.B. Roes, chair

© 2020 Pels A et al. *JAMA Network Open.*

eAppendix 2. Differences Between the Predefined Statistical Analysis Plan and the Final Analysis

We submitted the statistical analysis plan of this trial before the discontinuation due to the interim analysis and was in a second review stage at discontinuation of the trial.

The inclusion criterium 'PlGF < 5th percentile' was removed. In clinical practice, it appeared that no participating centres performed measurement of PlGF during standard patient car, but only for purpose of research. In clinical practice this pre-specified inclusion criterium was not known for potentially eligible patients.

In Table 1 we pre-defined the outcomes 'highest completed educational level mother', 'highest educational level father/partner' and 'language spoken at home'. These variables are important for the neurodevelopmental outcomes at two years of age. Since these variables are not collected systematically during pregnancy, we had a high proportion of missing data. During the neurodevelopmental assessment at two years, these variables will be collected systematically and we will report them in the publication of the long-term outcomes.

The variables 'Female sex (%)' and 'PlGF < 5th percentile of the reference value (%)' were added to Table 1, since this variable was considered to be clinically relevant.

In Table 2 and 3 the relative risk with 95% confidence interval was added for all outcomes.

The variable "Neonate born during maternal administration of intravenous magnesium sulphate (%)" has been removed from Table 2, since this outcome was not systematically registered and collected within the trial.

The variables 'Preterm birth < 28 weeks', 'Preterm birth < 37 weeks', 'Birth weight < 10th percentile', 'Birth weight < 3rd percentile', 'Apgar score 5 minutes < 7' and 'Cord blood gas pH < 7.10' were added to table 2 and 3, since these variables were considered to be clinically relevant and are part of the identified core outcome set[1].

© 2020 Pels A et al. *JAMA Network Open.*

The variable 'Birthweight' was added to Table 3 after peer review of the manuscript on request of the reviewers.

In Table 4 a post-hoc analysis adjusting for the imbalance in fetal sex was added, since this imbalance was observed in the results.

The pre-planned subgroup analysis, comparing placental growth factor (PlGF) < 25th percentile of all samples of the study population and PlGF ≥ 25th percentile of all samples of the study population, has not been reported, since most available PlGF outcomes were low. Therefore, this subgroup analysis was considered to have minimal clinical impact.

The pre-defined subgroup analysis, comparing participants who had a fetus or neonate without any congenital anomaly that could either explain the small fetal size in hindsight or would have a likely impact on the primary outcome, was changed to a sensitivity analysis. This analysis was wrongly defined as a subgroup analysis, since we do not test the effect of sildenafil in the participants who had a fetus or neonate with any congenital anomaly that could either explain the small fetal size in hindsight or would have a likely impact on the primary outcome.

Table 6 and Table S1 were added, since we considered it important to present the different types of pulmonary hypertension, the association between pulmonary hypertension, neonatal death and treatment allocation. We also considered it important to explore the pregnancy characteristics of the neonates that did and did not develop pulmonary hypertension in order to improve interpretation of the results.

Table S2, S3 and S4 were moved to the appendix for purpose of readability of the manuscript.

In the statistical analysis plan we planned to use random intercept models for all primary analyses to account for a center effect. However, due to a lower power after early discontinuation of the trial the models did not converge for all outcomes, we decided to use fixed-effect models and not to account for a center effect.

© 2020 Pels A et al. *JAMA Network Open.*

The two statisticians that independently conducted the statistical analyses, were deblinded due to the fact that pulmonary hypertension was not excluded from the data set and via their knowledge of the results of the interim analysis.

© 2020 Pels A et al. *JAMA Network Open.*

eAppendix 3. Trial Sequential Analyses

We followed our published statistical analysis plan.[2]

Primary neonatal outcome

When we applied the data of the interim analysis of the Dutch STRIDER trial to the Trial Sequential Analysis program we observed that futility had been reached regarding the primary neonatal outcome of intact neonatal survival (Figure 1).

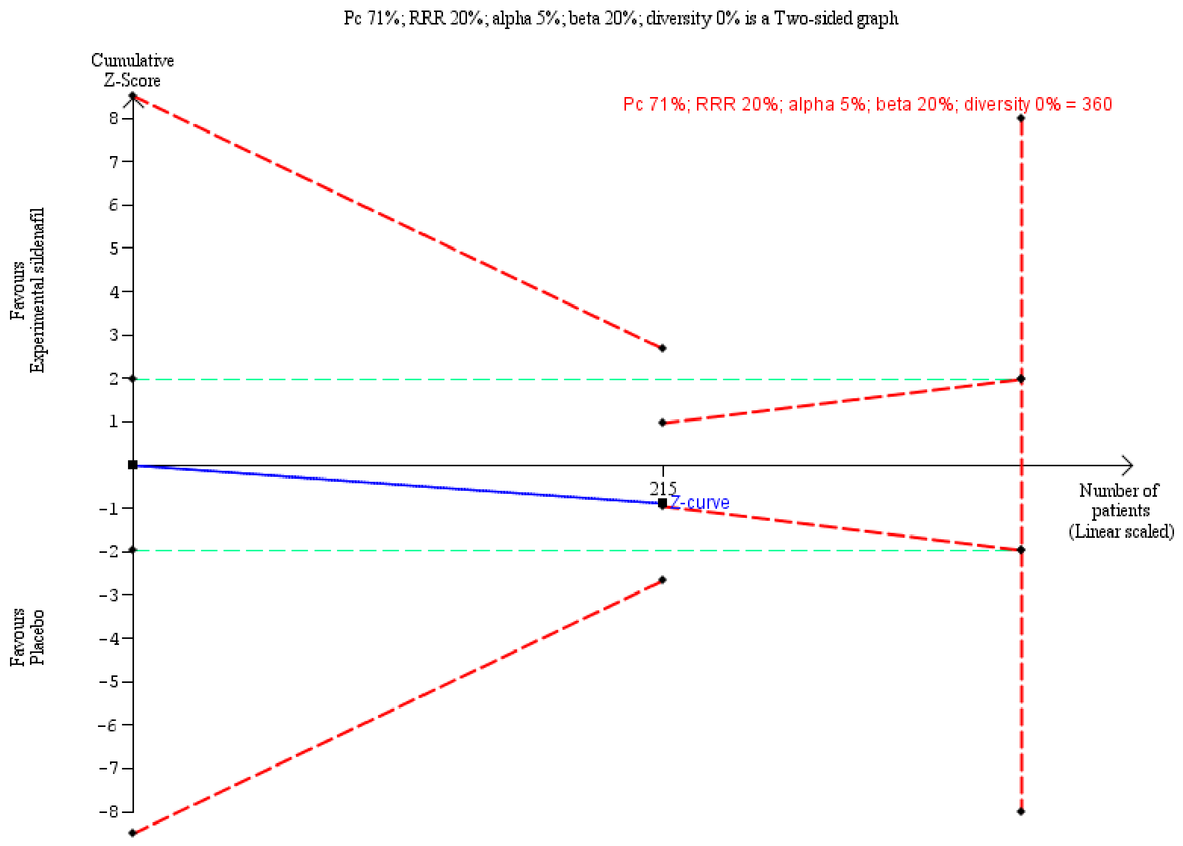

Figure 1. Trial Sequential Analysis of the interim data of the primary outcome from the Dutch STRIDER trial. The cumulative Z curve after 215 participants have been randomized and followed up penetrates the monitoring boundaries for futility. The required sample size of 360 participants is calculated based on the anticipated proportion of neonates with the primary outcome of 71%; a relative risk reduction of 21%; alpha of 5%; beta of 20%; and 0% diversity.

© 2020 Pels A et al. *JAMA Network Open.*

When we conducted a Trial Sequential Analysis of the two published STRIDER trials plus the interim data from the Dutch Strider trial we found again that the cumulative Z curve entered the futility area (Figure 2). The observed relative risk (RR) is 1.05 with 95% confidence interval (CI) of 0.90 to 1.20. We are able to demonstrate futility down to intervention effects of 16% relative risk reduction (data not shown). For smaller intervention effects we could not demonstrate futility (data not shown).

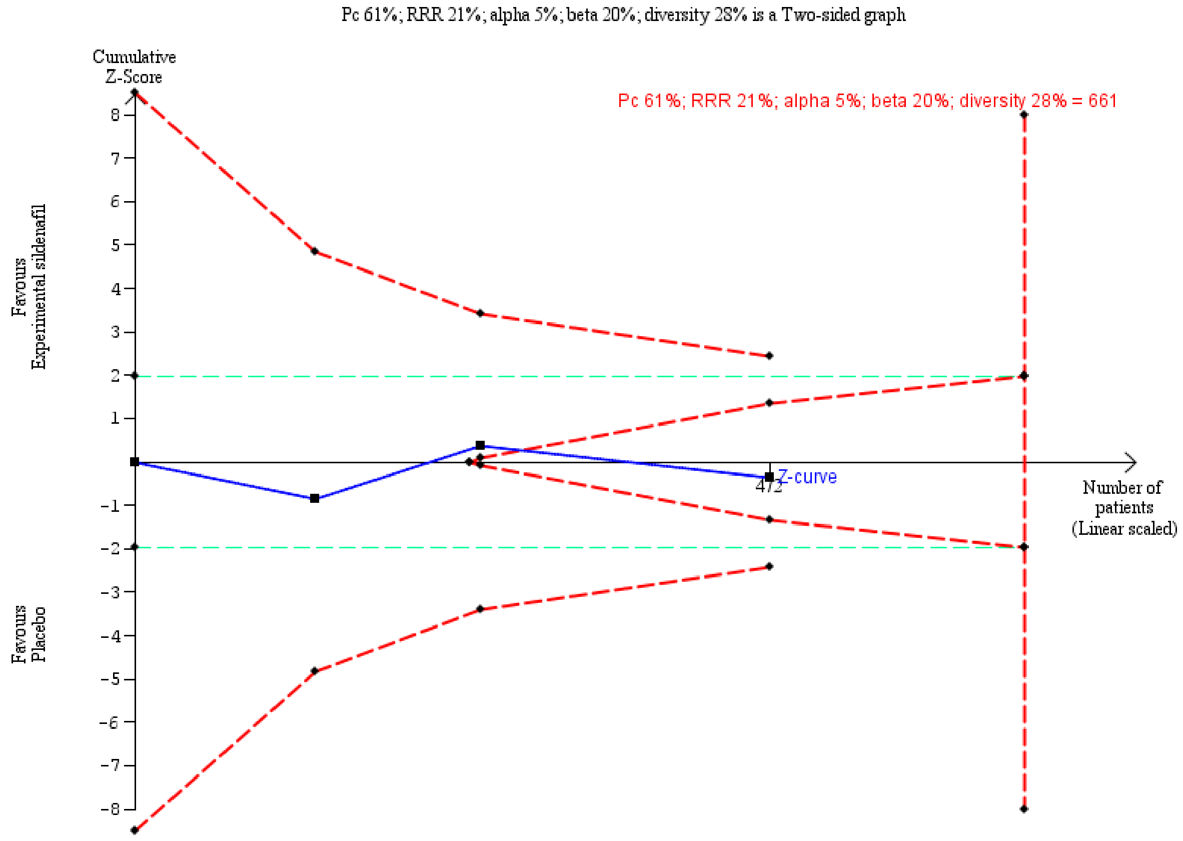

Figure 2. Trial Sequential Analysis of the primary outcome from the two published STRIDER trials[3,4] plus the interim data from the Dutch STRIDER trial. The cumulative Z curve after addition of the Dutch trial participants enters the area of futility. The diversity-adjusted required information size of 661 participants is calculated based on the observed proportion of neonates with the primary outcome of 61%; a relative risk reduction of 21%; alpha of 5%; beta of 20%; and the observed diversity 28%.

© 2020 Pels A et al. *JAMA Network Open.*

eTable 1. Doppler Measurements at Randomization and First Measurement More Than 24 Hours After Start of Medication

	Sildenafil (n= 108)		Placebo (n= 107)		P value*
	At randomization	After start medication	At randomization	After start medication	
Mean PI uterine artery	1.76 (±0.66)	1.29 (±0.61)	1.70 (±0.61)	1.65 (±1.03)	0.90
Mean PI umbilical artery	1.89 (±0.79)	1.97 (±1.04)	2.37 (±3.95)	1.92 (±0.86)	0.62
Mean PI middle cerebral artery	1.57 (±0.56)	1.51 (±0.41)	1.46 (±0.47)	1.58 (±0.44)	0.06
Mean PI ductus venosus	0.79 (±0.32)	0.88 (±0.40)	1.06 (±0.99)	0.73 (±0.32)	0.80

Plus-minus values are standard deviations (SD)

PI = pulsatility index

* P value for the comparison of the difference in Doppler measurements at randomization and after start of medication between the sildenafil and placebo groups.

© 2020 Pels A et al. *JAMA Network Open.*

eTable 2. Types of Pulmonary Hypertension Within Live Born Neonates in the Dutch STRIDER Trial per Randomization Allocation

	Sildenafil (n=85)		Placebo (N=78)	
	Neonatal death (n=21)	Survival to discharge (n=64)	Neonatal death (n=11)	Survival to discharge (n=67)
Total pulmonary hypertension	10	6	3	1
PPHN	7	3	1	1
PH associated with sepsis	1	1	0	0
Late-onset PH associated with (developing) BPD	1	0	1	0
PPHN followed by late-onset PH associated with (developing) BPD	1	2	1	0

PPHN = Persistent Pulmonary Hypertension of the Neonate. PH = pulmonary hypertension. BPD = bronchopulmonary dysplasia.

The total number of neonates in the sildenafil group with pulmonary hypertension compared with the placebo group was 16/85 (19%) versus 4/78 (5%); RR 3.67; 95% CI 1.28 to 10.51; P=0.008.

© 2020 Pels A et al. *JAMA Network Open.*

eTable 3. Characteristics of Neonates With and Without Pulmonary Hypertension

	Neonates with PH (n=20)	Neonates without PH (n=143)	P value
GA at randomization (weeks + days) (median, IQR)	24 + 6 (23 + 1 to 25 + 4)	25 + 3 (24 + 0 to 26 + 4)	0.05
EFW at randomization (g) (median, IQR)	475 (323 to 536)	518 (377 to 645)	0.06
Absent or reversed EDF at randomization (%)	9 (45.0%)	35 (24.5%)	0.04
GA at delivery (weeks + days) (median, IQR)	26 + 6 (26 +0 to 28 + 2)	29 + 4 (27 + 6 to 34 + 1)	<0.0001
Birth weight (g) (median, IQR)	573 (484 to 650)	805 (639 to 1460)	<0.0001
Female sex (%)	7 (35.0%)	71 (49.7%)	0.24
Maternal pre-eclampsia or HELLP (%)	6 (30.0%)	34 (23.8%)	0.58

PH = pulmonary hypertension; GA = gestational age; EFW = estimated fetal weight; EDF = end-diastolic flow; HELLP = Hemolysis Elevated Liver enzymes Low Platelets

© 2020 Pels A et al. *JAMA Network Open.*

eTable 4. Non–Context Specific Serious Adverse Events

	Sildenafil (n= 108)	Placebo (n= 107)
**Maternal**		
Spontaneous preterm labor	0	3
Admission due to threatened preterm labor	1	1
Hospital admission due to headache	1	0
Admission due to vaginal blood loss	1	0
Admission for observation after fall on abdomen	1	0
Hospital admission due to pain, itch and hypertension	1	0
Admission due to suboptimal CTG, headache and itch	0	1
Asymptomatic hyponatremia and hyperkaliemia	0	1
Hydronephrosis for which double J stent placement	0	1
Infected hematoma cesarean scar for which opening of the wound and antibiotic treatment	1	0
Admission due to fever and malaise, due to wound infection combined with upper airway infection, for which antibiotic treatment	1	0
Subcutaneous hematoma after cesarean section for which re-laparotomy	1	0
Severe HELLP postpartum, or antiphospholipid syndrome flare	1	0
Hospital admission due to delirious postpartum	1	0
Hospital admission due to endometritis postpartum	0	1
**Fetal/neonatal**		
Atypical hemorrhagic lesions in the wall of lateral ventricles	0	1
Progressive cholestasis	0	1
Intrahepatic portal venous shunt	1	0

© 2020 Pels A et al. *JAMA Network Open.*

Death 18 months postpartum due to cardiogenic shock based on sepsis	1	0

SAE = serious adverse event; CTG = cardiotocography; HELLP = hemolysis elevated liver enzymes low platelets

© 2020 Pels A et al. *JAMA Network Open.*

eTable 5. Side Effects of Study Medication as Reported by the Participants

	**Sildenafil (n= 103)**	**Placebo (n= 100)**
One or more side effects (%)	25 (24.3%)	9 (9.0%)
Headache (%)	13 (12.6%)	4 (4.0%)
Flushing (%)	8 (7.8%)	1 (1.0%)
Congested nose (%)	5 (4.9%)	0
Nausea/ reflux (%)	4 (3.9%)	3 (3.0%)
Bleeding nose (%)	3 (2.9%)	0
Fatigue (%)	3 (2.9%)	0
Abdominal pain (%)	2 (1.9%)	2 (2.0%)
Edema (%)	2 (1.9%)	1 (1.0%)
Hot sensation (%)	1 (1.0%)	1 (1.0%)
Dry mouth/throat (%)	1 (1.0%)	1 (1.0%)
Diarrhea (%)	1 (1.0%)	1 (1.0%)
Dizziness (%)	1 (1.0%)	0
Stuffiness (%)	1 (1.0%)	0
Redness face (%)	1 (1.0%)	0
Spider naevi (%)	1 (1.0%)	0
Skin rash (%)	1 (1.0%)	0
Blood blister (%)	1 (1.0%)	0
Night sweating (%)	0	1 (1.0%)
Muscle ache (%)	1 (1.0%)	0
Blurry vision (%)	0	1 (1.0%)
Reduced fetal movements (%)	1 (1.0%)	0
Braxton hicks contractions (%)	1 (1.0%)	0
Palpitations (%)	1 (1.0%)	0
Numb feeling in legs (%)	0	1 (1.0%)
Malaise (%)	1 (1.0%)	0

© 2020 Pels A et al. *JAMA Network Open.*

eTable 6. Primary Causes of Neonatal Death

Cause of neonatal death	Sildenafil (n = 21)	Placebo (n = 11)
Sepsis/infection	7 (33.3%)	2 (18.2%)
Pulmonary hypertension	2 (9.5%)	2 (18.2%)
Necrotizing enterocolitis	3 (14.3%)	1 (9.1%)
Bronchopulmonary dysplasia	1 (4.8%)	1 (9.1%)
Intestinal perforation	2 (9.5%)	0
Intestinal ischemia	0	2 (18.2%)
Intracerebral hemorrhage	1 (4.8%)	1 (9.1%)
Non-intervention due to non-viability/dismal prognosis	2 (9.5%)	0
Result of a congenital anomaly	1 (4.8%)	1 (9.1%)
Choking	1 (4.8%)	0
Pericardial tamponade	0	1 (9.1%)
Respiratory distress syndrome	1 (4.8%)	0

© 2020 Pels A et al. *JAMA Network Open.*

eTable 7. Congenital Anomalies

Diagnosis	Further information
Silver Russell syndrome	DNA-confirmed
Silver Russell syndrome	DNA-confirmed
Silver Russel syndrome	Neonatal death, 4 major clinical criteria, not DNA-confirmed
Hartnup's disease	Macrocephaly, receding skull seams, abnormality of the ear (relative macrocephaly due to brain sparing.
Atresia of the ductus venosus	Neonatal death, finding confirmed at autopsy
Mosaicism trisomy 16	Amniocentesis after randomization. Termination of pregnancy after finding.
Multiple congenital anomalies, no confirmed syndrome	Neonatal death with multiple anomalies confirmed at autopsy. Esophageal atresia with tracheo-esophageal fistula, tethered cord, hypertelorism
ADA-SCID (adenosine deaminase deficiency - severe combined immune deficiency)	Neonatal death due to late necrotizing enterocolitis and sepsis, in the setting of a primary T-cell immunodeficiency (DNA-confirmed).
Multiple congenital anomalies, no confirmed syndrome	Single umbilical artery, hemivertebrae, wide fontanelle. No confirmed DNA-diagnosis
Unbalanced translocation	Amniocentesis after randomization. Termination of pregnancy after finding.
Congenital nephropathy RMDN1 gene mutation	Neonatal death, homozygous mutation of RMND1 gene (p.Leu393Val).

© 2020 Pels A et al. *JAMA Network Open.*

Multiple congenital anomalies, no confirmed syndrome	Large neurocranium, arthrogryposis, hypospadia, syndactyly. No confirmed DNA-diagnosis.

© 2020 Pels A et al. *JAMA Network Open.*

eReferences

1. Healy P, Gordijn SJ, Ganzevoort W, et al. A Core Outcome Set for the prevention and treatment of fetal GROwth restriction: deVeloping Endpoints: the COSGROVE study. *American journal of obstetrics and gynecology*. May 29 2019.
2. Pels A, Jakobsen JC, Ganzevoort W, et al. Detailed statistical analysis plan for the Dutch STRIDER (Sildenafil TheRapy In Dismal prognosis Early-onset fetal growth Restriction) randomised clinical trial on sildenafil versus placebo for pregnant women with severe early onset fetal growth restriction. *Trials*. Jan 11 2019;20(1):42.
3. Groom KM, McCowan LM, Mackay LK, et al. STRIDER NZAus: A multicentre randomised controlled trial of sildenafil therapy in early-onset fetal growth restriction. *BJOG : an international journal of obstetrics and gynaecology*. Feb 19 2019;126(8):997-1006.
4. Sharp A, Cornforth C, Jackson R, et al. Maternal sildenafil for severe fetal growth restriction (STRIDER): a multicentre, randomised, placebo-controlled, double-blind trial. *Lancet Child Adolesc Health*. Feb 2018;2(2):93-102.

© 2020 Pels A et al. *JAMA Network Open.*
