# Supplementary material for: Maternal Sildenafil vs Placebo in Pregnant Women With Severe Early-Onset Fetal Growth Restriction: A Randomized Clinical Trial
Source: JAMA Netw Open. 2020 Jun 17;3(6):e205323. doi: 10.1001/jamanetworkopen.2020.5323 (PMC7301225; doi:10.1001/jamanetworkopen.2020.5323)
Supplement: Supplement 3. — Dutch STRIDER Trial Group Members [file jamanetwopen-e205323-s003.pdf]

Supplemental Online Content: Nonauthor Collaborators
*First name, last name, and suffix (if applicable) are required and will appear in PubMed.

***Group Name(s):**							
***First Name and Middle Initial(s)**	***Last Name**	***Suffix (eg, Jr, III)**	Academic Degrees	Institution	Location (city, state/province, country)	Role or Contribution, eg, chair, principal investigator	Group (if more than 1 Group listed in the byline) and/or Subgroup (eg, Steering Committee)
Joris A. M.	van der Post		MD, PhD	Department of Obstetrics and Gynecology, Amsterdam UMC, University of Amsterdam	Amsterdam, The Netherlands	Concept and design	
Katayoun	Taghavi		MD	Department of Obstetrics and Gynecology, Inselspital Bern, Frauenklinik	Bern, Switzerland	Local investigator	
Ben W.	Mol		MD, PhD	Department of Obstetrics and Gynecology, Monash University, Monash Medical Centre	Clayton, Australia	Concept and design	
Harry	van Goor		MD, PhD	Department of Pathology and Medical Biology, section Pathology, University Medical Center Groningen, University of Groningen	Groningen, The Netherlands	Local investigator	
Rolf M. F.	Berger		MD, PhD	Department of Pediatric Cardiology, Beatrix Children's Hospital, University Medical Center Groningen, University of Groningen	Groningen, The Netherlands	Local investigator	
Willem P.	de Boode		MD, PhD	Department of Neonatology, Radboud University Medical Center, Radboud Institute for Health Sciences, Amalia Children's Hospital	Nijmegen, The Netherlands	Local investigator	
Danilo	Gavilanes		MD, PhD	Department of Neonatology, Maastricht University Medical Center	Maastricht, The Netherlands	Local investigator	

Supplemental Online Content: Nonauthor Collaborators
*First name, last name, and suffix (if applicable) are required and will appear in PubMed.

***First Name and Middle Initial(s)**	***Last Name**	***Suffix (eg, Jr, III)**	Academic Degrees	Institution	Location (city, state/province, country)	Role or Contribution, eg, chair, principal investigator	Group (if more than 1 Group listed in the byline) and/or Subgroup (eg, Steering Committee)
Arno F. J.	van Heijst		MD, PhD	Department of Neonatology, Radboud University Medical Center	Nijmegen, The Netherlands	Local investigator	
Elisabeth M. W.	Kooi		MD, PhD	Division of Neonatology, Beatrix Children's Hospital, University Medical Center Groningen, University of Groningen	Groningen, the Netherlands	Local investigator	
Petra	Lemmers		MD, PhD	Department of Neonatology, Wilhelmina Children's Hospital, University Medical Center Utrecht	Utrecht, the Netherlands	Local investigator	
Enrico	Lopriore		MD, PhD	Leiden University Medical Center, Department of Neonatology	Leiden, The Netherlands	Local investigator	
Susanne M.	Mulder-de Tollenaer		MD, PhD	Department of Pediatrics, Isala Hospital	Zwolle, the Netherlands	Local investigator	
Hendrik	Niemarkt		MD, PhD	Department of Neonatology, Maxima Medisch Centrum	Veldhoven, The Netherlands	Local investigator	
Irwin K.M.	Reiss		MD, PhD	Department of Pediatrics, Division of Neonatology, Erasmus UMC	Rotterdam, the Netherlands	Local investigator	
Sinno H.P.	Simons		MD, PhD	Department of Pediatrics, Division of Neonatology, Erasmus UMC Rotterdam, Sophia Children's hospital	Rotterdam, the Netherlands	Local investigator	
Mirjam M.	van Weissenbruch		MD, PhD	Department Pediatrics/Intenive Care Neonatology, Amsterdam UMC, Location Vumc	Amsterdam, The Netherlands	Local investigator	
