# Supplementary material for: Maternal Sildenafil vs Placebo in Pregnant Women With Severe Early-Onset Fetal Growth Restriction: A Randomized Clinical Trial
Source: JAMA Netw Open. 2020 Jun 17;3(6):e205323. doi: 10.1001/jamanetworkopen.2020.5323 (PMC7301225; doi:10.1001/jamanetworkopen.2020.5323)
Supplement: Supplement 4. — Data Sharing Statement [file jamanetwopen-e205323-s004.pdf]

# Data Sharing Statement

Pels. Maternal Sildenafil vs Placebo in Pregnant Women With Severe Early-Onset Fetal Growth Restriction. *JAMA Netw Open*. Published June 17, 2020.
doi:10.1001/jamanetworkopen.2020.5323

**Data**
**Data available:** Yes
**Data types:** Deidentified participant data
**How to access data:** j.w.ganzevoort@amsterdamumc.nl
**When available:** With publication

**Supporting Documents**
**Document types:** Statistical/analytic code
**How to access documents:** j.w.ganzevoort@amsterdamumc.nl
**When available:** With publication

**Additional Information**
**Who can access the data:** researchers whose proposed use of the data has been approved
**Types of analyses:** for a specified purpose
**Mechanisms of data availability:** with a signed data access agreement
